# Artificial morphogen-mediated differentiation in synthetic protocells

**DOI:** 10.1038/s41467-019-11316-4

**Published:** 2019-07-25

**Authors:** Liangfei Tian, Mei Li, Avinash J. Patil, Bruce W. Drinkwater, Stephen Mann

**Affiliations:** 1 Centre for Protolife Research and Centre for Organized Matter Chemistry, School of Chemistry, University of Bristol, Bristol, BS8 1TS UK; 2 Faculty of Engineering, Queens Building, University of Bristol, Bristol, BS8 1TR UK

**Keywords:** Materials chemistry, Soft materials, Biomimetics

## Abstract

The design and assembly of artificial protocell consortia displaying dynamical behaviours and systems-based properties are emerging challenges in bottom-up synthetic biology. Cellular processes such as morphogenesis and differentiation rely in part on reaction-diffusion gradients, and the ability to mimic rudimentary aspects of these non-equilibrium processes in communities of artificial cells could provide a step to life-like systems capable of complex spatiotemporal transformations. Here we expose acoustically formed arrays of initially identical coacervate micro-droplets to uni-directional or counter-directional reaction-diffusion gradients of artificial morphogens to induce morphological differentiation and spatial patterning in single populations of model protocells. Dynamic reconfiguration of the droplets in the morphogen gradients produces a diversity of membrane-bounded vesicles that are spontaneously segregated into multimodal populations with differentiated enzyme activities. Our results highlight the opportunities for constructing protocell arrays with graded structure and functionality and provide a step towards the development of artificial cell platforms capable of multiple operations.

## Abstract (web summary)

The ability to mimic aspects of cellular process that rely on reaction-diffusion gradients could provide a step to building life-like systems capable of complex behaviour. Here the authors demonstrate morphological differentiation in coacervate micro-droplets.

## Introduction

Building arrays of artificial cell-like entities capable of contact or non-contact modes of interaction is an emerging challenge in bottom-up synthetic biology. A wide range of synthetic protocells in the form of single compartmentalized microstructures have been constructed and used as biomimetic models of cell-free gene expression^1–4^, enzyme activity in crowded^5,6^, or confined^7,8^ environments, artificial cytoskeleton reconstitution^9,10^, membrane gating^11^, motility^12–14^ and membrane growth and division^15–17^. These design strategies have been extended to the construction of multi-compartmentalized microsystems comprising nested^18–22^ or host-guest^23–25^ arrangements of synthetic protocells and exploited in the programmed release of molecular cargos, positional assembly of functional components, and spatiotemporal regulation of enzyme cascade reactions. In contrast, much less progress has been made in the assembly of protocell consortia displaying dynamical behaviours and systems-based emergent properties. Contact-dependent behaviours have been demonstrated by the use of physical or chemical adhesion in clusters of closely packed protocells. For example, prototissues capable of membrane protein-mediated electrical and chemical communication^26,27^ or reversible contractibility and mechanochemical transduction^28^ have been prepared using three-dimensional printing of lipid-coated water-in-oil emulsion droplets or programmed chemical ligation of bio-orthogonal proteinosomes, respectively. Transient contacts between synthetic protocells displaying complementary surface properties have been explored as steps toward the onset of artificial predation^29^, phagocytosis^30^ and parasitism^25^ in dispersed binary protocell populations. Alternatively, non-contact modes of chemical signalling within communities of protocells have been demonstrated in dispersions of lipid vesicles containing gene circuitry^31^, in binary populations of vesicles and proteinosomes^32^, between suspensions of colloidosomes^33^ and between 2D arrays of immobilized coacervate micro-droplets^34^. Chemical communication between artificial and living cells has also been explored^35–38^.

In contrast to the above studies, there are relatively few reports on artificial protocell systems that emulate cellular differentiation along morphogen gradients during developmental biology. Processes such as morphogenesis are regulated in part by signalling cues associated with chemical gradients, and mimicking rudimentary aspects of this process in synthetic protocells could provide a step towards the spontaneous physical and chemical differentiation of protocell consortia into multimodal populations of different types of artificial cells. In this regard, recent studies have used chemical gradients and reaction-diffusion systems for the structuring of matter and materials^39,40^, operation of chemical networks^41,42^, programming of artificial cells^43,44^ and control of signalling and differentiation in emulsion-based multi-compartmentalized gene circuits^45^. Here, we use chemical gradients comprising unidirectional or counter-directional flows of artificial morphogens to induce spatial and functional differentiation in immobilized protocell arrays consisting of several thousands of initially identical membrane-free coacervate micro-droplets. The coacervate droplets are formed at the nodal points of an acoustic standing wave pressure field^46^, and then exposed to externally generated morphogen gradients. The latter are produced by injecting surface complexation/membrane-forming molecules that dynamically reconfigure the protocells according to the local concentration of the advancing near-planar chemical diffusion front. As a consequence, the uniform population of coacervate droplets spontaneously differentiates into a multimodal consortium of up to five different types of membrane-bounded micro-compartments that we broadly classify as “vesicles”. By using intersecting chemical gradients generated under different morphogen molar ratios we demonstrate that a range of organized protocell consortia can be produced under non-equilibrium conditions. We show that encapsulation of horseradish peroxidase within the coacervate droplets leads to differentiated protocell communities that exhibit spatially dependent enzymatic responses when exposed to identical substrate concentrations principally due to morphogen-induced changes in vesicle membrane permeability. Overall our results highlight the potential of using spatiotemporal responses to chemical gradients for the spontaneous morphogen-mediated differentiation of single protocell populations and provide a step towards integrating reactive flow systems into the design of synthetic protocell communities with spatially organized functional diversity^46^.

## Results

### Morphological transformations under non-diffusive conditions

Coacervate micro-droplets are widely studied as membrane-free molecularly crowded protocell models exhibiting diverse functions and interactivity^2,3,5,6,24,25,29,47–49^. Recently, we reported that coacervate droplets prepared by liquid phase separation in charge-balanced mixtures of polydiallydimethylammonium chloride (PDDA) and adenosine 5^/^-triphosphate (ATP) undergo spontaneous re-structuration under equilibrium conditions into membrane-bounded coacervate vesicles^50^. Reconfiguration of the droplets was induced by complexation of a water-soluble polyanionic polyoxometalate (POM; sodium phosphotungstate, [PW_11_O_39_]^7-^) with PDDA polycations present at the droplet surface. As a consequence, the unstructured coacervate micro-droplets transformed into a three-tiered synthetic protocell consisting of a semi-permeable negatively charged POM/PDDA outer membrane typically 600 nm in thickness, a 2–5-μm-wide sub-membrane viscoelastic coacervate shell capable of sequestering organic dyes, proteins and nanoparticles, and an internal aqueous lumen often tens of micrometres in diameter. Transformation of the PDDA/ATP coacervate droplets into the POM/coacervate vesicles was associated with the ingress of water and concomitant volume expansion, which in turn were attributed to an increase in osmotic pressure across the POM/PDDA membrane due to the impermeability of displaced [ATP]^4-^ anions. Consequently, encapsulated proteins were protected from proteases in the external medium, exploited in spatially localized enzyme cascade reactions and used for chemically signalling between different POM/coacervate vesicle populations^50^.

Given these observations, we prepared acoustically formed 2D uniform arrays of immobilized PDDA/ATP coacervate droplets and initially investigated whether the above-mentioned coacervate-to-vesicle transitions could be modulated by changes in POM concentration under non-diffusive equilibrium conditions. Two-dimensional square-lattice arrays of micro-droplets with uniform sizes (mean diameter, 67.8 ± 7.1  μm) and a centre-to-centre spacing of 110 μm were prepared (Supplementary Figs. 1 and 2, and Methods)^46^. Fluorescence microscopy images of the coacervate phase labelled with 2^/^,3^/^-*O*-(2,4,6-trinitrophenyl)adenosine-5^/^-triphosphate (TNP-ATP) or rhodamine-labelled poly(allylamine hydrochloride) (RITC-PAH) (Supplementary Fig. 3) indicated that the micro-droplets were formed in situ at the nodal regions of the acoustic pressure field and strongly bound to the underlying PEGylated glass substrate (Supplementary Fig. 4). A stirred aqueous solution of the POM clusters was then added to the acoustic trapping chamber (20 × 20 mm; total volume = 1 mL; central viewing window, 4 × 5 mm) such that the pre-organized micro-droplets were simultaneously exposed to the same additive concentration under non-diffusive equilibrium conditions.

**Fig. 1:**
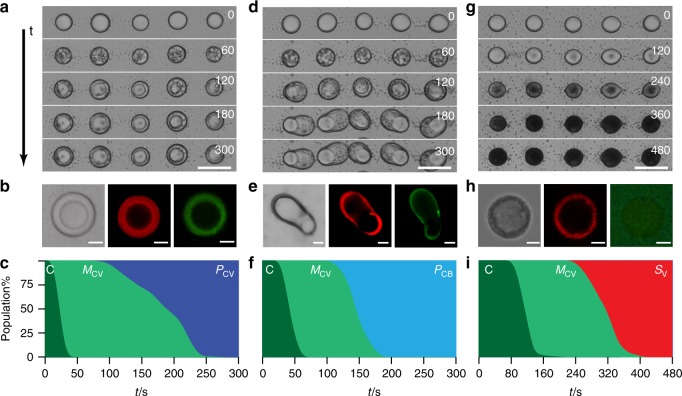
Morphological transformations under equilibrium conditions. **a** Time (*t*)-dependent optical microscopy images recorded from an acoustically formed array of PDDA/ATP coacervate micro-droplets at 0, 60, 120, 180 and 300 s after addition of a stirred solution of sodium phosphotungstate (POM, final concentration 2 mM). Scale bar, 100 µm. **b** Representative optical microscopy image (left), and RITC-PAH-doped (middle, red fluorescence) and TNP-ATP-doped (right, green fluorescence) fluorescence microscopy images of a single-POM/coacervate vesicle. Scale bars, 20 µm. **c** Corresponding area plot showing time-dependent changes in the numbers of native coacervate micro-droplets (**C**, dark green), multi-compartmentalized coacervate vesicles (**M**_**CV**_, light green) and spherical POM/coacervate vesicles (**P**_**CV**_, dark blue) over 300 s. Changes in populations are shown as percentage of total. **d**–**f**)As for **a**–**c**) but in the presence of a lower final POM concentration (1 mM) showing transformation of the coacervate micro-droplets into POM/coacervate vesicles with balloon-like morphology (**P**_**CB**_, light blue) via a series of **M**_**CV**_ intermediates (see images at *t* = 60 s). Scale bars, 100 µm (**d**) and 20 µm (**e**). **g**–**i** As for **a**–**c** but without POM and in the presence of SDS micelles (final concentration, 20 mM). Optical microscopy images are recorded at 0, 120, 240, 360 and 480 s (**g**). The coacervate droplets transform into ATP-depleted SDS/PDDA vesicles (**S**_**V**_) (**h**). Transformation to **S**_**V**_ (red area in (**i**)) occurs sequentially over 300 s via a **M**_**CV**_ intermediate. Scale bars, 100 µm (**g**) and 5 µm (**h**). Source data are provided as a Source Data file

Optical and fluorescence microscopy images showed that the immobilized droplets were uniformly transformed in the presence of sodium phosphotungstate (2 mM) over a period of *ca*. 4 min into spherical membrane-bounded POM/coacervate vesicles comprising an optically dense POM/PDDA outer shell, coacervate sub-membrane layer and a single low contrast aqueous lumen (Fig. 1a and Supplementary Fig. 5). Restructuring of the POM/coacervate vesicles occurred at each nodal point and produced minimal disruption of the acoustically formed array. Time-lapse optical microscopy images revealed a series of intermediate structures consisting of spherical multi-compartmentalized coacervate vesicles containing multiple water micro-droplets (Supplementary Fig. 6). With time, the internalized water droplets coalesced to produce a single aqueous filled lumen that compressed the coacervate phase against the POM/PDDA membrane (Fig. 1b and Supplementary Movie 1). The corresponding time-dependent area plots for the fraction of each individual population within the 2D droplet array showed a linear sequence of morphological transformations (Fig. 1c).

Transformation of the coacervate droplets to spherical membrane-bounded POM/coacervate vesicles was kinetically inhibited at very low or high sodium phosphotungstate concentrations. For example, the multi-compartmentalized coacervate vesicles exhibited prolonged lifetimes at POM concentrations less than 0.5 mM to produce a viable morphological type that did not transition into the spherical coacervate vesicles (Supplementary Fig. 6 and Supplementary Movie 2). Similarly, the time required for the coacervate droplets to transform into the POM/coacervate vesicles was extended to ~12 min at 20 mM due to osmotically induced collapse of the droplets on addition of the high concentration of POM clusters (Supplementary Figs. 7 and 8, and Supplementary Movie 3).

Undertaking the above equilibrium experiments at a POM concentration of 1 mM produced spherical multi-compartmentalized coacervate vesicles that subsequently became extended preferentially along the vertical direction over a period of *ca*. 5–10 min to produce membrane-bounded POM/coacervate vesicles with a balloon-shaped morphology and single aqueous micro-compartment (Fig. 1d, e). The transitions proceeded sequentially and were irreversible (Fig. 1f). Anisotropic growth of the vesicles at the reduced POM concentration, which occurred vertically followed by collapse of the elongated microstructures onto the substrate, was attributed to the inability of the reduced osmotic pressure to overcome the droplet adhesion force (Supplementary Movie 4). Significantly, no elongated vesicles were observed in control experiments undertaken with coacervate droplet suspensions containing various amounts of the polyanionic POM clusters (Supplementary Fig. 9). Taken together, the above results indicated that the transformation of acoustically formed PDDA/ATP coacervate micro-droplets in the presence of a homogeneous solution of polyanionic POM clusters produced three distinct types of membrane-bounded coacervate vesicles as the concentration of the additive was increased from 0.5 to 2 mM (Supplementary Fig. 10).

Similar experiments were undertaken to assess the influence of negatively charged sodium dodecylsulfate (SDS; CH_3_(CH_2_)_11_OSO_3_^−^) micelles on restructuring individual PDDA/ATP coacervate micro-droplets under non-diffusive equilibrium conditions. Exposure to SDS concentrations above the critical micelle concentration (CMC = 8.2 mM) over a period of *ca*. 8 min resulted in the formation of a semi-permeable surfactant-polyelectrolyte shell (Supplementary Figs. 11 and 12) and irreversible transformation of the immobilized coacervate droplets to membrane-bounded single-compartment water-filled SDS/PDDA vesicles that exhibited increased optical contrast (Fig. 1g and Supplementary Movie 5). Significantly, labelling the micro-droplets with fluorescent TNP-ATP or RITC-PAH indicated that ATP was replaced by SDS as the shell developed such that there was essentially no ATP, and hence no coacervate phase, in the SDS/PDDA vesicles (Fig. 1h and Supplementary Fig. 13). The corresponding time-dependent changes in the fraction of each individual population within the 2D droplet array indicated that the SDS micelles induced a linear sequence of morphological transformations involving coacervate-containing intermediates comprising multiple water micro-droplets (Fig. 1i). In contrast, no distinct membrane or significant loss of ATP was observed for coacervate micro-droplets exposed to SDS concentrations below the CMC (Supplementary Fig. 13), indicating that SDS molecules alone were ineffective as structure-inducing agents.

### Differentiation in unidirectional morphogen gradients

**Fig. 2:**
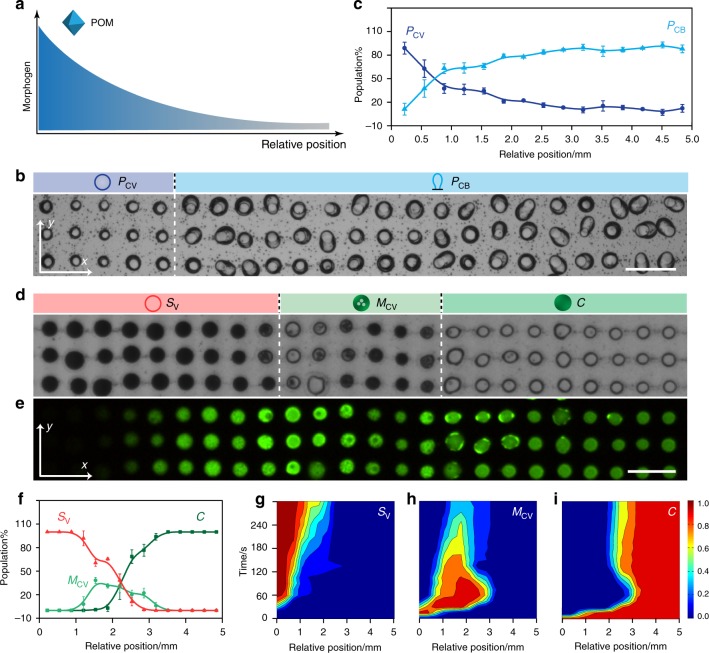
Protocell differentiation in unidirectional gradients. **a** Graphical representation of a unidirectional POM chemical gradient (*x-*axis). Similar experiments were undertaken with a SDS chemical gradient. **b** Optical microscopy image of an acoustically patterned array of PDDA/ATP coacervate droplets observed in the viewing window after exposure to a unidirectional POM diffusion gradient advancing along the *x*-axis. Images were recorded after no further changes in morphology were observed (15 min). Only a small section (3 × 23 droplet grid) of the array in the observation window is displayed. Sodium phosphotungstate (POM) is introduced into the chamber from the left-hand side of the image (50 µL, 12.5 mM, final POM concentration, 0.625 mM). Dashed white line marks the interface between the two differentiated protocell populations; spherical and balloon-shaped POM/coacervate vesicles, **P**_**CV**_ (dark blue circle) and **P**_**CB**_ (light blue graphic), respectively. Scale bar, 200 µm. (**c**) Plot showing changes in the population number densities of **P**_**CV**_ and **P**_**CB**_ morphological types along the chemical gradient (from left to right along the *x-*axis) after POM-mediated differentiation under conditions as described in (**b**); number of counted protocells, *n* = 1500. **d** Optical microscopy image of a PDDA/ATP coacervate droplet array after exposure to a SDS chemical gradient advancing along the *x*-axis. Images were recorded after no further changes in morphology were observed (30 min). Only a thin section (3 × 24 droplet grid) of the array viewed in the observation window is displayed. SDS is introduced into the chamber (50 µL, 50 mM; final concentration, 2.5 mM) from the left-hand side of the image. Three spatially separated populations consisting of ATP-depleted SDS/PDDA vesicles (**S**_**V**_; red circle; left side), multi-compartmentalized coacervate vesicles (**M**_**CV**_; green circle with dots; centre) and native coacervate droplets (**C**; green filled circle; centre) are observed. Dashed white lines mark the interfaces between the differentiated protocell populations; scale bar, 200 µm. **e** Corresponding fluorescence microscopy image for (**d**); droplets were initially prepared with green fluorescent TNP-ATP. **f** Plot showing changes in the population number densities of **S**_**V**_, **M**_**CV**_ and **C** morphological types along the morphogen gradient (from left to right along the *x*-axis) after SDS-mediated differentiation under conditions as described in (**d**); number of counted protocells, *n* = 1500. **g**–**i** 2D plots showing spatial and temporal distributions of morphological types **S**_**V**_ (**g**) **M**_**CV**_ (**h**) and **C** (**i**) produced under conditions described in (**d**); colour scale represents percentage of a given population. Total number of counted protocells, *n* = 1500. The **C** to **M**_**CV**_ transition occurs across regions of relatively high SDS concentration (left and centre-left) after approximately 30 s, followed by subsequent transformation to **S**_**V**_ in areas of highest SDS concentration (far left). Coacervate droplets remain unchanged in domains of relatively low SDS concentration (right side). Source data are provided as a Source Data file. Error bars represent the standard deviation of the statistics count of the different lines of different protocells (*n* = 3)

Given that POM clusters and SDS micelles were both capable of concentration-dependent restructuring of the PDDA/ATP coacervate micro-droplets under equilibrium conditions, we sought to exploit these additives as artificial morphogens for generating spatiotemporal patterns of chemical/physical differentiation in homogenous populations of initially identical model protocells. For this, we prepared an acoustically formed square array of immobilized PDDA/ATP micro-droplets, switched off the acoustic pressure field and then injected an aqueous solution of sodium phosphotungstate or SDS into the device specifically from one edge of the chamber (Supplementary Fig. 1) to produce a unidirectional chemical gradient across the observation window of the device (Fig. 2a). Computer simulations of the corresponding diffusion gradients (see Methods) were determined in the absence of a reaction-induced depletion of each morphogen and confirmed that although a circular diffusion front was generated initially at the point of injection, the coacervate droplets housed within the observation chamber were exposed locally to a near-planar diffusion front that advanced specifically along the *x* direction (Supplementary Figs. 14 and 15). The simulated times associated with the arrival of the additives in the observation window were commensurate with the experimental values (*ca*. 3 min) for the known morphogen concentrations. In general, the simulated concentration gradients of POM or SDS increased with time to a threshold value, which progressively increased as the amount of morphogen added at the simulated injection point increased.

We systematically varied the initial concentrations of the POM clusters or SDS micelles to determine the optimum conditions required for observing a spatiotemporal response within the central viewing window (4 × 5 mm; grid size, 33 × 45 droplets) positioned *ca*. 10 mm from the point of injection. Typically, an induction time of 2–5 min was required for the individual morphogens to diffuse into the viewing window. We then used optical and fluorescence microscopy to monitor time-dependent morphogen gradient-mediated changes in the individual coacervate micro-droplets as a function of their spatial positions in the array. The images revealed a progressive change in morphology specifically along rows of micro-droplets aligned parallel but not perpendicular to the direction of diffusion (*x*-axis). For example, injection of an aqueous solution of the POM clusters to give a final concentration across the entire chamber after equilibration of 0.625 mM resulted in the spontaneous differentiation of the homogeneous population of coacervate droplets into a binary population of spherical (26%) and balloon-shaped (74%) membrane-bounded POM/coacervate vesicles (Fig. 2b, c and Supplementary Movie 6). The differentiated populations were separated spatially along the morphogen diffusion gradient such that the domain of balloon-shaped protocells was positioned further along the *x*-axis than the population of spherical vesicles in accordance with the decrease in POM concentrations along the chemical gradient.

Injecting a higher concentration of the polyanionic clusters shifted the interface between the binary population distribution further along the *x*-axis as the morphogen concentration gradient was increased across the viewing chamber until only a single population of spherical POM/coacervate vesicles was observed in the observation window (Supplementary Fig. 16). In each case, leaving the experiments to run for longer periods of time such that the reaction-diffusion field extended well beyond the viewing chamber and the concentration gradient decreased to zero did not show any changes in the spatially differentiated populations, indicating that the morphological transformations were irreversible.

Artificial morphogen-mediated differentiation of a homogeneous population of immobilized coacervate droplets was also observed in the presence of a unidirectional diffusion gradient of SDS micelles. For example, an array of spatially segregated droplets and vesicles comprising three distinct zones along the diffusion direction was observed in the viewing window after injection of a SDS solution equivalent to a final concentration of 2.5 mM (Fig. 2d, e and Supplementary Movie 7). Specifically, a domain of coacervate droplets positioned closest to the injection point of the morphogen transformed into an organized population of ATP-depleted SDS/PDDA vesicles, while arrays of multi-compartmentalized coacervate vesicles in various stages of transition and grids of native coacervate droplets were observed in the central and right-hand side regions of the observation window, respectively (Fig. 2f). The results were consistent with a SDS diffusion gradient that crossed the CMC threshold value (*ca*. 8 mM) specifically within the observation chamber. Increasing the injected SDS concentration (3.5 mM at equilibration) shifted the local concentrations above the CMC across the entire viewing window such that a single population of SDS/PDDA vesicles was observed (Supplementary Fig. 17). Plots of the time-dependent changes in the fraction of each stable population produced within the observation window (Supplementary Fig. 18) were consistent with a progressive sequence of morphological transformations that were analogous to those observed for POM- or SDS-mediated transitions under non-diffusive conditions (see Fig. 1c, f, i). The corresponding spatiotemporal profiles were also consistent with an established morphogen gradient across the observation window, particularly for SDS, which showed a clear consecutive pathway with regard to the time and spatial position of the morphological transformations along the diffusion direction (Fig. 2g–i). In contrast, the dynamics associated with POM-mediated differentiation were more complex due to the concentration-dependent kinetic inhibition of the coacervate-to-coacervate vesicle transition (Supplementary Fig. 8). As a consequence, coacervate droplets that were exposed to a shallow concentration gradient comprising POM levels commensurate with formation of the balloon-shaped vesicles underwent transformation initially in areas furthest away from the injection edge due to lower levels of kinetic inhibition in regions containing slightly lower concentrations of the morphogen (Supplementary Fig. 19 and Supplementary Movie 6). Thus, although the final spatial distribution of the populations was consistent with a well-defined POM concentration gradient from left to right in the observation window, the temporal sequence associated with the transformations often occurred in a contrary direction.

### Protocell differentiation in opposing morphogen gradients

**Fig. 3:**
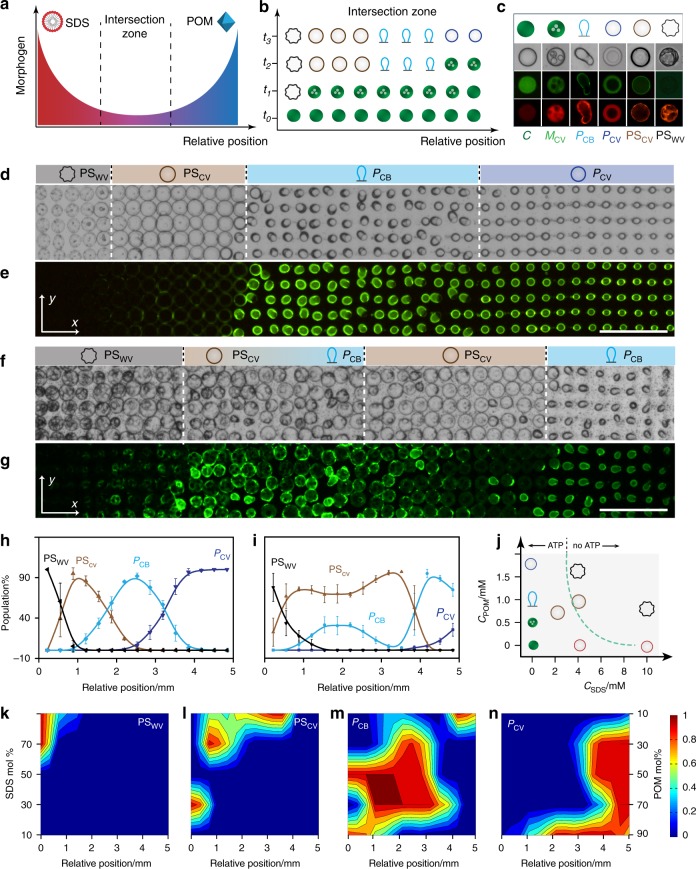
Protocell differentiation in opposing gradients. **a** Graphical representation showing counter-directional SDS (left, red) and POM (right, blue) chemical gradients along the *x*-axis. **b** Graphic showing possible sequence of morphogen-mediated transformations in the intersection zone shown in (**a**) for a single row of PDDA/ATP coacervate droplets (green filled circles, time *t* = 0). The various spatiotemporal responses produce a row of differentiated protocells with different morphologies (see (**c**) for details). Relative positions are along the *x*-axis; *t*_*n*_ = time intervals. **c** Panel of protocell morphological types produced in the SDS/POM intersection zone displaying representative graphics (row 1) and corresponding optical (row 2) and fluorescence microscopy images (row 3, TNP-ATP green fluorescence; row 4, RITC-PAH red fluorescence). From left to right; native coacervate droplet (**C**, filled green circle), multi-compartmentalized coacervate vesicle (**M**_**CV**_, green circle with dots), balloon-shaped POM/coacervate vesicle (**P**_**CB**_, (light blue graphic), spherical POM/coacervate vesicle (**P**_**CV**_, dark blue circle), POM/SDS/coacervate vesicle (**PS**_**CV**_, red circle) and POM/SDS wrinkled vesicle (**PS**_**WV**_, black graphic). **d**, **e** Optical (**d**) and corresponding fluorescence microscopy (**e**) images showing a 2D array of differentiated protocells viewed in the observation window after intersection of an opposing reaction-diffusion gradient of SDS (from left to right) and POM (from right to left) at an initial SDS : POM molar ratio of 2.3. Four spatially distinct populations are observed (white dashed lines). Images were recorded after no further changes in morphology were observed (30 min); displayed grid size, 5 × 42. **f**, **g** As for **d**, **e**, respectively, but for an initial SDS : POM morphogen ratio of 9.0 showing a spatially interpenetrating community. Images were recorded after no further changes in morphology were observed (20 min); displayed grid size, 5 × 42; scale bar 500 µm. **h**, **i** Plots showing correlated changes in relative number densities of four different types of differentiated protocells (% population) with spatial position along the direction (*x*-axis) of opposing morphogen gradients produced at SDS : POM molar ratios of 2.3 (**h**) and 9.0 (**i**). **j** Plot showing a landscape of protocell morphological types produced at different SDS : POM molar ratios under non-diffusive equilibrium conditions. Graphics corresponding to the different forms shown in **c** except for the SDS/PDDA vesicle (**S**_**V**_; red circle). All the forms develop irreversibly via a **M**_**CV**_ intermediate except for **PS**_**WV**_ which is derived from **PS**_**CV**_. Dashed line indicates the presence or absence of a coacervate phase (with/without ATP, respectively). **k**–**n** 2D plots showing the spatial distributions for final populations of **PS**_**WV**_ (**k**), **P**_**CV**_ (**l**), **P**_**CB**_ (**m**) and **PS**_**CV**_ (**n**) morphological types produced in opposing morphogen gradients prepared by injection of different amounts of SDS and POM (% values). Colour scale represents percentage of a given population. Source data are provided as a Source Data file. Error bars represent the standard deviation of the statistics count of the different lines of different protocells (*n* = 3)

Given that homogeneous arrays of initially identical PDDA/ATP coacervate micro-droplets could be differentiated into spatially separated populations of morphologically distinct synthetic vesicles in the presence of single-morphogen concentration gradients, we sought to increase the complexity of pattern formation by using opposing reaction-diffusion fields of POM clusters (sodium phosphotungstate) and SDS micelles co-aligned along the *x*-axis of the observation chamber in the acoustic device (Fig. 3a). For this, we subjected the micro-droplet arrays to a counter-flow of the two artificial morphogens under a range of different initial SDS: POM molar ratios, and used optical and fluorescence microscopy to assess the influence of the competing reaction-diffusion gradients on the spatiotemporal transformations associated with differentiation of the immobilized protocells (Fig. 3b).

Depending on the morphogen molar ratio, transformation of the coacervate droplets along the *x*-axis within the intersection zone resulted in up to five different irreversible morphological types (Fig. 3c), which were distributed at the lattice points of the array to produce discrete spatially organized populations in the central observation window In contrast, injecting homogenous mixtures of POM and SDS at different molar ratios under non-diffusive equilibrium conditions produced only single populations of the morphological types (Supplementary Fig. 20). In each case, transformations in the opposing morphogen gradients occurred via the formation and subsequent reconfiguration of multi-compartmentalized coacervate vesicles (**M**_**CV**_) typically over a period of 10 to 30 minutes. In general, regions exposed to intersecting morphogen gradients with low concentrations of SDS and high levels of POM were associated with spherical membrane-bounded POM/coacervate **v**esicles (**P**_**CV**_), while low concentrations of SDS and medium levels of POM produced membrane-bounded POM/coacervate balloon-shaped (**P**_**CB**_) vesicles. In contrast, droplets in contact with opposing gradients comprising competing levels of SDS micelles and POM clusters transformed into larger spherical POM/SDS/PDDA coacervate vesicles (**PS**_**CV**_) that were partially depleted in ATP and surrounded by a hybrid semi-permeable membrane (Supplementary Figs. 21 and 22). Higher levels of SDS in the areas of first contact restructured the coacervate micro-droplets into POM/SDS/PDDA wrinkled vesicles (**PS**_**WV**_) that were completely depleted in ATP and lacked an internal coacervate matrix (Supplementary Figs. 23 and 24). While single-morphogen transformations to the **P**_**CV**_ and **P**_**CB**_ types were routinely observed in the intersection zone, analogous transformations to SDS/PDDA vesicles were not generally recorded. We attributed this to the stronger interaction of the POM clusters with the PDDA/ATP coacervate droplets compared with SDS, which only displayed competitive activity above the critical micelle concentration (Supplementary Fig. 13).

Taken collectively, the above spatiotemporal transformations resulted in differentiation of the homogeneous population of initially identical coacervate micro-droplets into a spatially separated multimodal consortium comprising different types of membrane-bounded vesicles with the same chemical origin. For example, an initial SDS : POM morphogen molar ratio of 2.3 generated a tetra-modal distribution of hybrid (**PS**_**WV**_ and **PS**_**CV**_) and single-POM (**P**_**CB**_ and **P**_**CV**_) types within the SDS-enriched and POM-enriched regions of the intersection zone, respectively (Fig. 3d, e). Increasing the initial SDS : POM morphogen molar ratio to 9.0 also produced a tetra-modal distribution but consisting of **PS**_**WV**_**, PS**_**CV**_, **P**_**CB**_ and **P**_**CV**_ morphological forms (Fig. 3f, g). Measurements of the relative numbers of protocells against spatial position along the *x-*axis showed correlated distributions in the final population densities with a series of distinct or interpenetrating boundaries for arrays produced at ratios of 2.3 or 9.0, respectively (Fig. 3h, i). In the former case, the two morphogens appeared to be competitively matched at the interface between the opposing morphogen gradients to produce four well-delineated zones of the different morphological types. In contrast, the consortium produced at a ratio of 9.0 consisted of a small zone of **PS**_**WV**_ types nearest the entry of the SDS gradient, an extensive central region containing overlapping numbers of **PS**_**WV**_**, PS**_**CV**_ and **P**_**CB**_ forms with **PS**_**CV**_ being dominant throughout, and a small demarcated area of **P**_**CB**_ and **P**_**CV**_ microstructures closest to the input of the POM gradient.

To determine the conditions associated with the various transformations observed in the differentiated droplet arrays, we constructed a morphological landscape based on a systematic series of control experiments in which dispersions of PDDA/ATP coacervate droplets were incubated with different equilibrium concentrations of mixtures of SDS and POM (Fig. 3j and Supplementary Figs. 25–27). Typically, the **PS**_**CV**_ type was obtained when the final SDS and POM concentrations were below 3 mM and 1.75 mM, respectively. Mixtures of **PS**_**WV**_ and **PS**_**CV**_, or **PS**_**WV**_ alone, were observed for a range of POM concentrations provided that the final SDS concentrations were between 3 and 5 mM or above 5 mM, respectively. Given this information, we systematically varied the initial SDS : POM molar ratio used to establish the intersection zone in experiments involving opposing morphogen gradients and constructed 2D plots of the corresponding spatial distributions associated with the different final populations of the differentiated protocells (Fig. 3k–n). In general, tetra-modal, trimodal or binary distributions of the differentiated droplets were observed in the viewing window when the relative levels of added SDS were between 70–90%, 30–70% or 10–30%, respectively, of the total amount of injected morphogens. At very high SDS levels (>90%) the arrays consisted principally of spatially separated populations comprising combinations of SDS/PDDA vesicles (**S**_**V**_), **PS**_**WV**_, **PS**_**CV**_, **P**_**CB**_ and **P**_**CV**_ morphological types (Supplementary Fig. 28), while trimodal and bimodal populations of separated mixtures of **P**_**CB**_, **P**_**CV**_ and multi-compartmentalized coacervate vesicles were produced below 30% SDS (Supplementary Fig. 29).

**Fig. 4:**
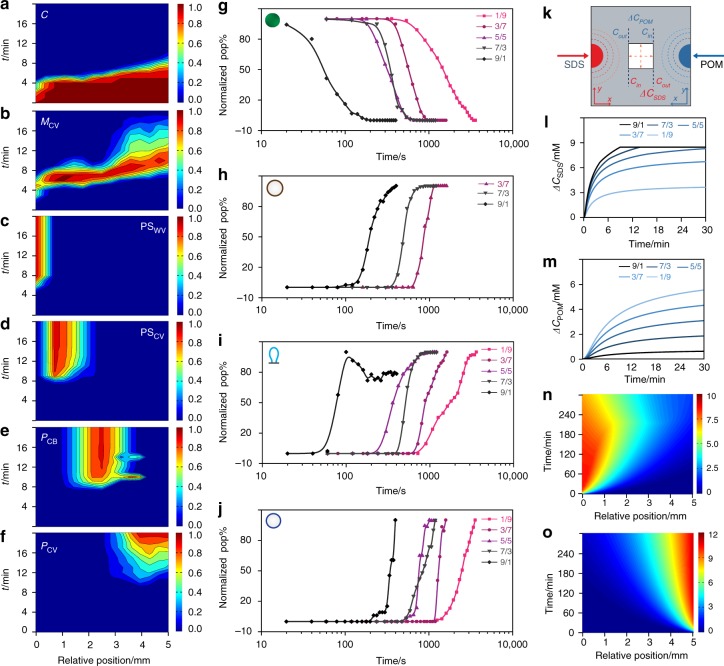
Population dynamics under opposing gradients. **a**–**f** 2D colour plots of the spatial and temporal population distributions of differentiated protocell types produced in an opposing morphogen gradient (initial SDS : POM molar ratio = 2.3 (70/30 mM)); **a** coacervate droplets (**C**); **b** multi-compartmentalized coacervate vesicles (**M**_**CV**_); **c** POM/SDS wrinkled vesicles (**PS**_**WV**_); **d** POM/SDS coacervate vesicles (**PS**_**CV**_); **e** balloon-shaped POM/coacervate vesicles (**P**_**CB**_) and **f** spherical coacervate vesicles (**P**_**CV**_). Colour scale represents number fraction of a given population; *n* = 1500. **g**–**j** Plots showing time-dependent changes in normalized populations of native coacervate droplets **C** (**g**), and differentiated protocells **PS**_**CV**_ (**h**), **P**_**CB**_ (**i**) and **P**_**CV**_ (**j**) in opposing morphogen gradients prepared under a range of initial SDS : POM molar ratios (9/1, 7/3, 5/5, 3/7). Data obtained from plots shown in **a**, **d**, **e**, **f**, respectively. **k** Simulation of opposing morphogen gradients in the central observation window (white square; 5 × 5 mm) of the acoustic trapping device. Changes in the SDS and POM concentrations perpendicular (*y*-axis) or parallel (*x*-axis) to the direction of diffusion are indicated (dashed red arrows in the centre square). The concentration gradients across the simulated area along the diffusion direction (*x*-axis) are defined as: *∆C* = *C*_*in*_ – *C*_*out*_. **l**, **m** Simulated time-dependent plots of the SDS concentration gradients (*∆C*_*SDS*_) (**l**) and POM concentration gradients (*∆C*_*POM*_) (**m**) established across the observation window and along the diffusion direction (*x*-axis) for various opposing SDS/POM morphogen gradients (90/10, 70/30, 50/50, 30/70 and 10/90 µL; 50 mM). The horizontal line shown in **l** represents the CMC of SDS. **n**, **o** Representative simulated 2D plots of the spatial and temporal distributions of SDS (**n**) and POM (**o**) concentrations for an opposing SDS/POM morphogen gradient of 2.3 (70/30 µL; 50 mM) along the *x*-axis in the centre of the acoustic trapping device. Colour scale represents morphogen concentrations in mM. Source data are provided as a Source Data file

Differentiation into the multimodal populations occurred via a dynamical sequence of droplet transformations (Supplementary Movies 8-10). In each case, changes in the corresponding population distributions were correlated both spatially and temporally (Supplementary Fig. 30). For example, at an initial SDS : POM molar ratio of 2.3 the progressive decrease in the coacervate droplet population across the array was matched initially with an increase in the numbers of multi-compartmentalized coacervate vesicles (**M**_**CV**_), which in turn underwent further morphological transformations depending on their spatial location within the opposing SDS/POM diffusion gradients (Supplementary Fig. 31). Specifically, transformation of the coacervate micro-droplets was first observed after 4 min in the SDS-enriched zone but then rapidly spread across the observation window such that an array of **M**_**CV**_ intermediates was produced within ~6 min (Fig. 4a, b). The multi-compartmentalized coacervate vesicles differentiated almost simultaneously into **PS**_**WV**,_ **PS**_**CV**_ and **P**_**CB**_ forms to produce three spatially discrete zones after *ca*. 8 min (Fig. 4c–e). In contrast, the **M**_**CV**_ to **P**_**CV**_ transformation within the POM-enriched intersection zone was kinetically delayed by approximately a further 2 min (Fig. 4f) prior to formation of a fourth segregated population. 2D spatiotemporal distribution plots for intersecting gradients produced at other SDS: POM ratios are shown in Supplementary Figs. 32-35.

Plots of the time-dependent changes in the normalized populations for each morphological type under a range of opposing morphogen gradients indicated that in each case the decrease in number of native coacervate micro-droplets and concomitant increase in the differentiated **PS**_**CV**_, **P**_**CB**_ and **P**_**CV**_ populations displayed similar sigmoidal decay/growth curves with an initial lag period, exponential decay/growth phase and final plateau stage (Fig. 4g–j). The period associated with decay of the coacervate population decreased as the mole fraction of SDS increased (Supplementary Fig. 36), consistent with the lower rate of diffusion of the SDS micelles compared with the POM clusters. Typically, times of 3000 and 180 s were observed for opposing gradients prepared at SDS : POM molar ratios of 0.11 and 9.0, respectively. As a consequence, differentiation of the homogeneous droplet array was forestalled at SDS concentrations at 30% or below, although once initiated, the rates of transformation for a given morphological type were essentially independent of the lag period and SDS : POM molar ratio (Fig. 4g–j).

We attributed the above results to the complex interplay between the opposing morphogen gradients. Computer simulations of the opposing SDS and POM diffusion profiles across the array under a range of SDS : POM molar ratios showed spatiotemporal profiles that intersected in the simulated observation window (Fig. 4k). The simulated SDS concentration gradients, which increased with the SDS : POM molar ratio, reached a steady state value over 30 min associated with attainment of the CMC threshold (Fig. 4l); by comparison, the simulated POM concentration gradients progressively increased in rate and amplitude over a period of 30 min as the SDS : POM molar ratio was decreased (Fig. 4m). As a consequence, the simulated 2D plots of the spatiotemporal distributions of SDS showed a modulated diffusion front while those for POM were essentially unperturbed by the presence of an opposing morphogen flux (Fig. 4n, o). This was attributed to secondary interactions between the counterflowing morphogens, which was modelled by incorporation of a POM-derived ionic strength dependence of the SDS CMC locally along the chemical gradient, as shown experimentally (Supplementary Fig. 37).

### Functional diversity in differentiated protocell consortia

**Fig. 5:**
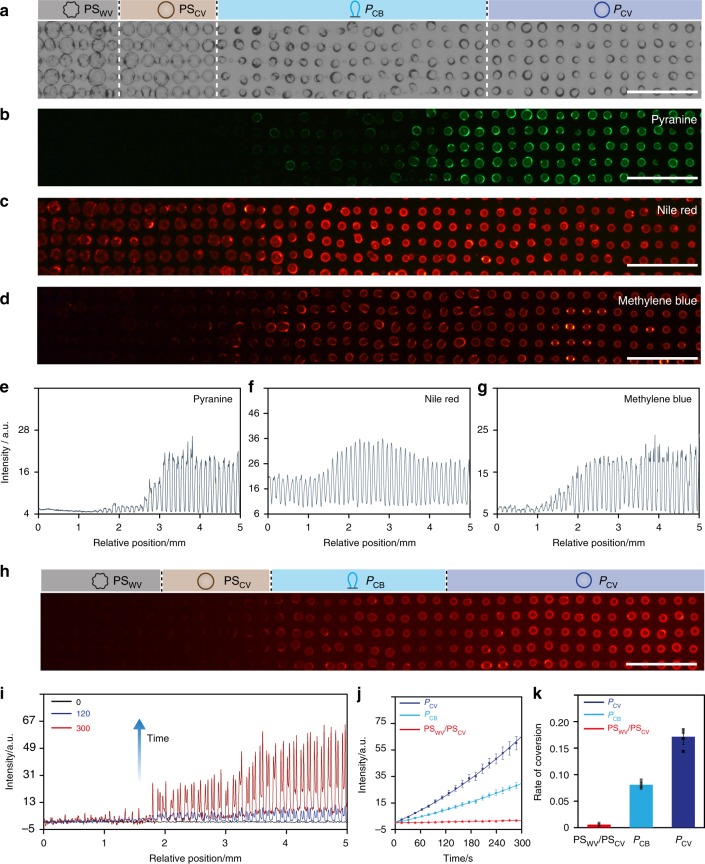
Functional diversity in differentiated protocell consortia. Representative optical (**a**) and corresponding fluorescence microscopy (**b**–**d**) images of 2D arrays of differentiated coacervate droplets comprising a spatially separated tetra-modal distribution of **PS**_**WV**_, **PS**_**CV**_, **P**_**CB**_ and **P**_**CV**_ (from left to right, see graphics) morphological forms after exposure to aqueous solutions of pyranine (negatively charged, **b)**, Nile Red (neutral, **c**) or methylene blue (positively charged, **d)**; scale bars, 500 µm. **e**–**g** Average fluorescence line intensity profiles recorded across the tetra-modal populations (spatial distribution) shown in **b**, **c**, **d**; a.u. arbitrary units. **h** Fluorescence microscopy image of a 2D array of HRP-containing differentiated protocells comprising a spatially separated tetra-modal distribution of **PS**_**WV**_, **PS**_**CV**_, **P**_**CB**_ and **P**_**CV**_ (from left to right, see graphics) morphological forms. The image is recorded 300 s after injection of a mixture of Amplex red and H_2_O_2_ across the 2D array; scale bar, 500 µm. **i** Average fluorescence line intensity profiles recorded across the tetra-modal population distribution shown in **h** at 0 (black line), 120 (blue) and 300 s (red) after addition of Amplex red and H_2_O_2_. **j** Plots of time-dependent changes in resorufin fluorescence mean intensity associated with individual HRP-containing **PS**_**WV**_ and **PS**_**CV**_ (red), **P**_**CB**_ (light blue) and **P**_**CV**_ (dark blue) types showing different rates of enzyme activity across the differentiated community. **k** Plots of the initial rates of HRP-mediated conversion of Amplex red to resorufin for data in **j**. Data recorded over the first 200 s; rate of conversion displayed as arbitrary units per second. The tetra-modal populations were prepared in opposing morphogen gradients (SDS, left side), POM (right side) at an initial SDS : POM molar ratio of 2.3 (70/30 µL; 50 mM). Source data are provided as a Source Data file. Error bars represent the standard deviation of the substrate conversion (*n* = 5)

Given the above observations, we explored whether the spatial and morphological differentiation produced in the presence of opposing SDS/POM morphogen gradients could be correlated with functional diversity across the organized consortia of membrane-bounded synthetic vesicles. For this, we chose the spatially separated tetra-modal population of POM/SDS hybrid wrinkled and non-wrinkled coacervate vesicles (**PS**_**WV**_ and **PS**_**CV**_) and single balloon-shaped and spherical POM coacervate vesicles (**P**_**CB**_ and **P**_**CV**_) prepared in counterflowing chemical gradients at an initial SDS : POM molar ratio of 2.3 and investigated the membrane permeability and enzyme activity of the differentiated protocells across the acoustically formed array. In the former case, we exposed the arrays of differentiated coacervate droplets to a series of organic dyes and used optical and fluorescence microscopy to assess the extent of molecular uptake in different vesicle types. Optical microscopy images showed no changes in the structure and spatial organization of the differentiated protocells before and after addition of the dye molecules (Fig. 5a). However, the corresponding fluorescence microscopy images indicated different levels of dye uptake in different populations of the differentiated coacervate droplets (Fig. 5b–g). For example, highly negatively charged molecules of pyranine were sequestered only within the **P**_**CV**_ population, whilst positively charged methylene blue was associated with both the **P**_**CB**_ and **P**_**CV**_ populations. In contrast, neutrally charged molecules of Nile Red were distributed in all four domains with lower fluorescence intensities recorded in the **PS**_**WV**_ and **PS**_**CV**_ populations. The results indicated that vesicles produced with a hybrid POM/SDS-containing membrane exhibited low permeabilities to small charged molecules. We attributed these differences in membrane selectivity to compositional and structural differences in the POM/PDDA or POM/SDS/PDDA outer shells produced as a consequence of morphogen-mediated differentiation in the opposing chemical gradients.

Having established a diversity of membrane properties in the differentiated droplets, we reasoned that it should be possible to use morphogen gradients to spontaneously generate spatially organised model protocell communities capable of exhibiting different functional outputs under the same environmental stimulus. As proof-of-principle, we prepared an array of horseradish peroxidase (HRP)-containing PDDA/ATP coacervate micro-droplets, transformed the homogeneous population in a counterflowing gradient of POM and SDS (SDS : POM = 2.3) to a tetra-modal spatial distribution of **PS**_**WV**_**, PS**_**CV**_, **P**_**CB**_ and **P**_**CV**_ forms, removed the supernatant and added a mixture of H_2_O_2_ and Amplex red (final concentrations in the chamber, 2.5 and 10 µM, respectively; molecular weight (Amplex red), MW = 257) to ensure a homogeneous distribution of the enzyme substrates across the array of differentiated protocells. Control experiments indicated that minimal leakage of HRP occurred after differentiation of the coacervate micro-droplets (Supplementary Fig. 38). HRP-mediated peroxidation of Amplex red specifically within the vesicles was monitored by measuring changes in red fluorescence intensity associated with the localized formation of the product resorufin. Within 5 min of injecting H_2_O_2_ and Amplex red homogeneously across the array, populations of the **PS**_**WV**_**, PS**_**CV**_, **P**_**CB**_ and **P**_**CV**_ protocells showed different responses to the same environmental input (Fig. 5h, Supplementary Fig. 39 and Supplementary Movie 11). The spherical POM/PDDA/ATP coacervate vesicles exhibited the highest increases in fluorescence intensity followed by lower levels of increased fluorescence intensity in the population of elongated POM/PDDA/ATP coacervate vesicles. In contrast, regions of the array containing vesicles with hybrid SDS/POM/PDDA membranes (**PS**_**WV**_ and **PS**_**CV**_) remained dark in the fluorescence microscopy images, indicating that HRP-mediated peroxidation was kinetically inhibited in these protocells (Supplementary Fig. 40). This was consistent with average fluorescence line profiles recorded across the differentiated consortium at different time intervals (Fig. 5i), which revealed that the **P**_**CV**_ population was approximately twice as active as the **P**_**CB**_ population (Fig. 5j, k).

We attributed the onset of functional diversity in the graded protocell populations principally to differences in their membrane permeability with respect to the uptake of Amplex red. This was consistent with analogous experiments in which Amplex red was replaced with a lower molecular weight substrate (*ortho*-phenylenediamine, *o*-PD; MW = 108) that was readily sequestered by all members of the tetra-modal population. Under these conditions, a yellow/green fluorescence (2,3-diaminophenazine, 2,3-DAP) response was observed across the entire array of HRP-containing differentiated protocells within 5 min of adding the substrate (Supplementary Fig. 41). However, the production of fluorescent 2,3-DAP was reduced in the **PS**_**WV**_ population compared with the **PS**_**CV**_, **P**_**CB**_ and **P**_**CV**_ forms, suggesting that high levels of SDS in the shell of the differentiated protocells was also responsible for lower levels of peroxidation. Other factors such as local differences in entrapped enzyme concentrations and diffusive transfer of resorufin across the array were not likely as the coacervate droplets were prepared with the same HRP concentration and membrane transfer of HRP and resorufin was observed to be negligible (Supplementary Figs. 38 and 42).

## Discussion

Taken together, our results demonstrate that spatially organized consortia of synthetic vesicles with structural and functional diversity can be spontaneously generated by exposing uniform arrays consisting of thousands of initially identical coacervate micro-droplets to unidirectional or counter-directional chemical gradients. Morphological differentiation of the membrane-free droplets into a range of membrane-bounded vesicles occurs under non-equilibrium conditions and is a spatiotemporal response to the advancing and intersecting artificial morphogen gradients. Tuning the gradients leads to modifications in the distribution of the reconfigured protocell populations such that delineated or intermingled consortia of functionally adapted vesicles can be spontaneously produced.

In general, the interaction and interplay of reactive flow systems with organized protocell arrays provide an approach to integrating non-equilibrium processes into the design of structurally and functionally graded populations of diverse cell-like entities. As a consequence, complex spatial and time-dependent behaviours of synthetic protocell consortia should be possible by managing the reaction fields accordingly through judicious control of the concentration gradients and their directionality. In the longer term, such systems could find applications in the development of chemically based microscale communication networks responsive to fluctuating inputs, artificial life platforms capable of environmental monitoring under non-equilibrium (flow) conditions, and population-informed sensing devices for detecting dynamical changes in metabolites.

Finally, as coacervate micro-droplets have been proposed as plausible progenitors to membrane-bounded protocells on the early Earth^51^, the ability of these molecularly crowded micro-compartments to artificially differentiate into communities of membrane-delineated vesicles in response to advancing chemical gradients could have implications for contemporary theories of the origin of life. In particular, we speculate that the origin of functional heterogeneity required for the emergence of competing proto-living systems in the early oceans could have derived from the non-equilibrium spatiotemporal transformation of homogeneous populations of immobilized coacervate droplets. Such droplets could be produced recursively in large numbers over extended time periods and exposed to geochemical reaction-diffusion gradients to produce co-existent and spatially distributed multimodal communities of differentiated protocells derived from the same chemical origin. More generally, we hypothesize that morphogen gradients on the early Earth could have provided a plethora of non-equilibrium pathways to the orchestration of physical and chemical morphogenesis, emergence of population dynamics in protocell landscapes and generation of abiogenic energy gradients.

## Methods

### Acoustic trapping of coacervate-based protocells

2D regular arrays of coacervate micro-droplets were prepared in a custom-built acoustic trapping device with a square arrangement of four piezoelectric transducers (Noliac, NCE 51, L15 × W2 × T1 mm). The opposing transducer pairs were wired in parallel, driven with sinusoidal waveforms by two signal generators (Agilent 33220a-001), and each connected to an oscilloscope (Agilent DSOX2014A). A PEGylated glass coverslip was attached with adhesive to the bottom of the device to serve as the base of a liquid sample trapping chamber (20 × 20 mm; total volume = 1 mL). Neutrally charged PDDA/ATP coacervate micro-droplets were prepared in situ by adding an aqueous solution of ATP (100 μL, 50 mM, pH 7) to the centre of the chamber containing an aqueous solution of PDDA (1 mL, 5 mM monomer, 100–200 kDa, pH 7) in the presence of two orthogonal acoustic standing waves generated from opposing transducer pairs operating at 6.76/6.78 MHz (10 V). The mixtures were stirred by pipetting, and the acoustic pressure field applied for at least 30 min to produce a periodic 2D array of immobilized coacervate micro-droplets. Fluorescent PDDA/ATP droplets were prepared by doping the polymer/nucleotide mixtures with a fluorescent derivative of ATP (TNP-ATP) or a rhodamine-tagged cationic polymer (RITC-PAH) with TNP-ATP: ATP or PAH: PDDA monomer molar ratios of 1 : 1000, or 1 : 9, respectively.

### Morphological transformations under equilibrium conditions

A 2D periodic array of immobilized membrane-free PDDA/ATP coacervate micro-droplets was prepared as described above. The supernatant was carefully removed and exchanged with Milli-Q water three times under the same acoustic force field. The acoustic pressure field was then switched off and 500 µL of water was removed from the acoustic trapping chamber. Pre-mixed solutions (500 µL) of chemical morphogens (SDS, POM, or mixtures of SDS and POM) were then injected into the sample chamber with vigorously stirring to ensure homogeneous mixing within the protocell array. (The protocells were undisturbed by the stirring due to their strong adsorption onto the glass substrate). The final concentrations of POM or SDS in the pre-mixed solutions were varied from 1–4 mM or 4–40 mM, respectively. For mixtures of SDS and POM, the combined concentrations of the two morphogens in the pre-mixed solutions (500 µL) were kept constant (*C*_*SDS*_ + *C*_*POM*_ = 10 mM), and the SDS : POM molar ratios systematically changed (SDS : POM = 9.0 (injected concentrations, 9 and 1 mM, respectively), 2.3 (7/3 mM), 1.0 (5/5 mM), 0.43 (3/7 mM) and 0.11 (1/9 mM). Representative optical and fluorescence microscopy images were recorded in the central observation window, which contained a square grid of approximately 33 × 45 droplets. Plots of the different types of membrane-bounded vesicles formed in the central observation window at various times were determined by counting the total amount of each individual population. Typically, 1500 protocells were counted for the statistical analysis.

### Protocell differentiation in morphogen gradients

A 2D periodic array of immobilized membrane-free PDDA/ATP coacervate micro-droplets was prepared as described above. After removal of the supernatant and washing, the acoustic pressure field was switched off and an aqueous solution of POM clusters (10–100 μL, 50 mM, pH 5) or SDS (10–100 μL, 50 mM) was injected using a pipette specifically from one edge of the trapping chamber (left side as viewed in the Figures) to generate a unidirectional gradient of a single morphogen within the pre-organized array of homogeneous PDDA/ATP coacervate micro-droplets. The morphogens were injected at the base of the device at a slow and constant rate to minimise turbulence at the diffusion front. Typically an induction time of 2–5 min was required for the morphogens to diffuse into the viewing window.

Alternatively, SDS and POM were injected almost simultaneously into the sample chamber from opposite edges (left (SDS) and right (POM) sides as viewed in the Figures) to generate unidirectional counterflowing gradients of the two morphogens across the pre-organized micro-droplet array. In general, SDS was injected first, followed immediately by POM at the opposite edge of the device. The concentration of the SDS and POM stock solutions (50 mM), the total volume (*V*) and combined concentrations (*C*) of the two morphogens were kept constant (*V*_*SDS*_ + *V*_*POM*_ = 100 µL; *C*_*SDS*_ + *C*_*POM*_ = 50 mM). The SDS : POM molar ratios were systematically changed between 9.0 (injections of 90 and 10 µL of 50 mM solutions, respectively), 2.3 (70/30 µL), 1.0 (50/50 µL), 0.43 (30/70 µL) and 0.11 (10/90 µL).

Time-lapse optical microscopy images of the coacervate droplet arrays undergoing differentiation were recorded in the central observation window (4 × 5 mm; 33 × 45 droplets) positioned *ca*. 10 mm from the point of injection of the morphogens. Representative slices from these images were used to present the data. Except for the time-dependent images, optical micrographs of the differentiated arrays are presented after no further morphological changes were observed.

Spatiotemporal changes in the distribution and segregation of the different types of membrane-bound vesicles in the array and their corresponding population dynamics were determined by counting each individual population in the 2D array according to their spatial position. In general, stable spatial distributions of the differentiated protocells were obtained within 60 min of injection of the morphogens.

### Functional diversity in differentiated populations

Differences in membrane permeability associated with specific morphological types of differentiated protocells produced under a counter-flow of SDS and POM (molar ratio = 2.3; injections of 70 and 30 µL of 50 mM solutions, respectively) were investigated by injecting pyranine (negatively charged, 3 µL, 1 mM), methylene blue (positively charged 5 µL, 1 mM) or Nile Red (neutral, 3 µL, 1 mM) into an acoustic trapping chamber containing a 2D array of differentiated PDDA/ATP micro-droplets (**PS**_**WV**_**, PS**_**CV**_, **P**_**CB**_ and **P**_**CV**_). The organic dyes were added after the supernatant in the acoustic trapping chamber had been carefully removed and exchanged three times with Milli-Q water. Fluorescence microscopy images were recorded 15 min after addition of the dyes to the array, and measurements of the membrane fluorescence intensity associated with the different morphological types of protocell determined

The influence of morphogen-mediated changes on the enzymatic reactivity of the PDDA/ATP micro-droplets was investigated as follows. Acoustically formed arrays of enzyme-containing PDDA/ATP micro-droplets were prepared as described above (ATP, 100 μL, 50 mM, pH 7; PDDA, 1 mL, 5 mM monomer, 100–200 kDa) but with the addition of HRP (HRP, 0.01 mg mL^−1^) to the PDDA solution. The homogeneous array was transformed into a tetra-modal spatial distribution consisting of **PS**_**WV**_**, PS**_**CV**_, **P**_**CB**_ and **P**_**CV**_ types by injecting SDS and POM (SDS : POM = 2.3 (70/30 µL; 50 mM) at opposite edges of the sample chamber. After 1 h, the supernatant was carefully removed and exchanged three times with Milli-Q water. A mixture of H_2_O_2_ (final concentration in the chamber, 10 µM) and Amplex red (final concentration, 2.5 µM) was then added to the array of morphologically differentiated protocells with vigorously stirring to ensure homogeneous mixing. (The protocells were undisturbed by the stirring due to their strong adsorption onto the glass substrate). The array was then left unstirred for the duration of the enzyme reaction. Fluorescence microscopy was used to detect the HRP-mediated conversion of non-fluorescence Amplex red to fluorescence resorufin (λ_ex_ = 515 − 560 nm and λ_em_ = 580 nm).

## Supplementary information

Supplementary Information

Supplementary Movie 1

Supplementary Movie 2

Supplementary Movie 3

Supplementary Movie 4

Supplementary Movie 5

Supplementary Movie 6

Supplementary Movie 7

Supplementary Movie 8

Supplementary Movie 9

Supplementary Movie 10

Supplementary Movie 11

Description of Additional Supplementary Files

Source Data file

## Data Availability

The authors declare that all relevant data supporting the finding of this study are available within the paper and its supplementary information files. The source data underlying Fig. 1c, f and I, 2c and f-I, 3 h, I and k-n, 4a-j and l-o, 5e-g and i-k and Supplementary Figs. 6c, 8, 14b-d, 15c-d, 18a and b, 19b-e, 28e and f, 29c and d, 30b-e, 31f-j, 32g-l, 33-f-n, 34f-o, 35f-n, 36, 37 and 38c-l are provided as a Source Data file. Additional data are available from the corresponding author upon request.
