# Supplementary material for: Artificial morphogen-mediated differentiation in synthetic protocells
Source: Nat Commun. 2019 Jul 25;10:3321. doi: 10.1038/s41467-019-11316-4 (PMC6658542; doi:10.1038/s41467-019-11316-4)
Supplement: Supplementary file 1 — Supplementary Information [file 41467_2019_11316_MOESM1_ESM.pdf]

Supplementary Information

Artificial morphogen-mediated differentiation in synthetic protocells

Tian et al

Supplementary Figures

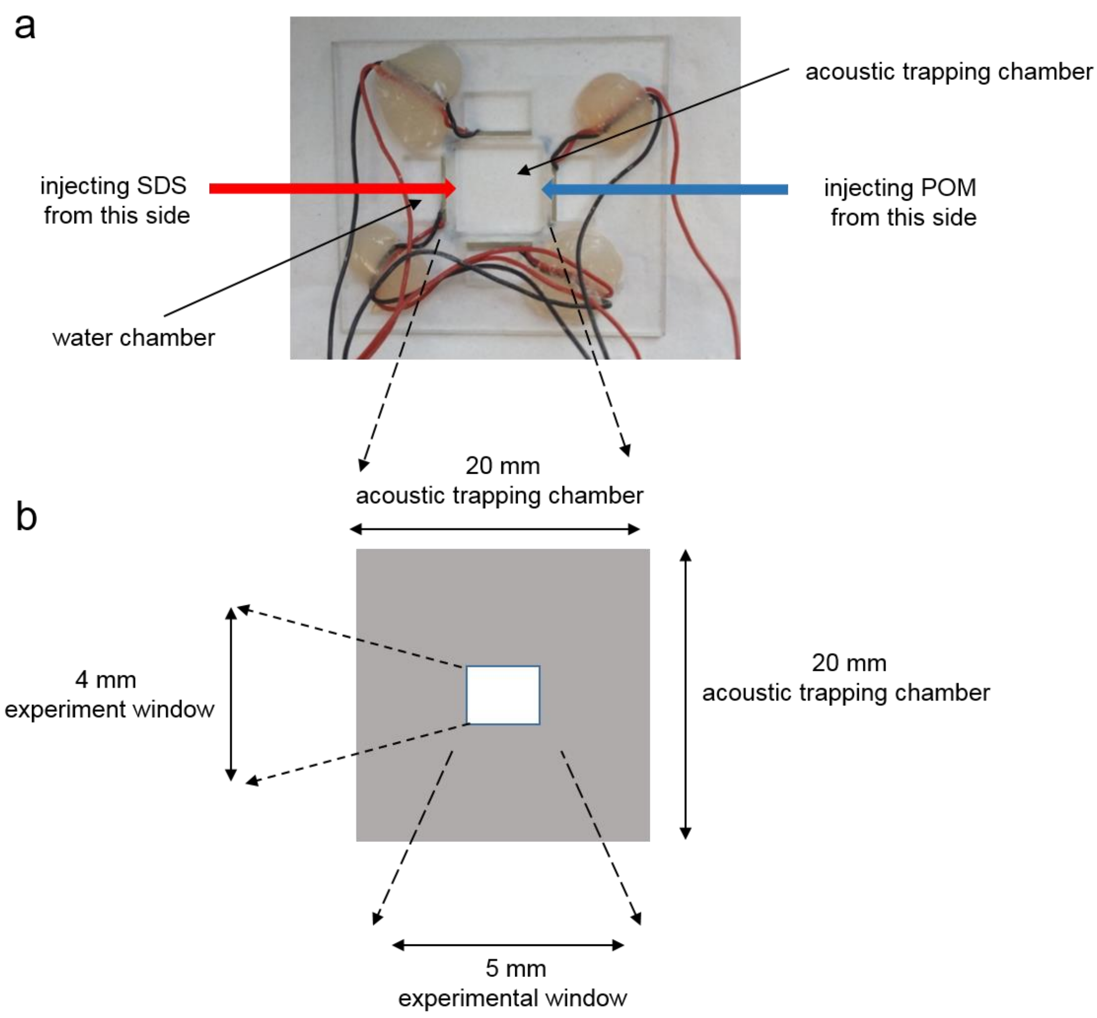

Supplementary Figure 1. (**a**) Optical image showing diffusion of SDS (left side) and/or POM clusters (sodium phosphotungstate; right side) into a 2D droplet array. Experiments involving unidirectional diffusion of POM or SDS, or counter-diffusion of POM and SDS were undertaken. The custom-made acoustic trapping device consists of a square acoustic trapping chamber (20 x 20 mm) with four rectangular water chambers to provide cooling. SDS or POM was injected specifically from one edge of the trapping chamber to generate a unidirectional gradient of a single morphogen within the pre-organized array of PDDA/ATP coacervate micro-droplets. Alternatively, SDS and POM were injected simultaneously at the left and right edges of the square acoustic trapping chamber, respectively, to generate opposing reaction-diffusion gradients of the morphogens. (**b**) Time-dependent observations were made in the central viewing window (4 x 5 mm) positioned *ca.* 10 mm from the point of morphogen injection. Typically, an induction time of *ca*. 5-10 min was required for a single morphogen to diffuse into the central viewing chamber.

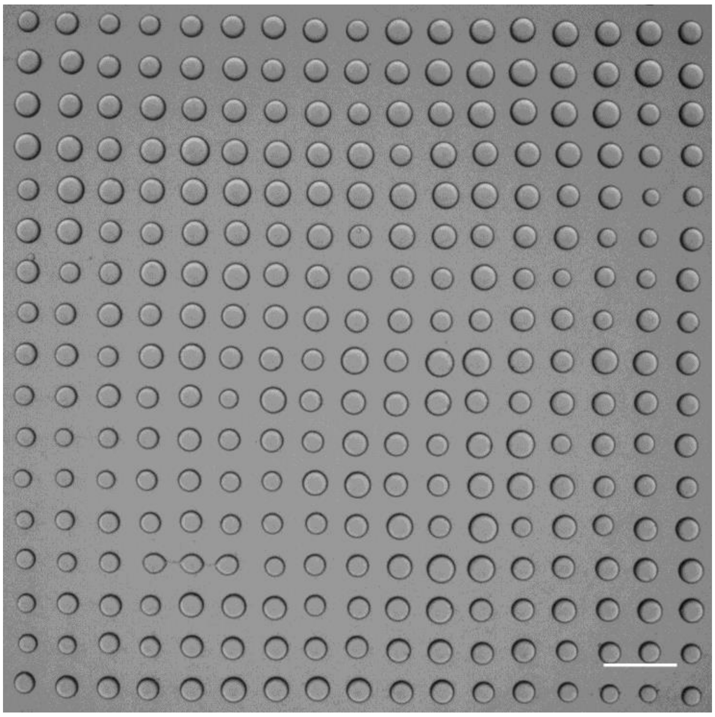

Supplementary Figure 2. Optical microscopy image showing a 2D array of PDDA/ATP coacervate micro-droplets prepared in the presence of two orthogonal acoustic standing waves generated from opposing transducer pairs operating at 6.76/6.78 MHz (10 V). Diameter of the droplets at each lattice point = 65 µm; lattice centre-to-centre spacing = 110 µm. The droplets are strongly attached to the underlying PEGylated glass substrate and remain in their lattice positions when the acoustic field is switched off. Scale bar = 200 µm.

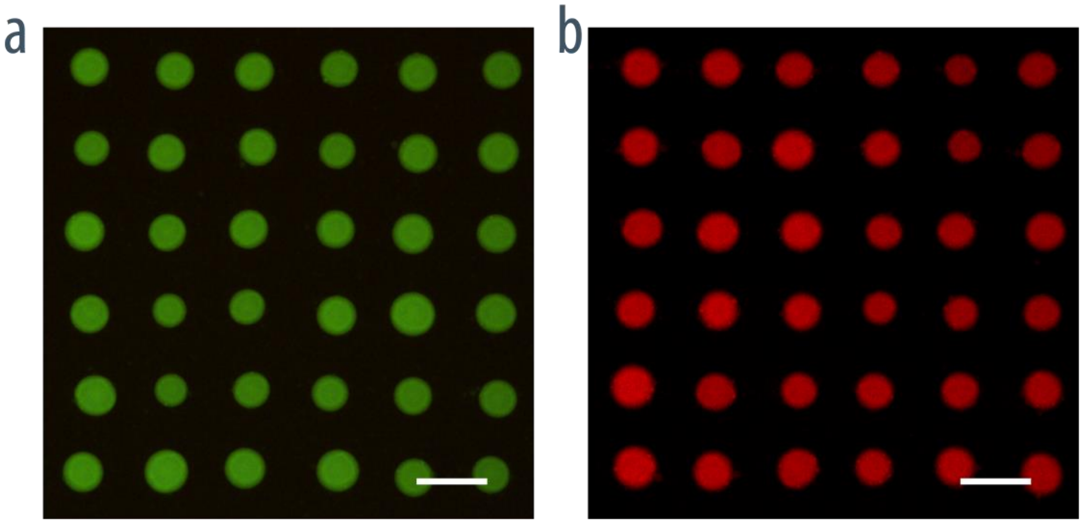

Supplementary Figure 3. (**a**,**b**) Fluorescence microscopy images of acoustically formed PDDA/ATP droplets doped with TNP-ATP (0.1 mol%)) (**a**) or rhodamine-labelled poly(allylamine hydrochloride) (RITC-PAH, 1 mol%)) (**b**). The droplet arrays were prepared in an acoustic trapping device operated under two orthogonal acoustic standing waves (6.76/6.78 MHz, 10 V). All scale bars = 100 µm.

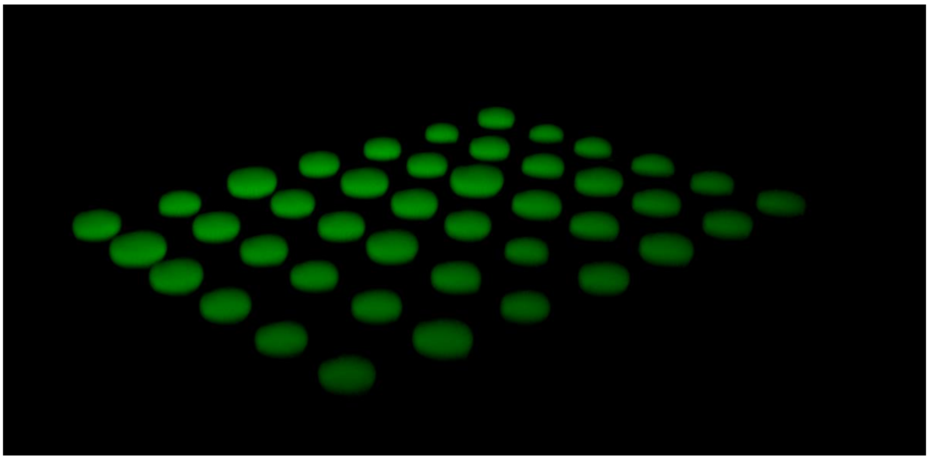

Supplementary Figure 4. 3D confocal fluorescence microscopy image of acoustically formed TNP-ATP-doped PDDA/ATP droplets showing strong attachment to the underlying PEGylated glass substrate of the sample chamber to produce a hemispherical morphology. The PDDA/ATP/TNP-ATP droplets were prepared by addition of a mixture of TNP-ATP (0.1 mol%) and ATP (100 µL, 50 mM) to a PDDA solution (1 mL, 5.0 mM monomer, 100-200 kDa) contained within the sample chamber of an acoustic trapping device operated under two orthogonal acoustic standing waves (6.76/6.78 MHz, 10 V).

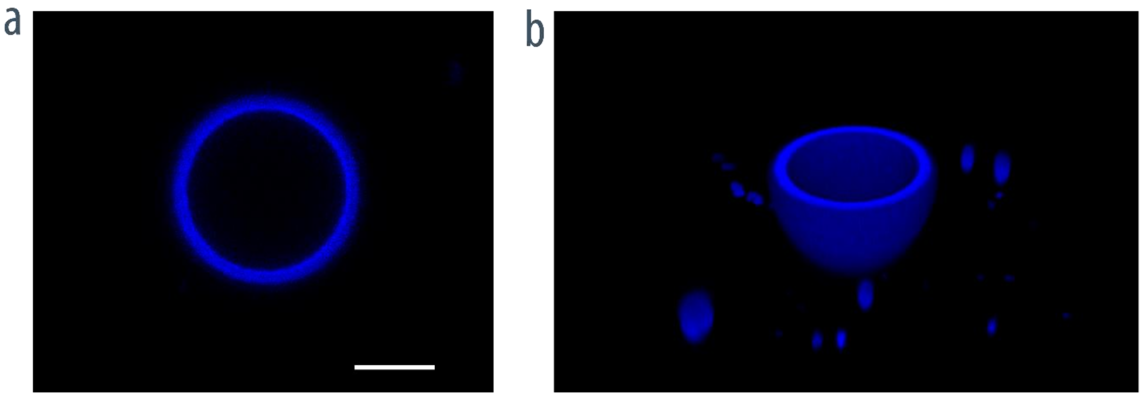

Supplementary Figure 5. (**a**,**b**) Confocal fluorescence microscopy (**a**) and 3D re-constructed image (Z-stack) (**b**) of a single POM/PDDA vesicle (**P$_{CV}$**); scale bar = 5 µm. The sample was prepared by addition of 500 µL of POM (sodium phosphotungstate, 4 mM) to a PDDA/ATP coacervate suspension (500 µL, 10 mM). Fluorescent PDDA/ATP droplets were prepared by doping the polymer/nucleotide coacervate with a DyLight 405-tagged cationic polymer (DyLight 405-PAH) with a PAH : PDDA monomer molar ratio of 1 : 100. The images show the presence of a *ca.* 1 µm-thick coacervate phase that is compressed against the outer POM/PDDA membrane by an expanded water lumen.

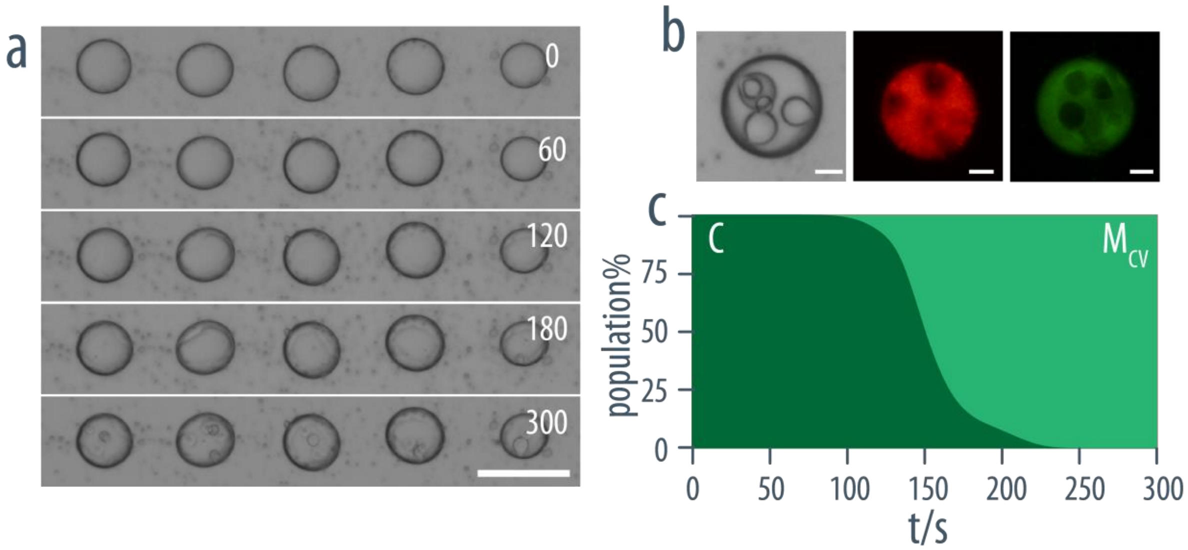

Supplementary Figure 6. (**a**) Time (*t*)-dependent optical microscopy images recorded from an acoustically formed array of PDDA/ATP coacervate micro-droplets at 0, 60, 120, 180 and 300 s after addition of a stirred solution of polyanionic POM clusters (sodium phosphotungstate final concentration = 0.5 mM; 500 µL, 1 mM injection) into the sample chamber. Images show 5 droplets in a single row of the array viewed in the central observation window. The membrane-free coacervate droplets transform into membrane-bounded coacervate vesicles containing multiple internalized water micro-domains (**M$_{CV}$**) (see images at 300 s). Scale bar = 100 µm. (**b**) Representative optical microscopy image (left), and RITC-PAH-doped (middle, red fluorescence) and TNP-ATP-doped (right, green fluorescence) fluorescence microscopy images of a single **M$_{CV}$**. The vesicles comprise an optically dense POM/PDDA thin outer membrane (thin dark ring), an optically transparent and fluorescent PDDA/ATP coacervate sub-membrane phase, and several low contrast aqueous lumens; scale bars = 20 µm. (**c**) Corresponding area plot showing time-dependent changes in the numbers of native coacervate micro-droplets (**C**, dark green) and multi-compartmentalized POM/coacervate vesicles (**M$_{CV}$**, light green) over 300 s. Changes in populations are shown as percentage of total. Source data are provided as a Source Data file.

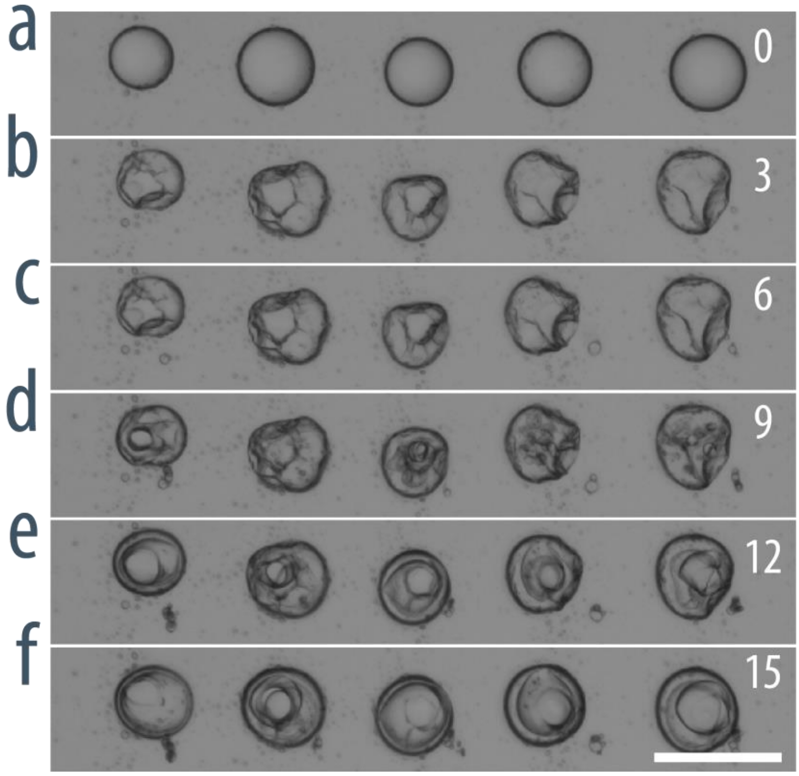

Supplementary Figure 7. (**a-f**) Time-dependent optical microscopy images recorded from an acoustically formed array of PDDA/ATP coacervate micro-droplets at 0, 3, 6, 9, 12 and 15 min after addition of a stirred solution of polyanionic POM clusters (sodium phosphotungstate; final concentration = 20 mM) into the sample chamber. Images show 5 droplets in a single row of the array viewed in the central observation window. The membrane-free coacervate droplets (**a**) immediately collapse on addition of the POM clusters (**b-c**) due to changes in osmotic pressure associated with the high POM concentration. As a consequence, transition of the droplets to spherical POM/coacervate vesicles ($\mathbf{P_{CV}}$) is kinetically inhibited. After 6 min, multiple internalized water micro-droplets ($\mathbf{M_{CV}}$) form in the collapsed droplets and induce re-swelling as the POM/PDDA membrane is established (**d-e**). The $\mathbf{M_{CV}}$ intermediates subsequently transform over a period of *ca.* 3 min into the $\mathbf{P_{CV}}$ morphological type (**f**). Scale bar = 100 µm.

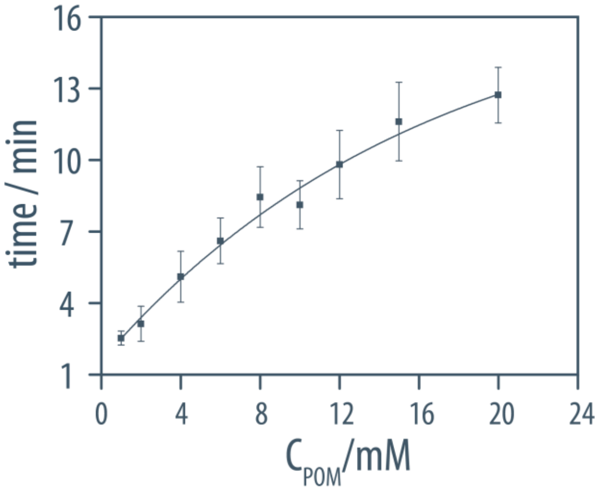

Supplementary Figure 8. Plot of the average time required for PDDA/ATP coacervate droplets to transform into POM/coacervate vesicles against the final concentration of POM under non-diffusive equilibrium conditions. The plot shows that increases in POM concentration kinetically inhibit the morphological transformation. Source data are provided as a Source Data file. Error bars represent the standard deviation of the statistics count of the different protocells (n = 88).

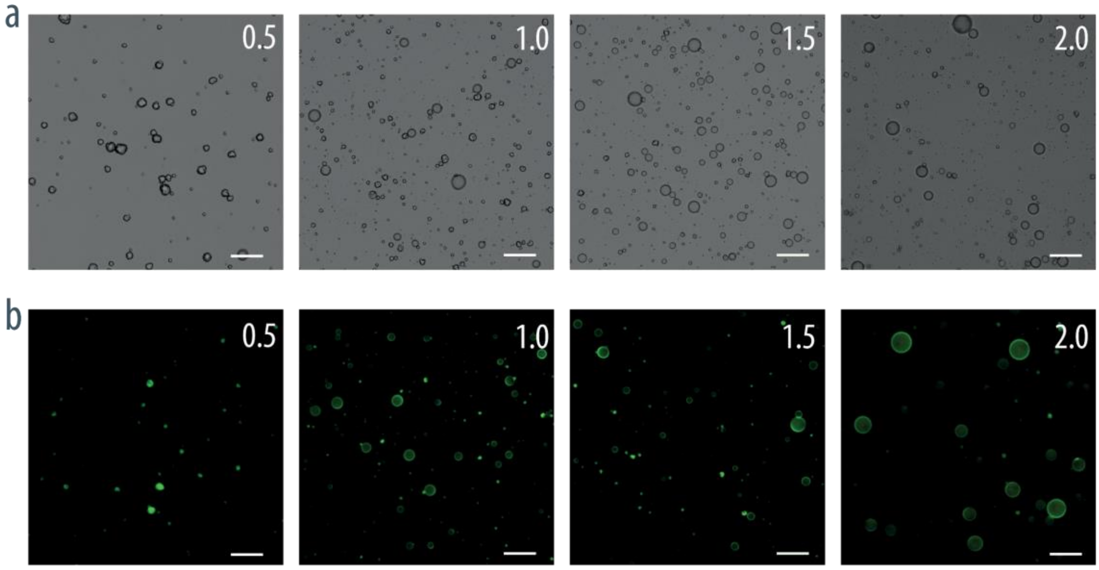

Supplementary Figure 9. Control experiments. (**a,b**) Optical (**a**) and fluorescence (**b**) microscopy images showing effect of adding an aqueous solution of POM clusters (from left to right, [POM] = 0.5, 1.0, 1.5 and 2 mM) to a re-dispersed suspension of PDDA/ATP coacervate micro-droplets (5 mM). Spherical POM/coacervate vesicles are produced at all POM concentrations. Fluorescent PDDA/ATP droplets were prepared by doping the polymer/nucleotide coacervate with a fluorescent derivative of ATP (TNP-ATP) at a TNP-ATP : ATP molar ratio of 1 : 1000. All scale bars = 50 µm.

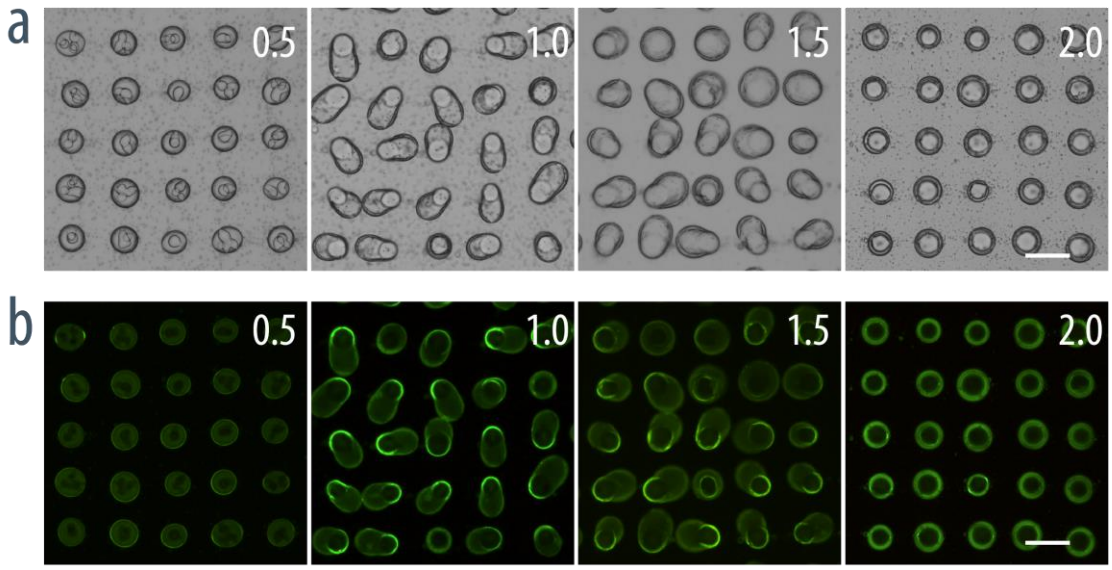

Supplementary Figure 10. (**a,b**) Optical (**a**) and fluorescence (**b**) microscopy images showing different morphological transformations associated with the addition of an aqueous solution of POM clusters (sodium polytungstate) to a PDDA/ATP coacervate micro-droplet array under non-diffusive equilibrium conditions. Images were recorded after 30 min. The membrane-free coacervate droplets are transformed *in situ* into membrane-bounded multi-compartmentalized coacervate vesicles (**M**$_{CV}$), balloon-shaped POM/coacervate vesicles (**P**$_{CB}$) or spherical POM/coacervate vesicles (**P**$_{CV}$) at POM final concentrations of 0.5, 1.0, 1.5 and 2.0 mM, respectively. The droplet array was prepared by addition of ATP (100 µL, 50 mM) to a PDDA (1 mL, 5 mM monomer, 100-200 kDa) solution contained within the sample chamber of an acoustic trapping device constructed with two transducer pairs operating at 6.76/6.78 MHz (10 V). Fluorescent PDDA/ATP droplets were prepared by doping the polymer/nucleotide coacervate with a fluorescent derivative of ATP (TNP-ATP) with a TNP-ATP : ATP molar ratio of 1 : 1000. All scale bars = 100 µm.

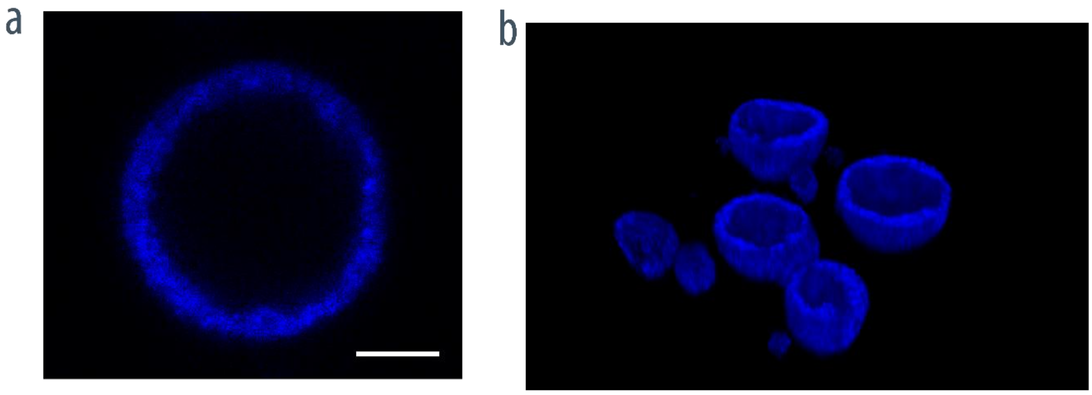

Supplementary Figure 11. (**a,b**) Confocal fluorescence microscopy (**a**) and 3D re-constructed image (**b**) of a single SDS/PDDA vesicle (**S**v); scale bar = 5 µm. The sample was prepared by adding 500 µL of SDS (40 mM) to a PDDA/ATP coacervate suspension (500 µL, 10 mM). Fluorescent PDDA/ATP droplets were prepared by doping the polymer/nucleotide mixtures with a DyLight 405-tagged cationic polymer (DyLight 405-PAH) at a PAH : PDDA monomer molar ratio of 1 : 100. The images show a polymer-enriched membrane surrounding an aqueous lumen.

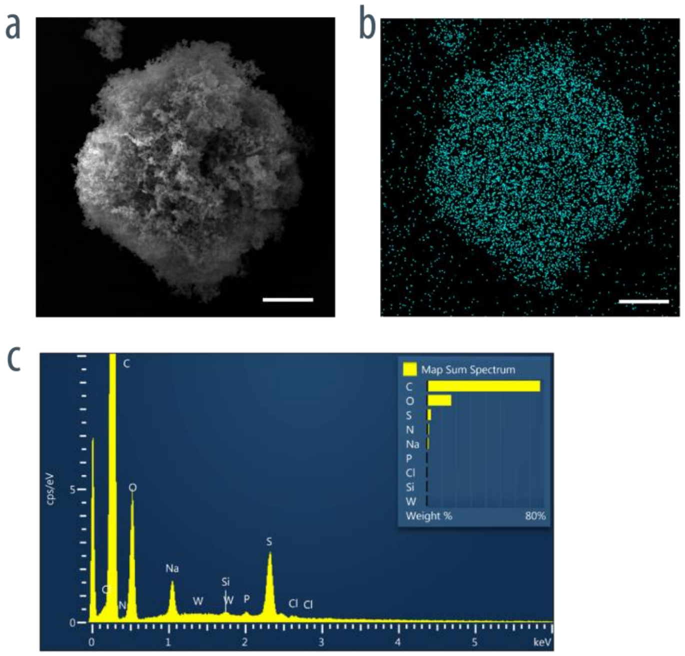

Supplementary Figure 12. (**a**) Representative scanning electron microscope (SEM) image of a single SDS/PDDA vesicle (**S**v). (**b-c**) Corresponding energy dispersive X ray (EDX) map of the sulphur distribution (**b**) and analysis profile (**c**) of the SDS/PDDA vesicle (**S**v) showing the presence of SDS in the membrane. Scale bar for (**a**) and(**b**), 20 µm. The sample was prepared by adding 500 µL of SDS (40 mM) to a PDDA/ATP coacervate suspension (500 µL, 10 mM). After 30 min, the sample was centrifuged three times at 5000 rpm for 5 min to remove the supernatant and re-dispersed in Milli-Q water. The suspension was then lyophilized, and the obtained powder coated with a 30 nm-thick coating of carbon for SEM imaging and EDX analysis. Lyophilization resulted in extensive damage to the soft surfactant/polymer shell as shown in (**a**).

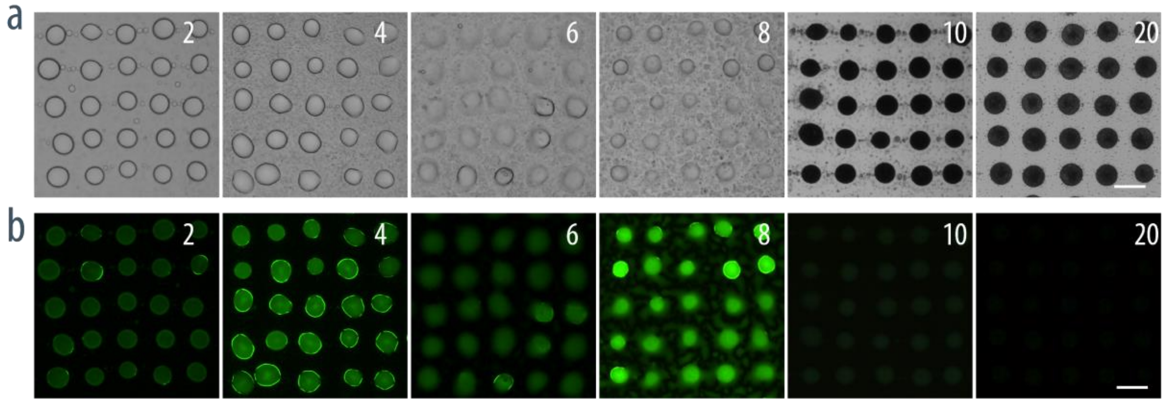

Supplementary Figure 13. (**a,b**) Optical (**a**) and fluorescence (**b**) microscopy images showing morphological transformations associated with the addition of aqueous solutions of SDS to a PDDA/ATP coacervate micro-droplet array under non-diffusive equilibrium conditions at final concentrations of 2, 4, 6, 8, 10 and 20 mM (from left to right). Images were recorded after 30 min. The PDDA/ATP micro-droplets array was prepared by adding ATP (100 µL, 50 mM) to a PDDA solution (1 mL, 5.0 mM monomer, 100-200 kDa) contained within the sample chamber of an acoustic trapping device operated under two orthogonal acoustic standing waves (6.76/6.78 MHz, 10 V). Fluorescent PDDA/ATP droplets were prepared by doping the polymer/nucleotide coacervate with a fluorescent derivative of ATP (TNP-ATP) with a TNP-ATP : ATP molar ratio of 1 : 1000. Minimal changes in morphology and composition are observed at concentrations below the critical SDS micelle concentration (<10 mM). In contrast, optically dark ATP-depleted SDS/PDDA vesicles (**S**$_V$) are observed in the presence of SDS micelles (10 and 20 mM). All scale bars = 100 µm.

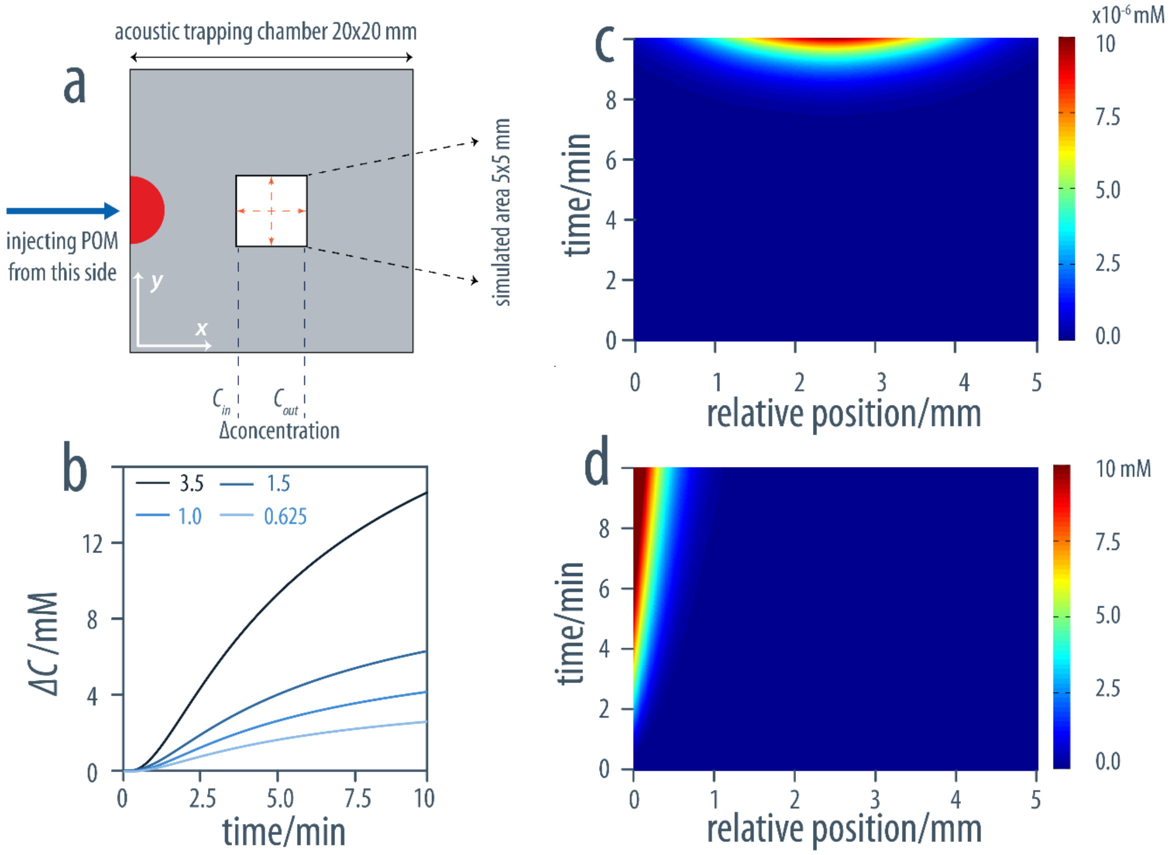

Supplementary Figure 14. (**a**) Simulation of a unidirectional POM morphogen gradient in the central observation window (white square; 5 x 5 mm) of the acoustic trapping device. POM is introduced into the device from the left side of the device along the *x* direction. Changes of POM concentration perpendicular (*y* axis) or parallel (*x* axis) to the diffusion wave front are indicated as the red arrows in the central square. The concentration gradient across the simulated area along the diffusion direction (*x* axis) is defined as: $\Delta C = C_{in} - C_{out}$. (**b**) Simulated time-dependent plots of the concentration gradients (ΔC) established across the observation window and along the diffusion direction (*x* axis) for various simulated POM injections (50 µL; 12.5, 20, 30 and 70 mM) delivered from the left-hand side of the device. The final simulated equilibrium POM concentrations are 0.625, 1, 1.5 and 3.5 mM in the acoustic trapping chamber, respectively. (**c**) Representative simulated 2D plots of the spatial and temporal distributions of POM concentration in a line of protocells aligned perpendicular to the diffusion of POM in the central viewing window of the acoustic trapping device showing an approximately planar diffusion front. (**d**) Representative simulated 2D plots of the spatial and temporal distributions of POM concentration in a line of protocells aligned parallel to the diffusion of POM in the central viewing window of the acoustic trapping device showing a propagating POM concentration gradient. The 2D simulations are for an injection of 50 µL of POM (70 mM; final concentration = 3.5 mM) solution along the *x* axis. The simulations are approximations as they did not consider binding of POM clusters at the coacervate surface (morphogen depletion) during the reaction-diffusion process. Source data are provided as a Source Data file.

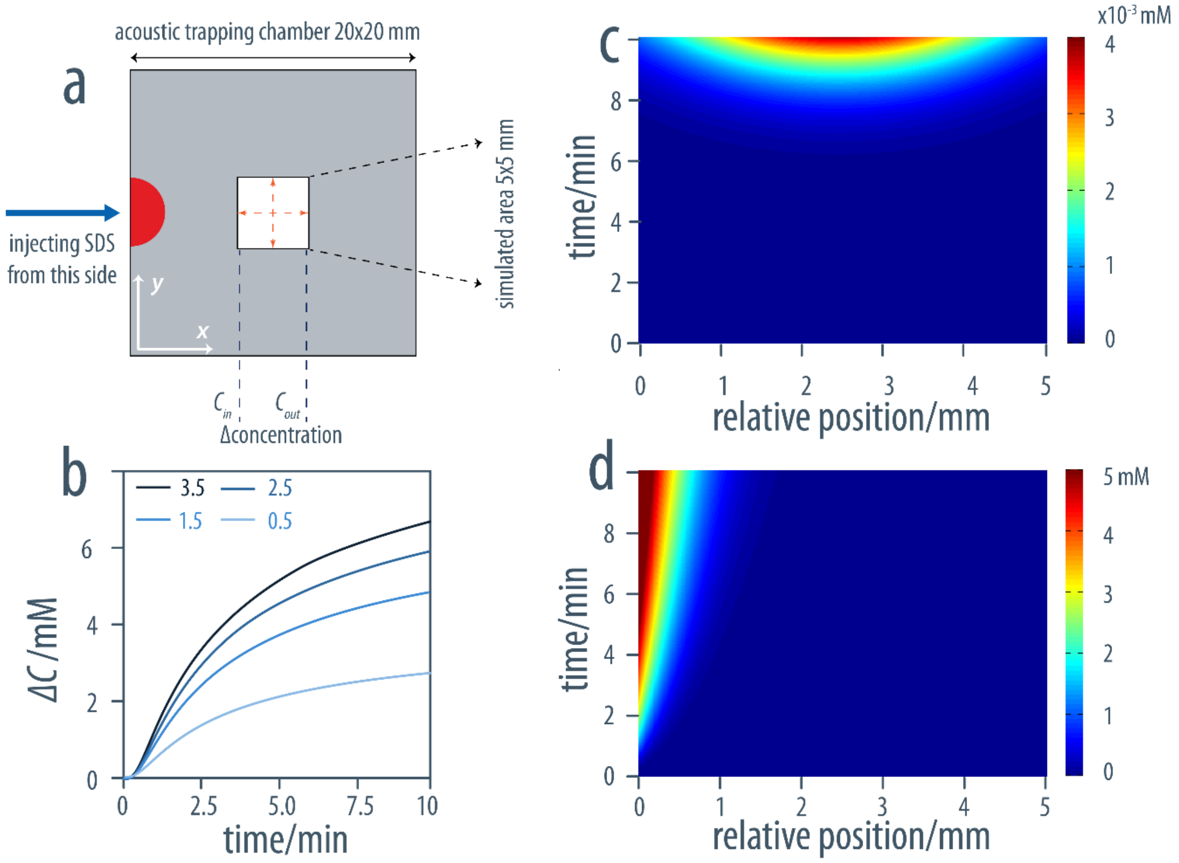

Supplementary Figure 15. (**a**) Schematic illustration of the simulated area in the acoustic trapping device. The simulated area is a 5x5 mm square in the centre of the device (observation window), and the changes of SDS concentration perpendicular or parallel to the diffusion wave front are indicated as the red arrows in the centre square. The concentration gradient across the simulated area along the diffusion direction (*x* axis) is defined as: $\Delta C = C_{in} - C_{out}$. (**b**) Simulated time-dependent plots of the concentration gradient (ΔC) along the diffusion direction (*x* axis) for various simulated molecular SDS injections (50 µL; 10, 30, 50 and 70 mM) delivered from the left-hand side of the device along the *x* direction. The final simulated equilibrium SDS concentrations are 0.5, 1. 5, 2.5 and 3.5 mM in the acoustic trapping chamber, respectively. (**c**) Representative simulated 2D plots of the spatial and temporal distributions of SDS concentration in a line of protocells aligned perpendicular to the diffusion of SDS in the central viewing window of the acoustic trapping device showing an approximately planar diffusion front. (**d**) Representative simulated 2D plots of the spatial and temporal distributions of SDS concentration in a line of protocells aligned parallel to the diffusion of SDS in the central viewing window of acoustic trapping device showing a propagating SDC concentration gradient. Simulations are for an injection of 50 µL of SDS (70 mM; final concentration = 3.5 mM) solution along the *x* axis. The simulations are approximations as they did not consider binding of SDS at the coacervate surface (morphogen depletion) during the reaction-diffusion process. Source data are provided as a Source Data file.

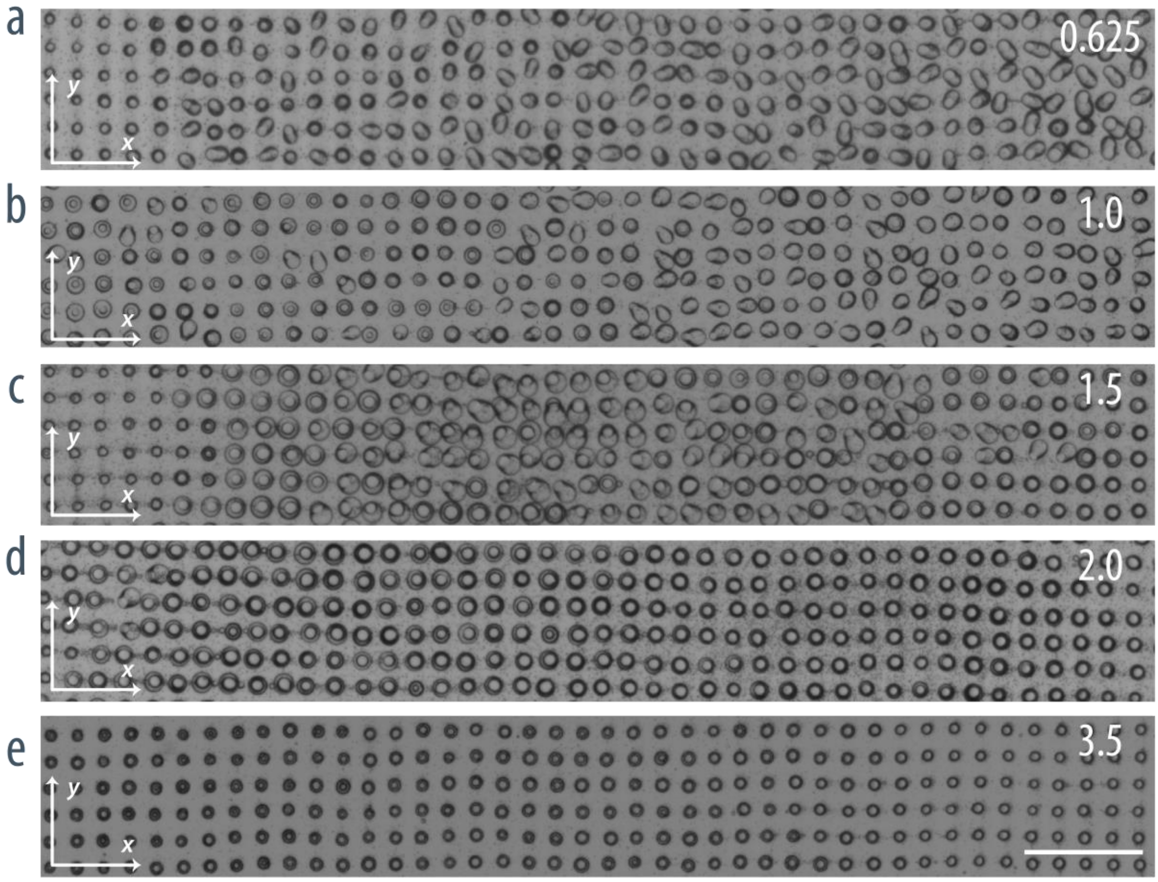

Supplementary Figure 16. (**a-e**) Optical microscopy images showing morphological differentiation in 2D arrays of PDDA/ATP coacervate droplets after exposure to various POM reaction-diffusion gradients. Only thin longitudinal sections of the protocell array in the observation window are displayed. The POM clusters are introduced into the chamber from the left-hand side of the image along the *x* direction. 50 µL of POM at a concentration of 12.5 (**a**), 20 (**b**), 30 (**c**), 40 (**d**) or 70 mM (**e**) were injected to give final equilibrium concentrations of 0.625, 1, 1.5, 2.0 or 3.5 mM, respectively. Images were recorded after no further changes in morphology were observed (15 min (**a,b**); 30 min (**c-e**). Binary populations of spherical POM/coacervate vesicles ($\mathbf{P_{CV}}$) (left side) and balloon-shaped POM/coacervate vesicles ($\mathbf{P_{CB}}$; right side) are observed between 0.625-1.5 mM (**b-d**). All the coacervate droplets were converted to $\mathbf{P_{CV}}$ when the final POM concentration was above 2.0 mM (**d,e**). Scale bar = 500 µm.

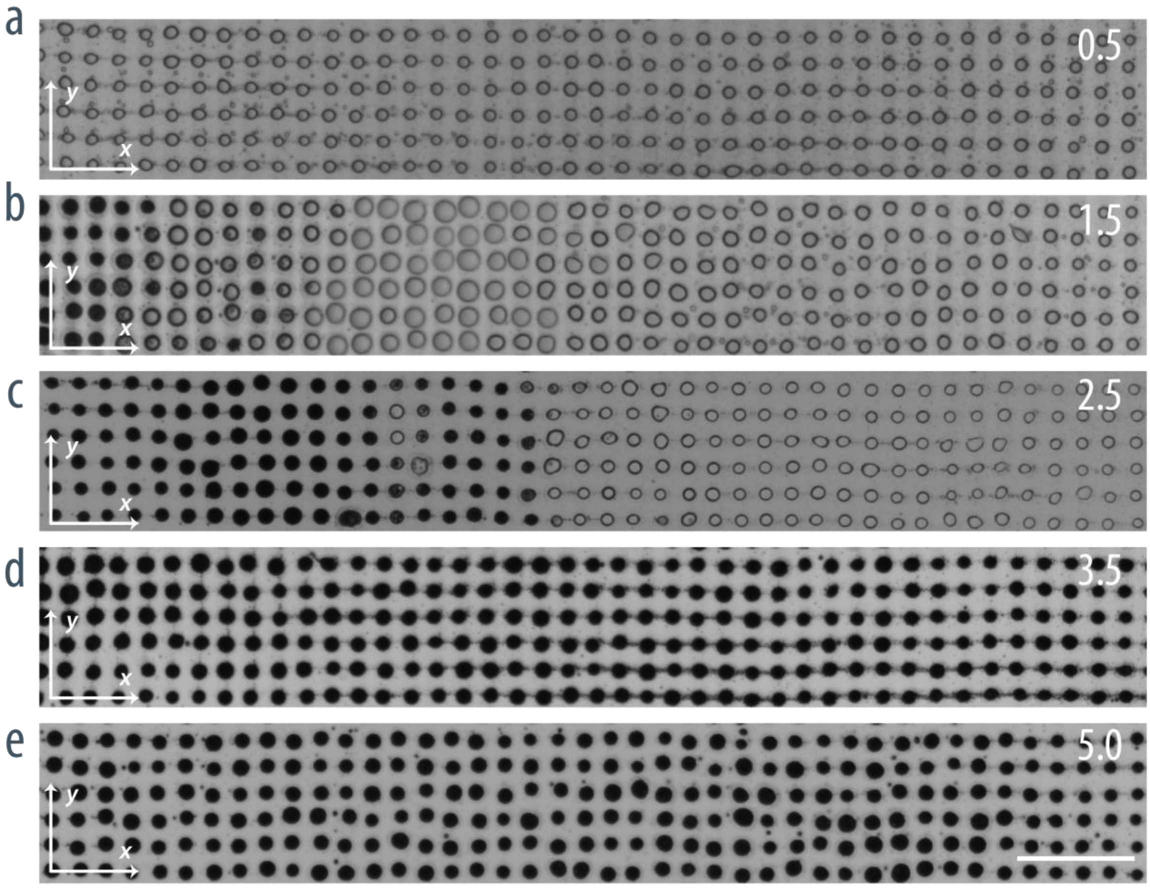

Supplementary Figure 17. (**a-e**) Optical microscopy images showing morphological differentiation in 2D arrays of PDDA/ATP coacervate droplets after exposure to various SDS reaction-diffusion gradients. Only thin longitudinal sections of the protocell array in the observation window are displayed. Images were recorded after no further changes in morphology were observed (60 min (**a**); 30 min (**b-e**). The SDS micelles clusters were introduced into the chamber from the left-hand side of the image along the *x* direction. The following volumes of a 50 mM SDS solution were injected; 10 (**a**), 30 (**b**), 50 (**c**), 70 (**d**) and 100 µL (**e**); final equilibrium concentrations: 0.5 (**a**), 1.5 (**b**), 2.5 (**c**), 3.5 (**d**) and 5 mM (**e**)). No changes in droplet morphology were observed in (**a**). In contrast, the homogeneous distribution of coacervate droplets differentiates into three spatially separated populations consisting of ATP-depleted SDS/PDDA vesicles (**S**$_V$; > 8 mM SDS; left side (**b**); left and centre (**c**)), multi-compartmentalized coacervate vesicles (**M**$_{CV}$; < 8 mM; centre-left (**b**); centre (**c**), and native coacervate droplets (**C**; < 4 mM; right (**b**); green filled circle; very low [SDS]) in (**b,c**). Enlarged droplets in the centre-left region of (**b**) arise from transient interfacial dewetting of the **M**$_{CV}$ types in the presence of SDS molecules. A single population of SDS/PDDA vesicles (**S**$_V$) is observed in (**d,e**). Scale bar = 500 µm.

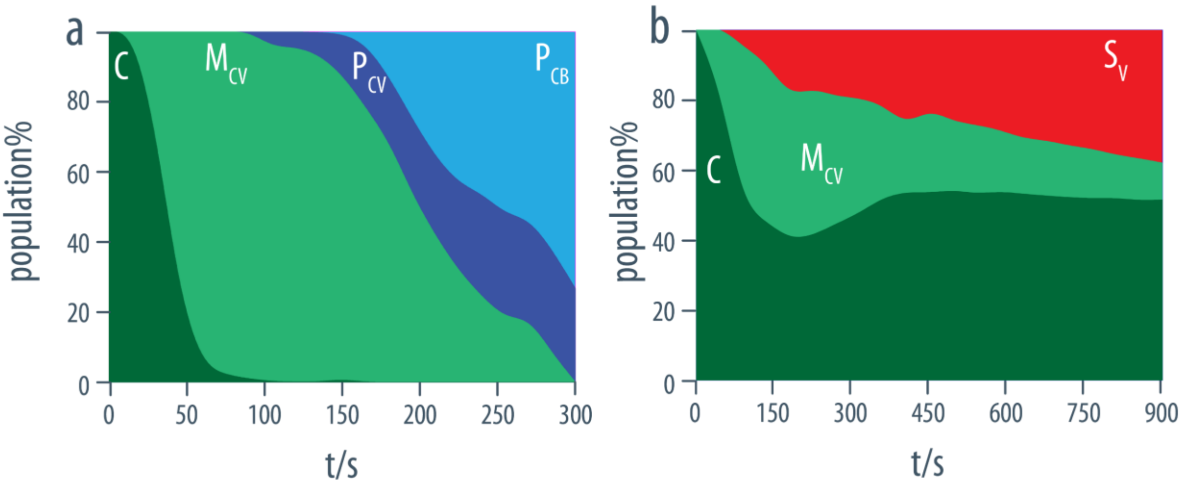

Supplementary Figure 18. (**a**) Area plot showing time-dependent changes in the numbers of native coacervate micro-droplets (**C**, dark green), multi-compartmentalized POM/coacervate vesicles (**M_{CV}**, light green), spherical coacervate single-compartment vesicles (**P_{CV}**) and balloon-shaped POM/coacervate vesicles (**P_{CB}**) over 300 s in the presence of a POM reaction-diffusion gradient (POM final equilibration concentration: 0.625 mM). (**b**) As for (**a**) but over 900 s in the presence of a SDS reaction-diffusion gradient (SDS final equilibration concentration: 2.5 mM). SDS/PDDA vesicles (**S_V**; red). Changes in populations are shown as percentage of total number of counted protocells (*n* = 1500). Source data are provided as a Source Data file.

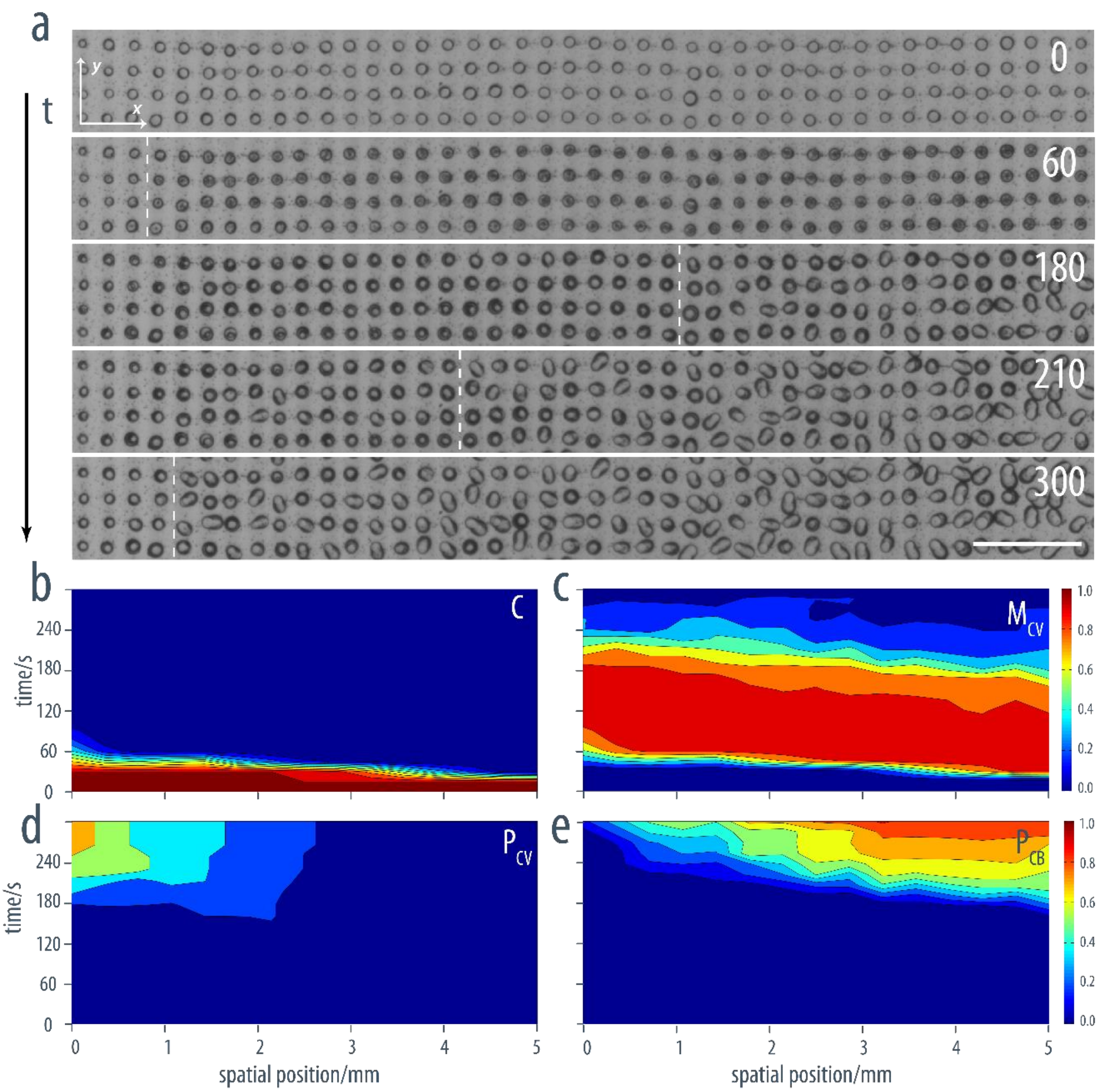

Supplementary Figure 19. (**a**) Time (*t*) dependent optical microscopy images recorded from an acoustically formed array of PDDA/ATP coacervate micro-droplets at 0, 60, 180, 210 and 300 s after addition of POM clusters into the sample chamber (50 µL, 12.5 mM; final POM concentration after equilibration, [POM] = 0.625 mM). POM is introduced into the chamber from the left-hand side of the image along the *x* direction. Only thin longitudinal sections of the protocell array in the observation window are displayed. Scale bar = 500 µm. (**b-e**) Corresponding 2D plots of the spatial and temporal distributions of the differentiated populations; (**b**) native coacervate (**C**); (**c**) multi-compartmentalized coacervate vesicle (**M$_{CV}$**); (**d**) spherical POM/coacervate vesicle (**P$_{CV}$**) and (**e**) balloon-shaped POM/coacervate vesicle (**P$_{CB}$**). Colour scale represents percentage of a given population. Total number of counted protocells, *n* = 1500. The **C** to **M$_{CV}$** transition occurs across the entire array after 60 s followed by subsequent transformation at around 180 s to populations of **P$_{CV}$** and **P$_{CB}$** in regions of higher [POM] (left side) and lower [POM] (right) in the reaction-diffusion gradient. All the coacervate droplets undergo morphological transformation. Formation of **P$_{CB}$** occurs initiates in areas of lower [POM] (right side) due to kinetic inhibition of the **M$_{CV}$** to **P$_{CB}$** transition at higher POM concentrations. Source data are provided as a Source Data file.

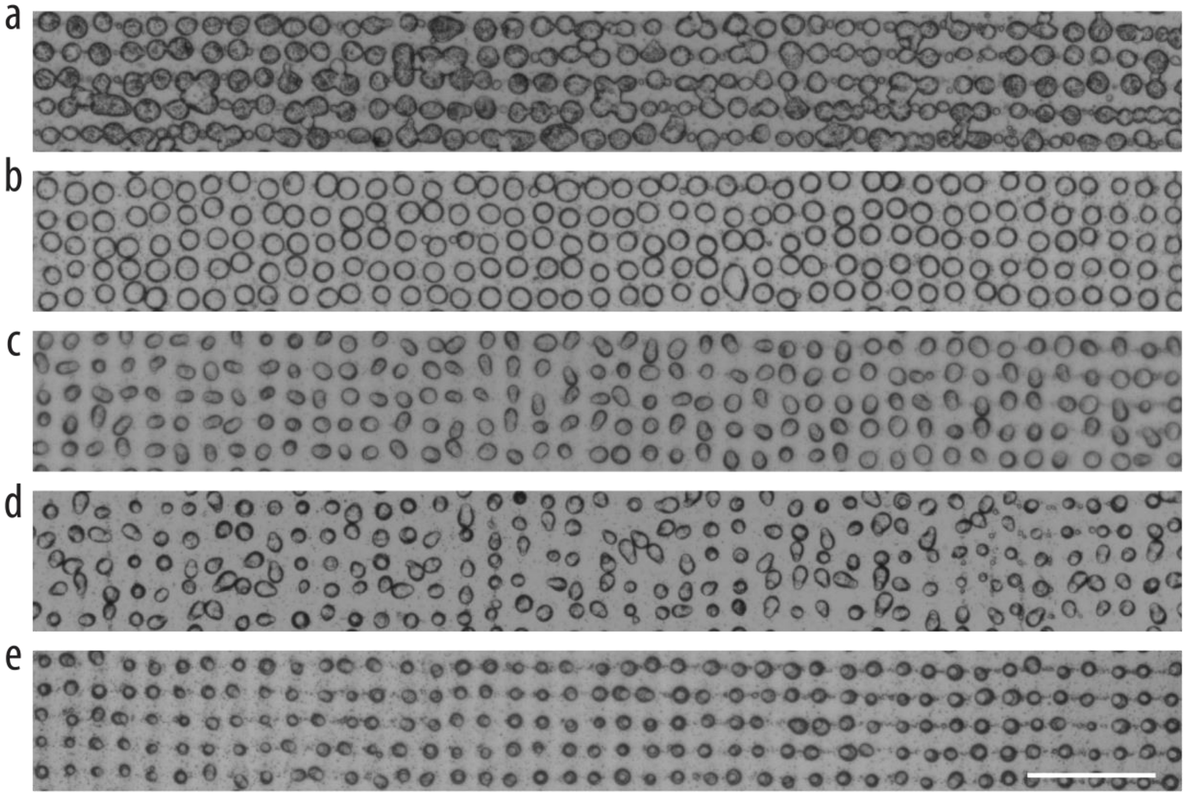

Supplementary Figure 20. (**a-e**) Optical microscopy images showing morphological transformations in 2D arrays of PDDA/ATP coacervate droplets after exposure to homogenous mixtures of SDS and POM (100 µL, C_{SDS} + C_{POM} = 50 µM) under non-diffusive equilibrium conditions at SDS : POM molar ratios of 9 : 1 (**a**), 7 : 3 (**b**), 5 : 5 (**c**), 3 : 7 (**d**) and 100% SDS (**e**). Only thin longitudinal sections of the protocell array in the observation window are displayed. Images were recorded after no further changes in morphology were observed (30 min). In each case, only a single population occurs after transformation; $\mathbf{PS_{WV}}$ (**a**), $\mathbf{PS_{CV}}$ (**b**), $\mathbf{P_{CB}}$ (**c-e**). Scale bar, 500 µm.

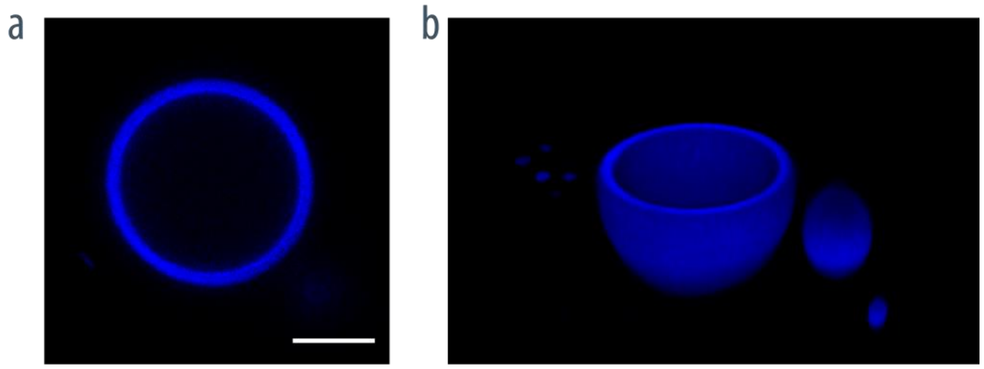

Supplementary Figure 21. (**a,b**) Confocal fluorescence microscopy (**a**) and 3D re-constructed image (**b**) of a single SDS/POM/PDDA coacervate vesicle (**PS$_{cv}$**); scale bar = 5 µm. The sample was prepared by adding 500 µL of POM (1 mM) and SDS (2 mM) to a PDDA/ATP coacervate suspension (500 µL, 10 mM). Fluorescent PDDA/ATP droplets were prepared by doping the polymer/nucleotide mixtures with a DyLight 405-tagged cationic polymer (DyLight 405-PAH) with a PAH : PDDA monomer molar ratio of 1 : 100. The images show a polymer-enriched coacervate shell surrounding an aqueous lumen.

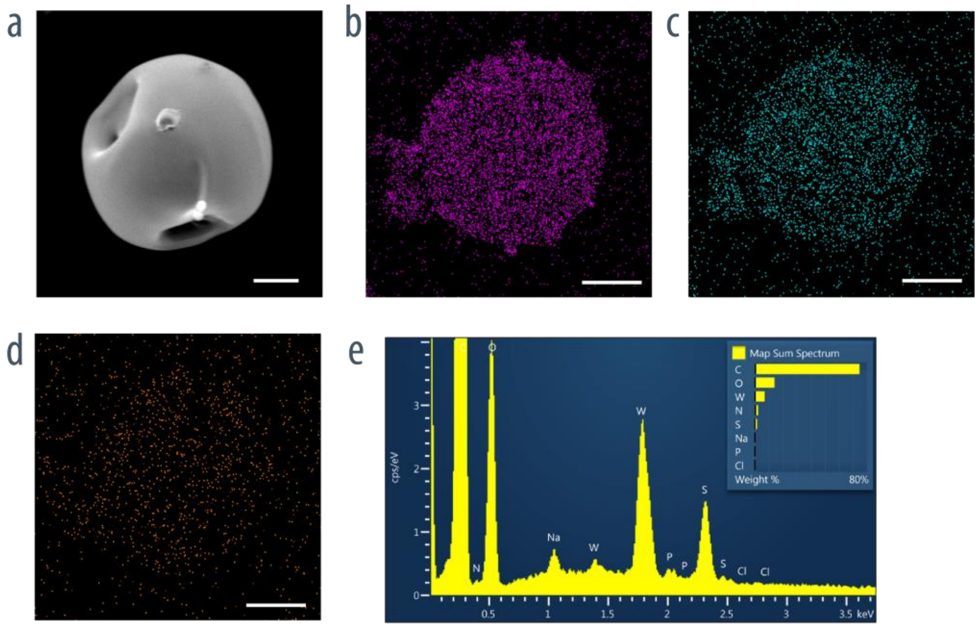

Supplementary Figure 22. (**a**) Representative scanning electron microscope (SEM) image of a single POM/SDS/PDDA coacervate vesicle (**PS$_{CV}$**). Note the intact membrane and compressed coacervate sub-membrane layer and hollow interior just visible through the perforated region towards the bottom of the vesicle. Scale bar, 5 µm. (**b-e**) Corresponding energy dispersive X ray (EDX) maps for tungsten (**b**), sulphur (**c**) and phosphorus (**d**), and analysis profile (**e**) showing co-location of POM (phosphotungstate) and SDS in the membrane. Scale bars for **b-d**, 10 µm. The sample was prepared by adding a mixture of SDS and POM solutions (500 µL, SDS/POM, 4/2 mM) to a PDDA/ATP coacervate suspension (500 µL, 10 mM). After 30 min, the sample was centrifuged three times at 5000 rpm for 5 min to remove the supernatant and re-dispersed in Milli-Q water. The suspension was then lyophilized, and the obtained powder coated with a 30 nm-thin coating of carbon for SEM imaging and EDX analysis.

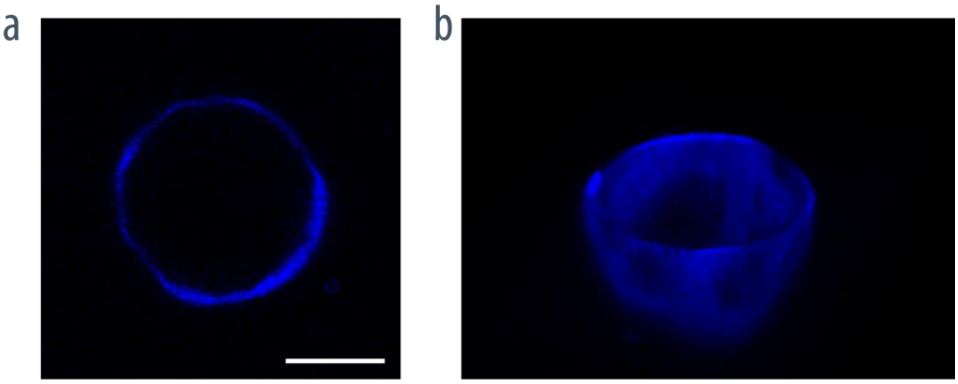

Supplementary Figure 23. (**a**,**b**) Confocal fluorescence microscopy (**a**) and 3D re-constructed image (**b**) of a single wrinkled SDS/POM/PDDA vesicle (**PS$_{wv}$**); scale bar = 20 µm. The sample was prepared by adding 500 µL of POM (4 mM) and SDS (20 mM) to a PDDA/ATP coacervate suspension (500 µL, 10 mM). Fluorescent PDDA/ATP droplets were prepared by doping the polymer/nucleotide mixtures with a DyLight 405-tagged cationic polymer (DyLight 405-PAH) with a PAH : PDDA monomer molar ratio of 1 : 100. The images show a polymer-enriched wrinkled membrane surrounding an aqueous lumen.

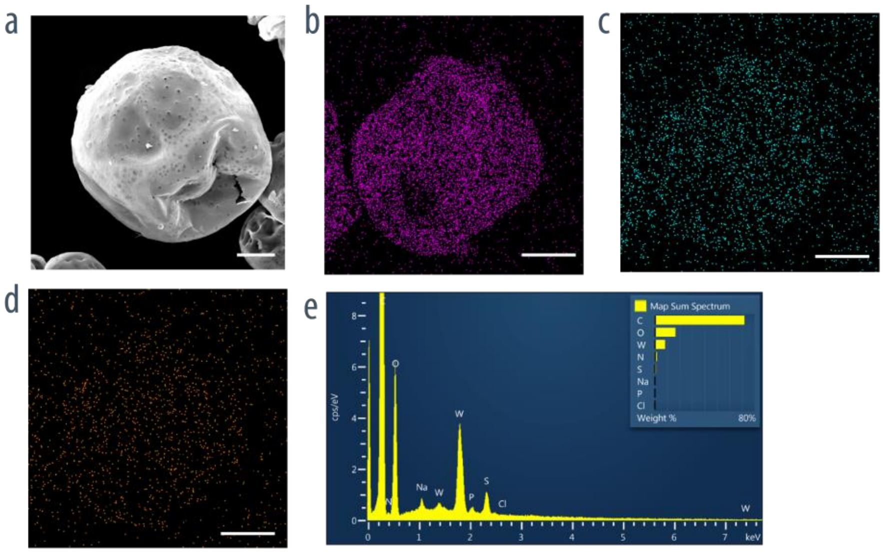

Supplementary Figure 24. (**a**) Representative scanning electron microscope (SEM) image of a single POM/SDS/PDDA wrinkled vesicle (**PS$_{wv}$**). Note the intact partially collapsed membrane, absence of a coacervate sub-membrane layer and hollow interior just visible through the perforated region towards the bottom of the vesicle. Scale bar, 10 µm. (**b-e**) Corresponding energy dispersive X ray (EDX) maps for tungsten (**b**), sulphur (**c**) and phosphorus (**d**) and analysis profile (**d**) showing co-location of POM (phosphotungstate) and SDS in the membrane. The low P count is consistent with the removal of ATP. Scale bars for **b** and **c**, 10 µm. The sample was prepared by adding a mixture of SDS and POM solution (500 µL, SDS/POM, 20/4 mM) to a PDDA/ATP coacervate suspension (500 µL, 10 mM). After 30 min, the sample was centrifuged three times at 5000 rpm for 5 min to remove the supernatant and re-dispersed in Milli-Q water. The suspension was then lyophilized, and the obtained powder was coated with 30 nm-thick coating of carbon for SEM imaging and EDX analysis.

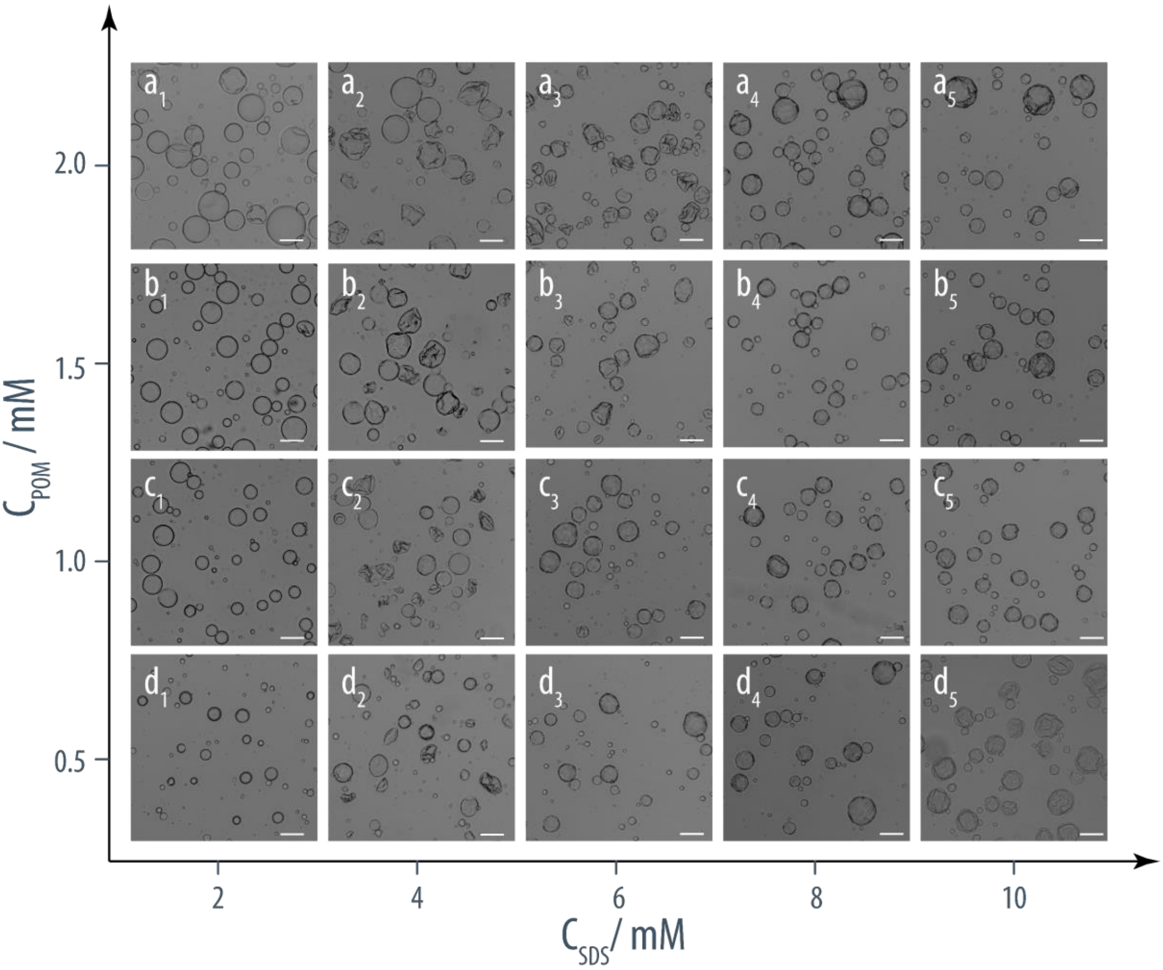

Supplementary Figure 25. (**a-d**) Optical microscopy images showing a range of morphological transformations after addition of a mixture of POM and SDS at various SDS/POM ratios to re-dispersed samples of PDDA/ATP coacervate micro-droplets (5 mM). Each row corresponds to a constant final POM concentration (C_{POM} = 2, 1.5, 1 or 0.5 mM in rows **a** to **d**, respectively). The final SDS concentration (C_{SDS}) in the mixture is 2, 4, 6, 8 or 10 mM (from left to right along each row). Large spherical hybrid capsules **PS$_{CV}$** are obtained when C_{SDS} and C_{POM} are below 3 and 1.75 mM, respectively. Mixtures of **PS$_{WV}$** and **PS$_{CV}$** or **PS$_{WV}$** alone were observed for a range of POM concentrations provided the respective final SDS concentrations were between 3 and 5 mM or above 5 mM. All scale bars = 50 µm.

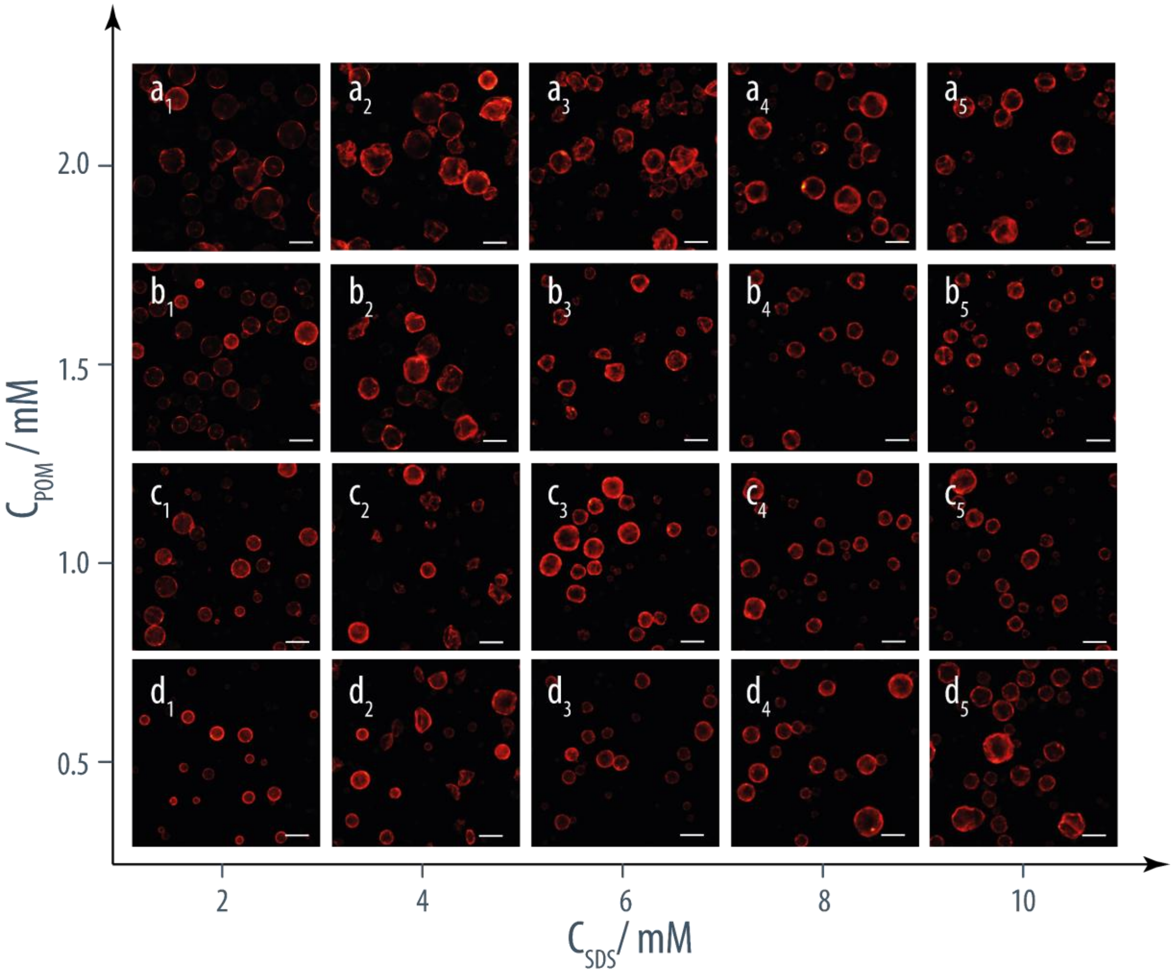

Supplementary Figure 26. (**a-d**) As for **Fig. S25** but showing fluorescence microscopy images. Rows **a** to **d;** C_{POM} = 2, 1.5, 1 or 0.5 mM, respectively; C_{SDS} = 2, 4, 6, 8 or 10 mM (from left to right along each row). Fluorescent PDDA/ATP coacervate micro-droplets (5 mM) were doped with a rhodamine-tagged cationic polymer (RITC-PAH; PAH : PDDA monomer molar ratio = 1 : 9). The fluorescence images confirmed the presence of the polycationic polymer in the hybrid protocell types (**PS**$_{WV}$ and **PS**$_{CV}$). All scale bars = 50 µm.

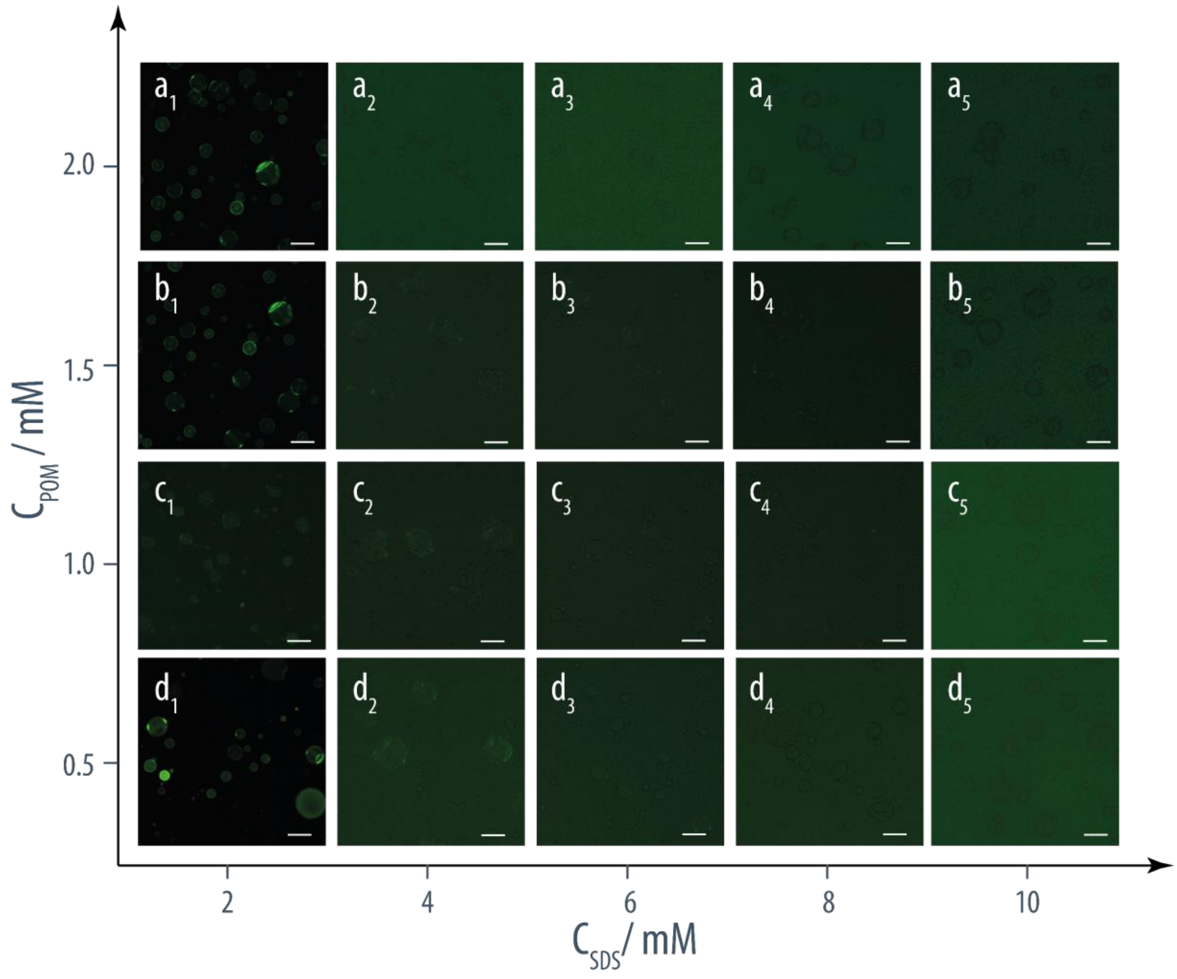

Supplementary Figure 27. (**a-d**) As for **Fig. S25** but showing fluorescence images. Rows **a** to **d**; C_{POM} = 2, 1.5, 1 or 0.5 mM, respectively; C_{SDS} = 2, 4, 6, 8 or 10 mM (from left to right along each row). Fluorescent PDDA/ATP coacervate micro-droplets (5 mM) were doped with TNP-ATP (TNP-ATP : ATP molar ratio = 1 : 1000). The fluorescence images confirmed that ATP is gradually excluded from the hybrid PS_{CV} protocells with increasing C_{SDS}. This effect was enhanced at constant C_{SDS} for increased values of C_{POM} in the morphogen mixtures. All scale bars = 50 µm.

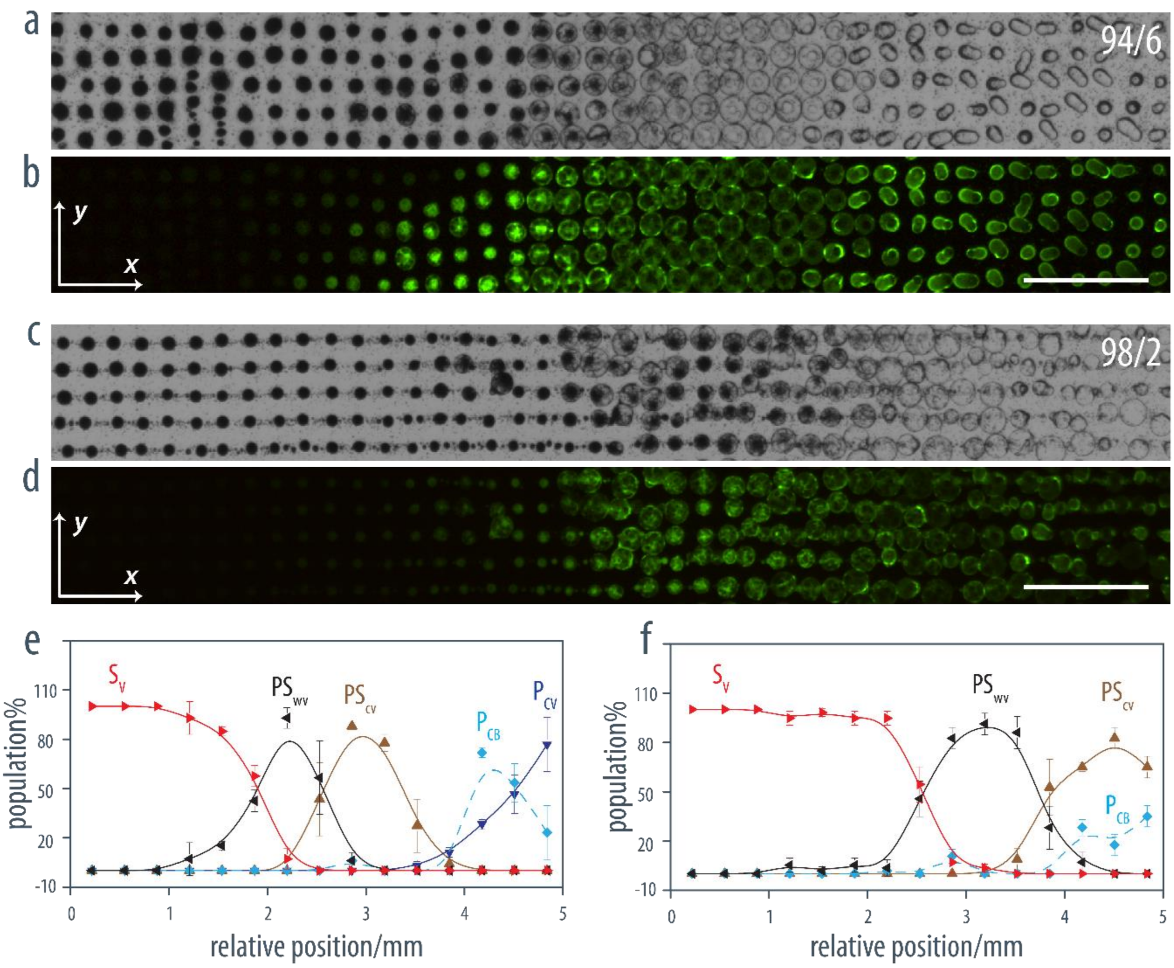

Supplementary Figure 28. (**a-d**) Optical (**a**,**c**) and corresponding fluorescence microscopy (**b**,**d**) showing 2D array of differentiated protocells viewed in the observation window after exposure to opposing reaction-diffusion gradients of SDS and POM generated from an initial SDS : POM morphogen ratio of 15.6 (94/6 µL; 50 mM) (**a-b**) and 49 (98/2 µL, 50 mM) (**c-d**). Green fluorescence originates from TNP-ATP and serves as a proxy for the presence of a coacervate phase. Scale bars, 500 µm. (**e-f**) Plots showing correlated changes in relative number densities of different types of differentiated protocells (% population) with spatial position along the diffusion direction (*x* axis) of opposing SDS and POM reaction-diffusion gradients for the data shown in (**a**) and (**c**). Five spatially distinct populations (**S_V**, **PS_{WV}**, **PS_{CV}**, **P_{CB}** and **P_{CV}**; from left to right) are observed in the intersection zone for (**a**), and four spatially distinct populations (**S_V**, **PS_{WV}**, **PS_{CV}** and **P_{CB}**; from left to right) are observed in the intersection zone for (**c**). Images were recorded after no further changes in morphology were observed (30 min). Displayed grid sizes, 5 x 42. Source data are provided as a Source Data file. Error bars represent the standard deviation of the statistics count of the different lines of different protocells (n = 3).

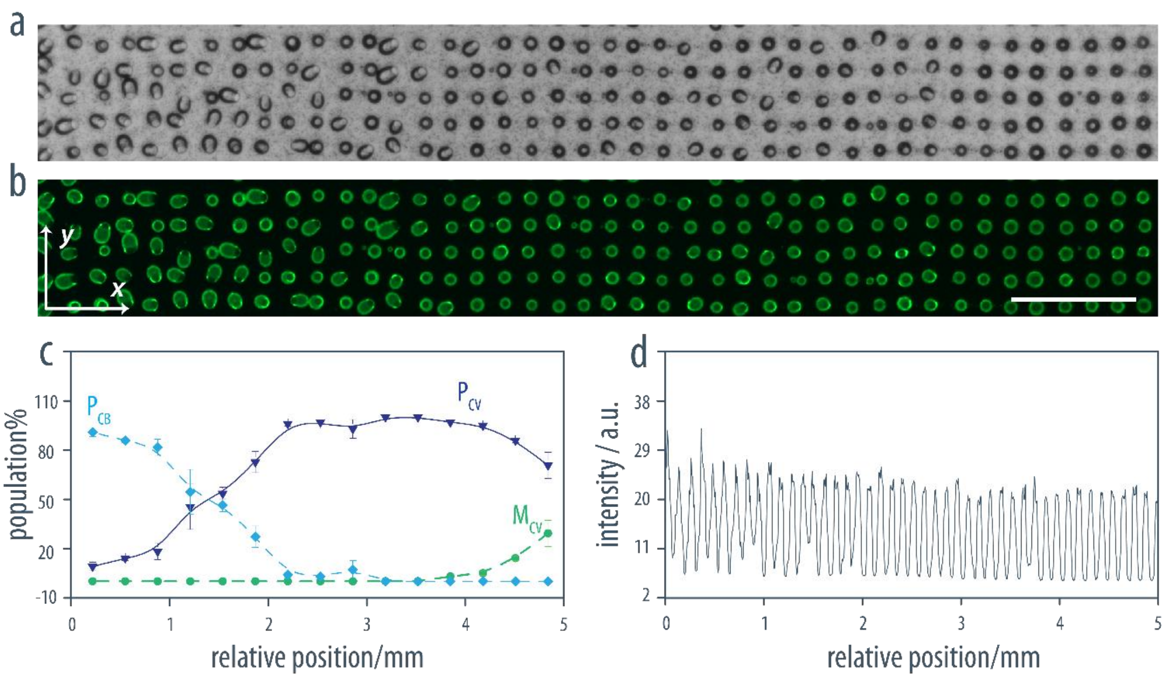

Supplementary Figure 29. (**a**,**b**) Optical (**a**) and corresponding fluorescence microscopy (**b**) showing 2D array of differentiated protocells viewed in the observation window after exposure to an opposing reaction-diffusion gradient of SDS and POM generated from an initial SDS : POM morphogen ratio of 0.11 (10/90 µL, 50 mM). Green fluorescence originates from TNP-ATP and serves as a proxy for the presence of a coacervate phase. Scale bar, 500 µm. (**c**) Plot showing correlated changes in relative number densities of different types of differentiated protocells (% population) with spatial position along the diffusion direction (*x* axis) of an opposing SDS and POM reaction-diffusion gradients for the data shown in **a**. Three spatially distinct populations (**P_{CB}, P_{CV}** and **M_{CV}**; from left to right) with two distinct boundaries are observed in the intersection zone. (**d**) Average fluorescence line intensity profile derived from (**b**). Source data are provided as a Source Data file. Error bars represent the standard deviation of the statistics count of the different lines of different protocells (n = 3).

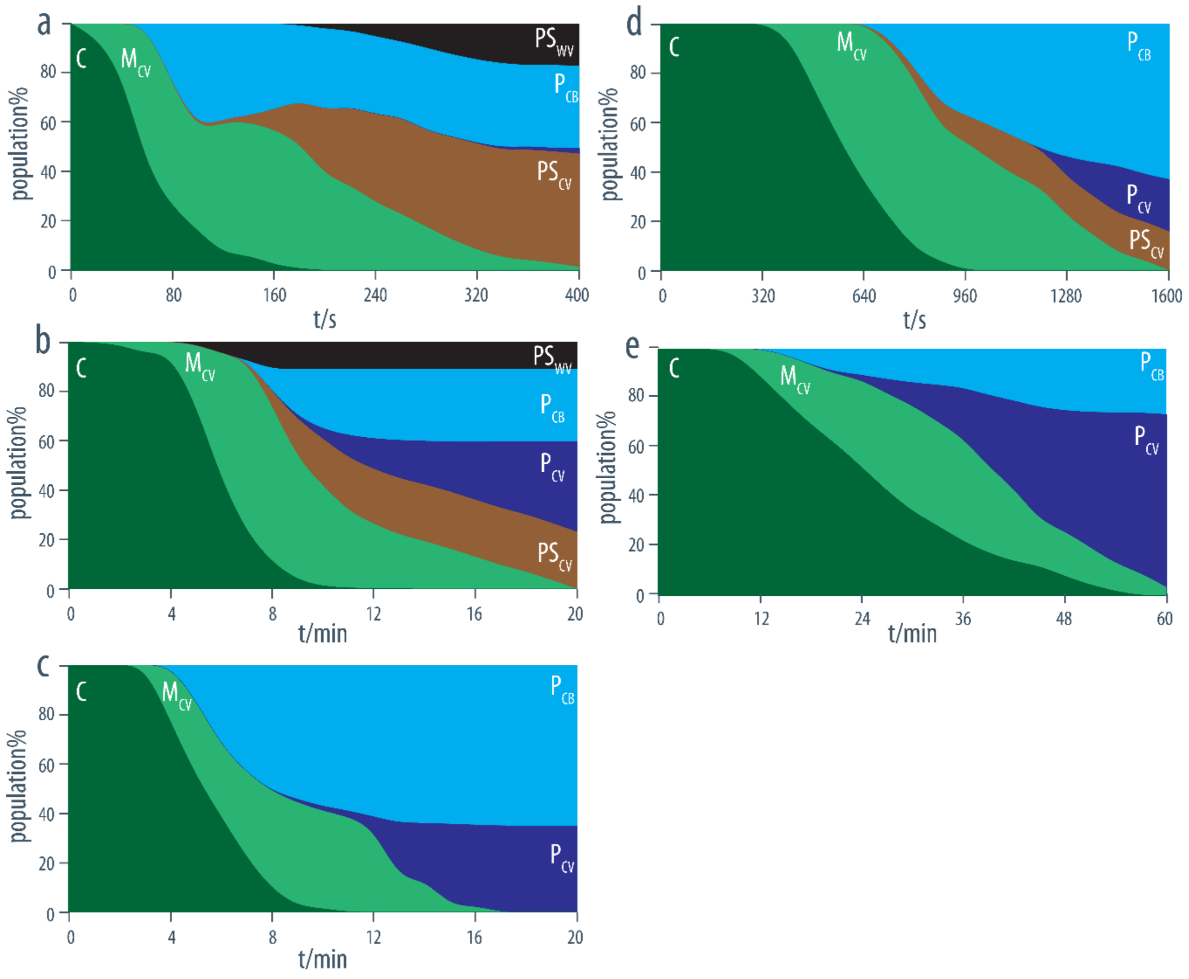

Supplementary Figure 30.(**a-e**) Area plots showing time-dependent changes in the numbers of native coacervate droplets (dark green, **C**) and differentiated protocells (light green, **M_{CV}**; blue **P_{CV}**; light blue **P_{CB}**; brown **PS_{CV}**; black, **PS_{WV}**) produced in opposing morphogen gradients prepared at initial SDS : POM molar ratios of 9.0 (90/10 µL; 50 mM) (**a**), 2.3 (70/30 µL, 50 mM) (**b**), 1.0 (50/50 µL; 50 mM) (**c**), 2.3 (70/30 µL; 50 mM) (**d**) and 0.11 (10/90 µL; 50 mM) (**e**). Changes in populations are shown as percentage of total; number of counted protocells, *n* = 1500. Source data are provided as a Source Data file.

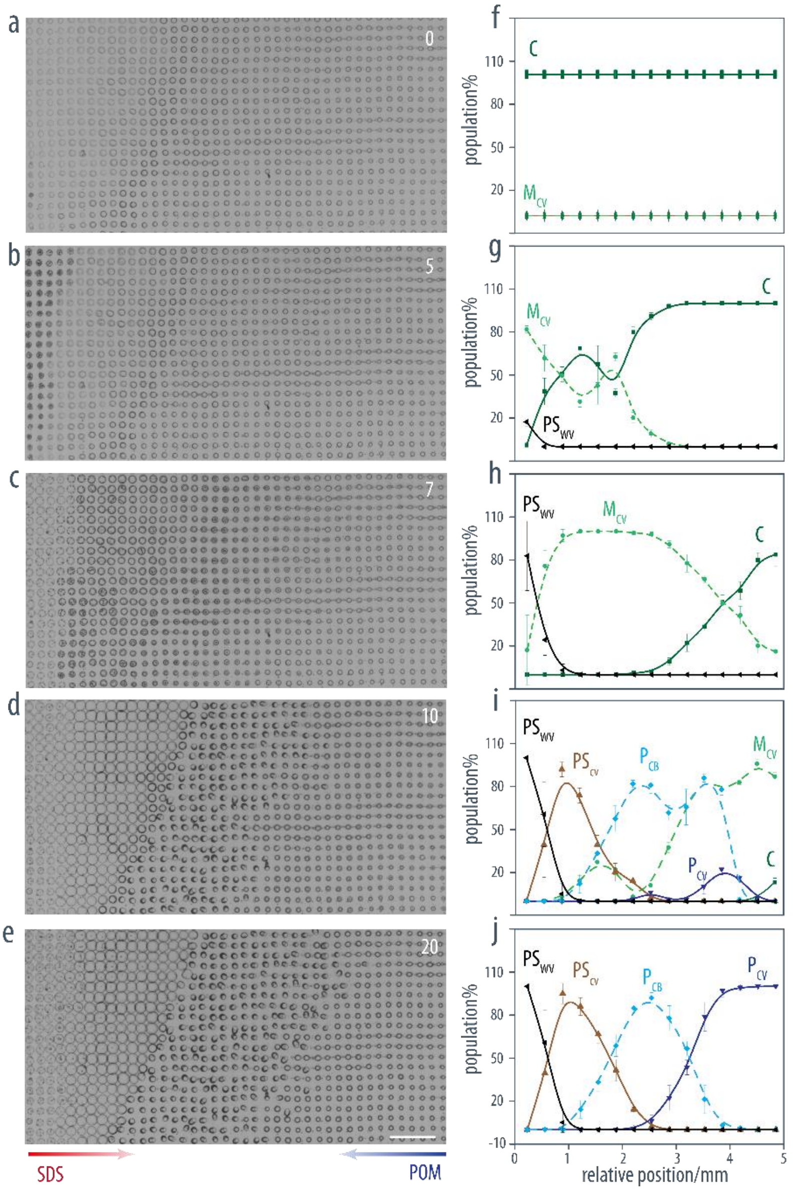

Supplementary Figure 31. (**a-e**) Time-dependent optical microscopy images of a 2D PDDA/ATP coacervate micro-droplet array recorded at t=0 (**a**), and 5 (**b**), 7 (**c**), 10 **(d**) and 20 min (**e**) after injection of counterflowing SDS and POM gradients prepared with an initial SDS : POM molar ratio of 2.3 (70/30 µL; 50 mM). (**f-j**) Plots of normalized population dynamics associated with the various forms of protocell differentiation shown in (**a-e**) respectively. Labels as denoted previously. Scale bar = 500 µm. Source data are provided as a Source Data file. Error bars represent the standard deviation of the statistics count of the different lines of different protocells (n = 3).

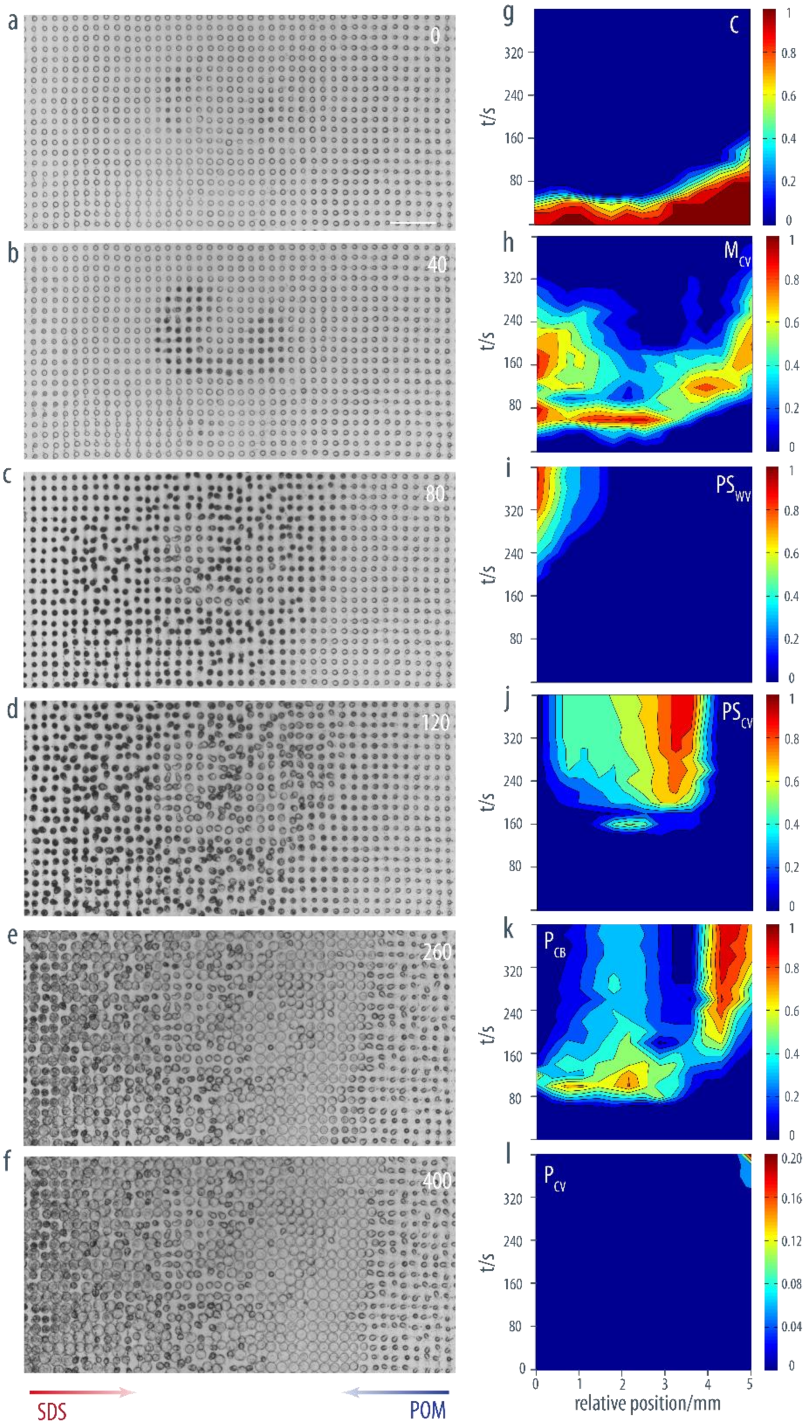

Supplementary Figure 32. (**a-f**) Time-dependent optical microscopy images of a 2D PDDA/ATP coacervate micro-droplet array recorded at t=0 (**a**), and 40 (**b**), 80 (**c**), 120 (**d**), 260 (**e**) and 400 s (**f**) after injection of counterflowing SDS and POM gradients prepared with an initial SDS : POM molar ratio of 9.0 (90/10 µL; 50 mM). (**g-l**) 2D plots of the spatiotemporal distributions of native coacervate droplets (**C**, (**g**)) and differentiated protocells **M_{CV}** (**h**), **PS_{WV}** (**i**), **PS_{CV}** (**j**), **P_{CB}** (**k**), and **P_{CV}** (**l**) in the 2D array. Scale bar = 500 µm. Source data are provided as a Source Data file.

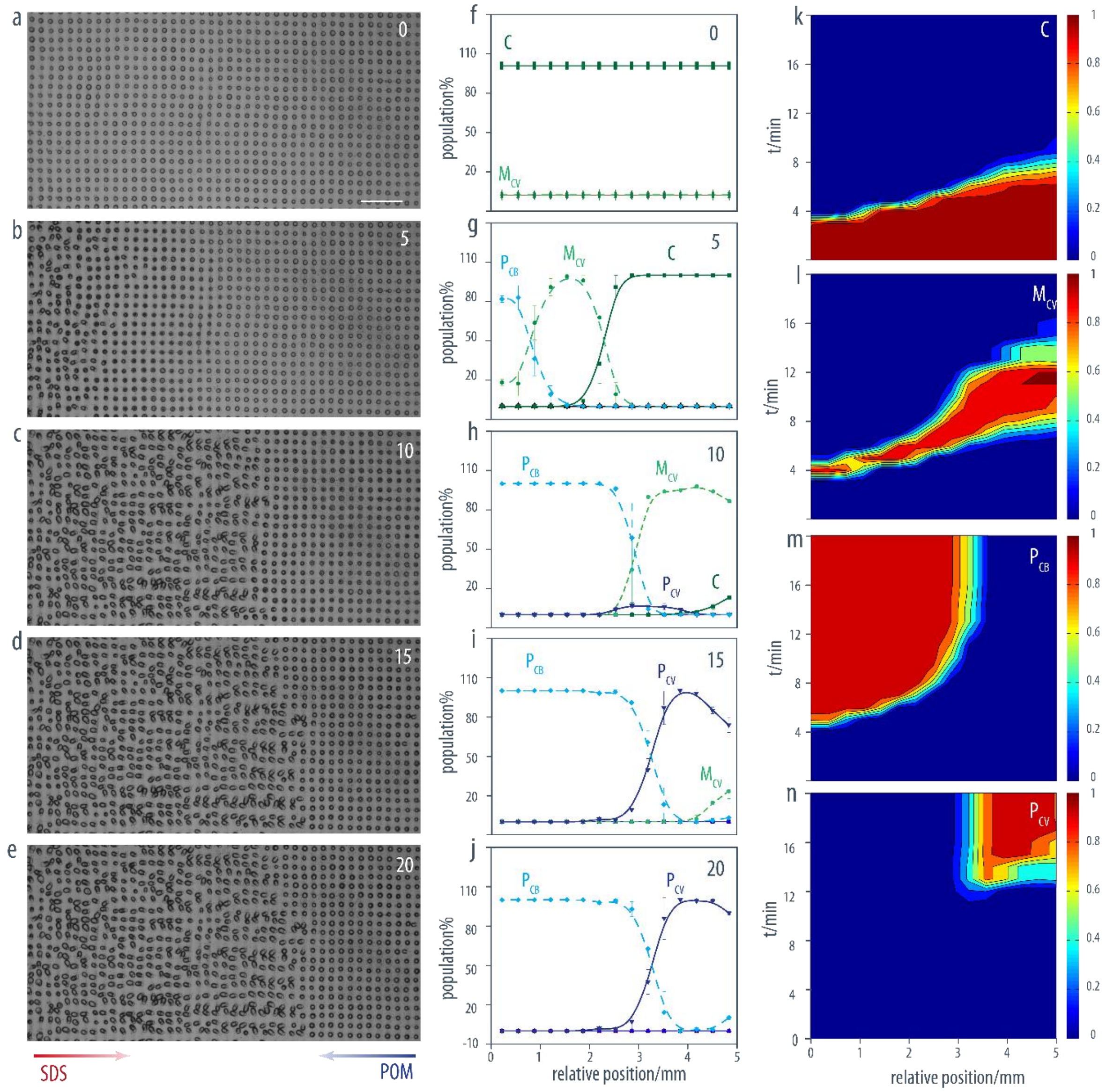

Supplementary Figure 33. (**a**-**e**) Time-dependent optical microscopy images of a 2D PDDA/ATP coacervate micro-droplet array recorded at t=0 (**a**), and 5 (**b**), 10 (**c**), 15 (**d**), and 20 min (**e**) after injection of counterflowing SDS and POM gradients prepared with an initial SDS : POM molar ratio of 1.0 (50/50 µL; 50 mM). (**f**-**j**) Plots of normalized population dynamics associated with the various forms of protocell differentiation shown in (**a**-**e**) respectively. Labels as denoted previously. Scale bar = 500 µm. (**k**-**n**) 2D plots of the spatiotemporal distributions of native coacervate droplets (**C**, (**k**)) and differentiated protocells $\mathbf{M_{CV}}$ (**l**), $\mathbf{P_{CB}}$ (**m**), and $\mathbf{P_{CV}}$ (**n**) in the 2D array. Source data are provided as a Source Data file. Error bars represent the standard deviation of the statistics count of the different lines of different protocells (n = 3).

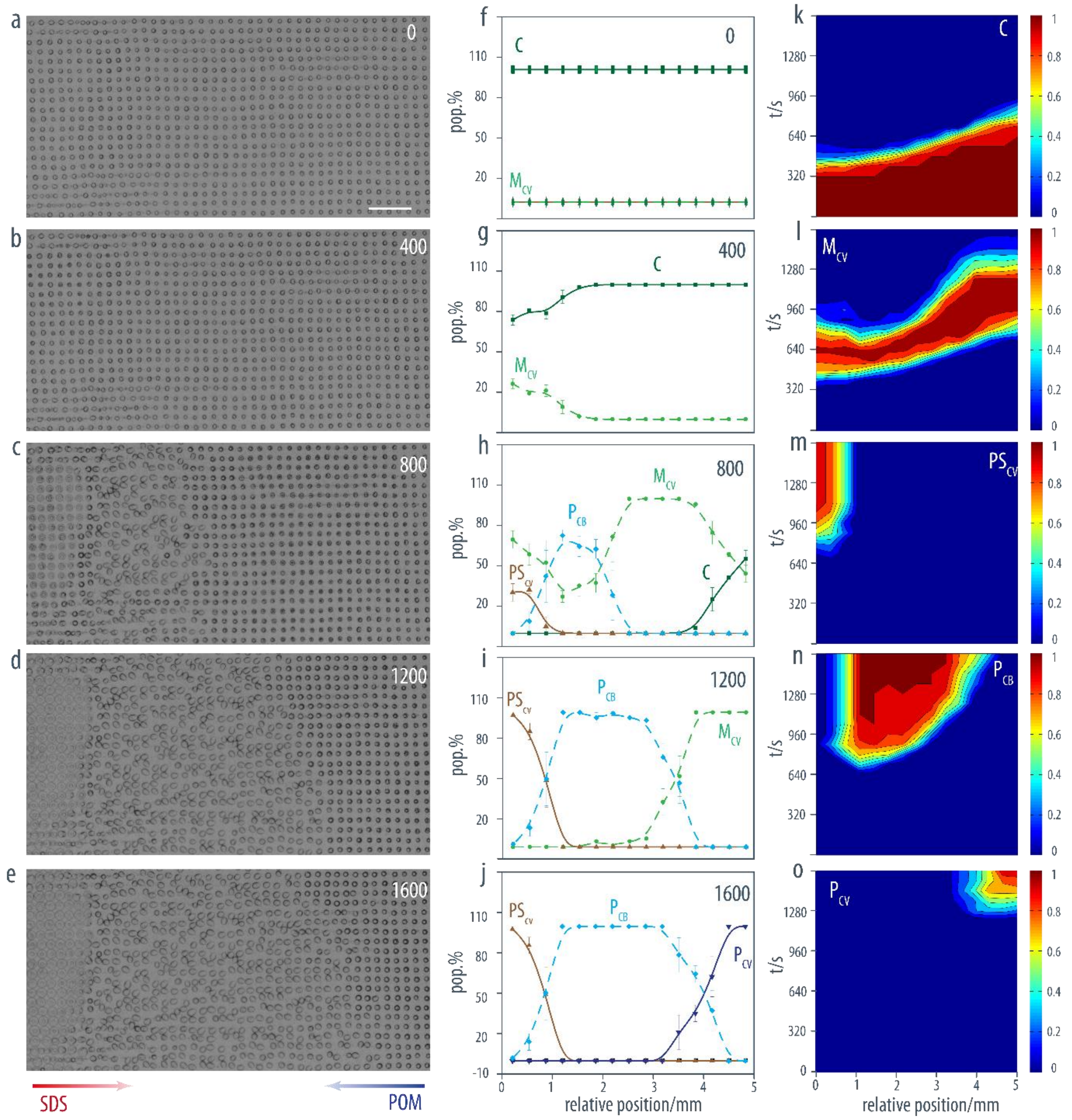

Supplementary Figure 34. (**a-e**) Time-dependent optical microscopy images of a 2D PDDA/ATP coacervate micro-droplet array recorded at t=0 (**a**), and 400 (**b**), 800 (**c**), 1200 (**d**), and 1600 s (**e**) after injection of counterflowing SDS and POM gradients prepared with an initial SDS : POM molar ratio of 0.43 (30/70 µL; 50 mM). (**f-j**) Plots of normalized population dynamics associated with the various forms of protocell differentiation shown in (**a-e**) respectively. Labels as denoted previously. Scale bar = 500 µm. (**k-o**) 2D plots of the spatiotemporal distributions of native coacervate droplets (**C**, (**k**)) and differentiated protocells **M$_{CV}$** (**l**), **PS$_{CV}$** (**m**), **P$_{CB}$** (**n**), and **P$_{CV}$** (**o**) in the 2D array. Source data are provided as a Source Data file. Error bars represent the standard deviation of the statistics count of the different lines of different protocells (n = 3).

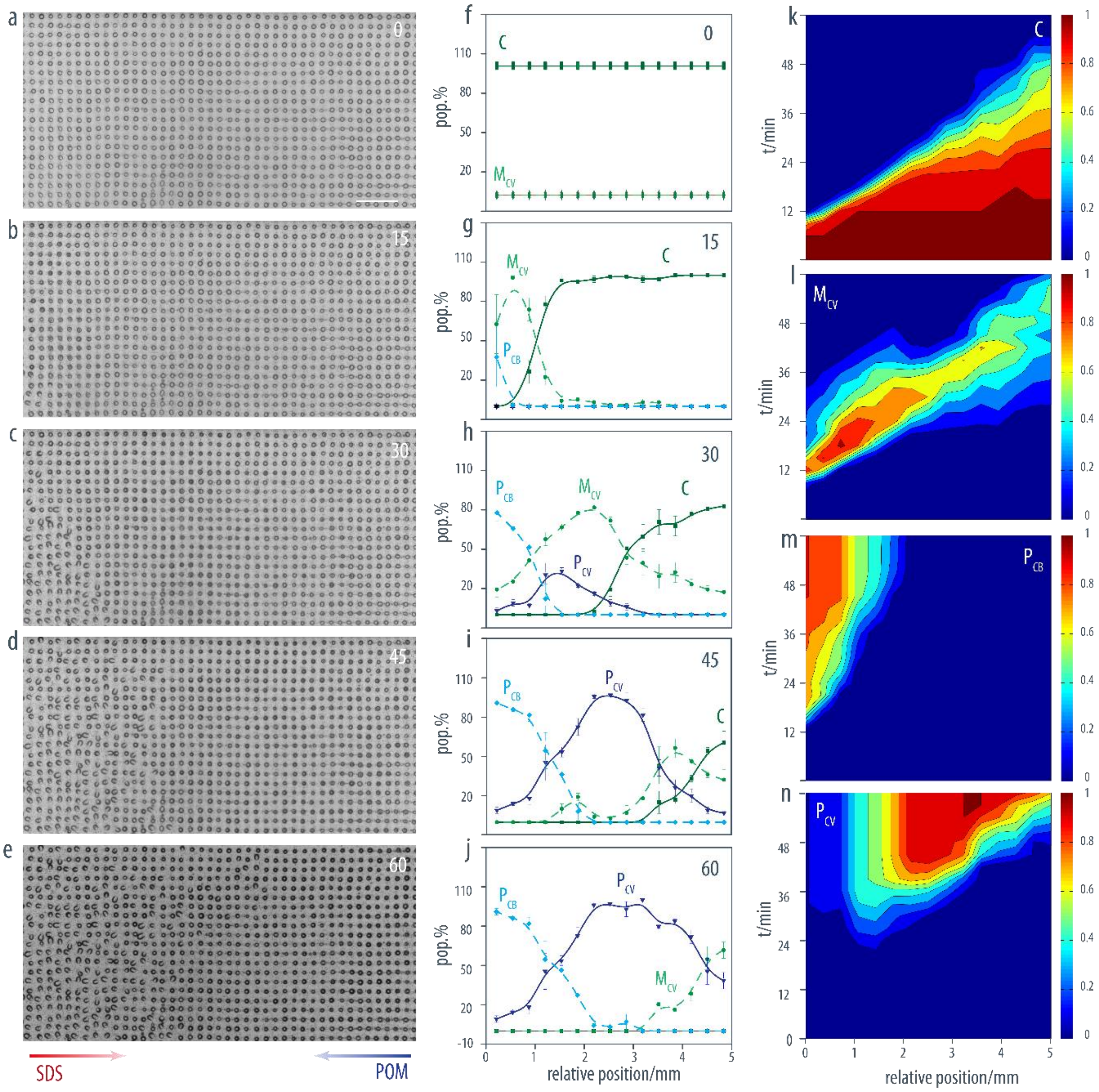

Supplementary Figure 35. (**a-e**) Time-dependent optical microscopy images of a 2D PDDA/ATP coacervate micro-droplet array recorded at t=0 (**a**), and 15 (**b**), 30 (**c**), 45 (**d**), and 60 min (**e**) after injection of counterflowing SDS and POM gradients prepared with an initial SDS : POM molar ratio of 0.11 (10/90 μL; 50 mM). (**f-j**) Plots of normalized population dynamics associated with the various forms of protocell differentiation shown in (**a-e**) respectively. Labels as denoted previously. Scale bar = 500 μm. (**k-n**) 2D plots of the spatiotemporal distributions of native coacervate droplets (**C**, (**k**)) and differentiated protocells **M_{CV}** (**l**), **P_{CB}** (**m**), and **P_{CV}** (**n**) in the 2D array. Source data are provided as a Source Data file. Error bars represent the standard deviation of the statistics count of the different lines of different protocells (n = 3).

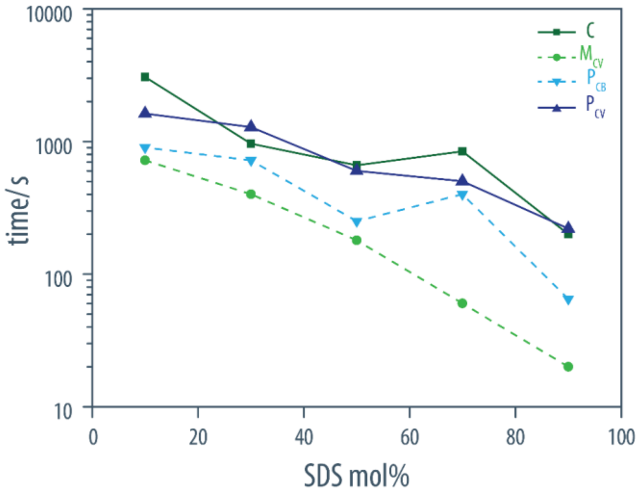

Supplementary Figure 36. Plots showing the time required for complete transformation of the coacervate droplet population (**C**, green line, squares) and appearance of **M**$_{CV}$ (light green, dashed line), **P**$_{CB}$ (light blue, dashed line) and **P**$_{CV}$ (blue line) differentiated morphological forms in opposing SDS/POM concentration gradients prepared at different initial mole% of SDS. Source data are provided as a Source Data file.

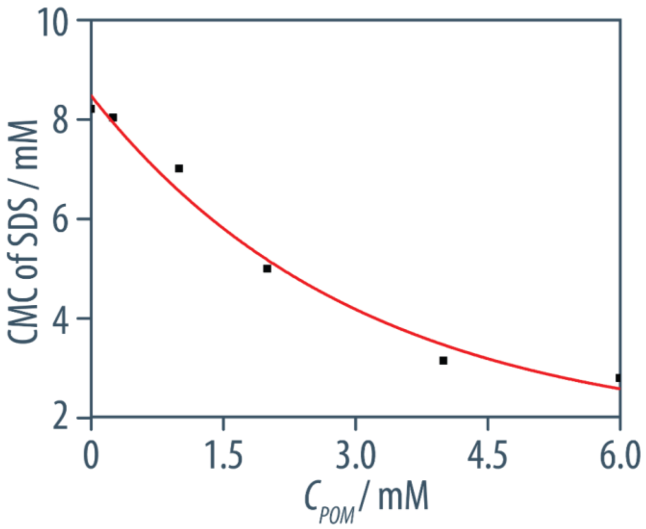

Supplementary Figure 37. Plot showing experimental values of SDS critical micelle concentration as measured against POM concentration. The CMC of SDS gradually decreases with increasing POM concentration. This is attributed to repulsion between the charged head groups which decreases with an increase of salt (POM) concentration, thereby facilitating micelle assembly at lower SDS monomer concentrations. The plot is fitted to a single exponential decay (see Supplementary Equation 5 in Supplementary Methods) with a R-square of 0.973. Source data are provided as a Source Data file.

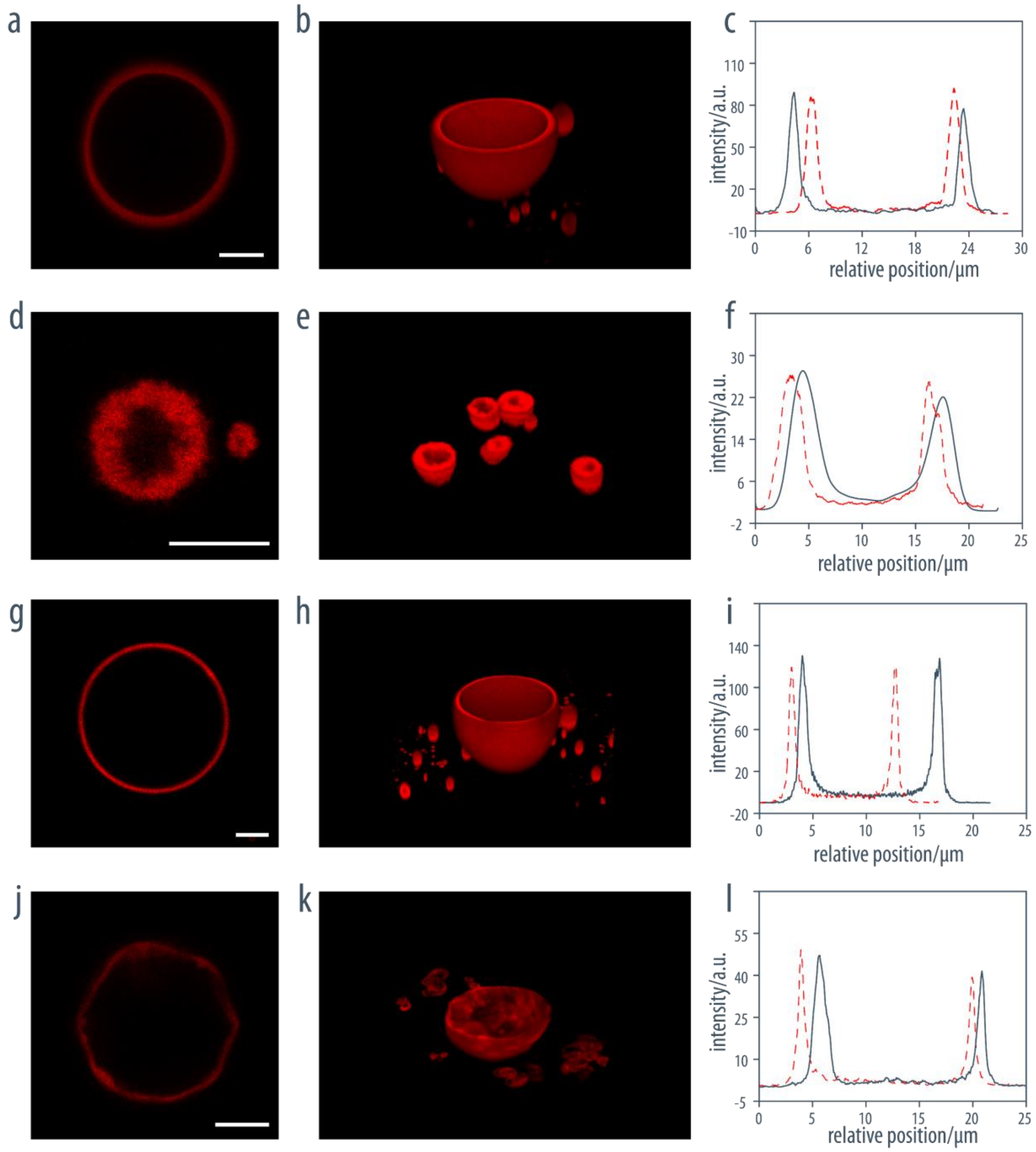

Supplementary Figure 38. (**a**,**b**) Confocal fluorescence microscopy (**a**) and 3D re-construction image (**b**) of a single POM/PDDA coacervate vesicle (**P**$_{CV}$) with encapsulated RITC-HRP (red fluorescence). The Z-stack image shows a water lumen and indicates that the RITC-HRP is preferentially localized in the outer membrane and coacervate sub-membrane layer (**c**) Line profiles comparing fluorescence intensities of RITC-HRP before (solid line) and after (dashed line) dilution, showing minimal loss of the encapsulated enzymes after 100-fold dilution. (**d-f**) As for (**a-c**) but for a single SDS/PDDA vesicle (**S**$_{V}$) with encapsulated RITC-HRP localized in the membrane. (**g-i**) As for (**a-c**) but for a single POM/SDS/PDDA vesicle (**PS**$_{CV}$) with encapsulated RITC-HRP localized in the membrane. (**j-l**) As for (**a-c**) but for a single POM/SDS/PDDA vesicle (**PS**$_{WV}$) with encapsulated RITC-HRP localized in the membrane. All scale bars = 5 µm. Source data are provided as a Source Data file.

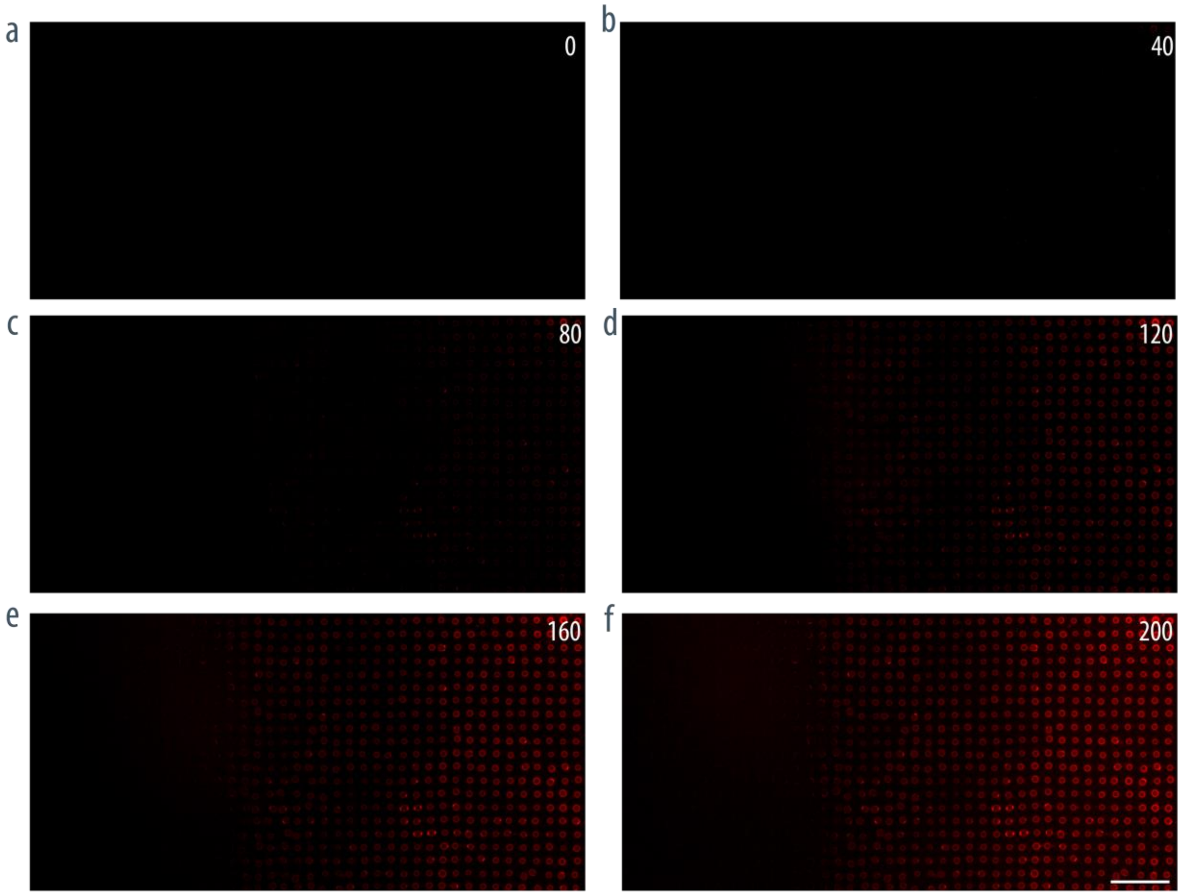

Supplementary Figure 39. (**a-f**) Time-dependent fluorescence microscopy images of a 2D array of HRP-containing differentiated protocells comprising a spatially separated tetramodal distribution of $\mathbf{PS_{WV}}$, $\mathbf{PS_{CV}}$, $\mathbf{P_{CB}}$ and $\mathbf{P_{CV}}$ (from left to right) morphological forms. Images are recorded at 0 (**a**), 40 (**b**), 80 (**c**), 120 (**d**), 160 (**e**) and 200 s (**f**) after injection of a mixture of Amplex red and H_2O_2 followed by vigorous stirring to achieve homogenous concentrations (final concentrations in the chamber, 2.5 and 10 µM, respectively) of the substrates across the 2D array. Formation of the product, resorufin, gives rise to red fluorescence specifically in the $\mathbf{P_{CV}}$ and $\mathbf{P_{CB}}$ populations but not in the domains containing the $\mathbf{PS_{WV}}$ and $\mathbf{PS_{CV}}$ protocells, which remain dark. The differentiated protocells were prepared using opposing gradients of SDS and POM (SDS : POM = 2.3 (70/30 µL; 50 mM). Scale bar 500 µm.

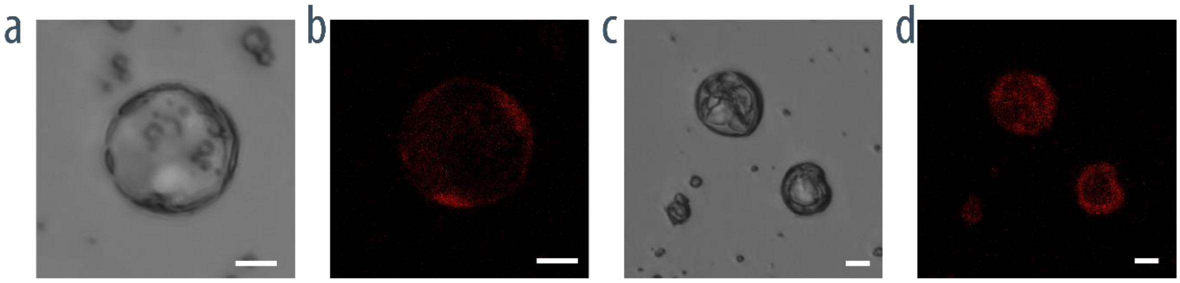

Supplementary Figure 40. (**a-d**) Optical (**a,c**) and corresponding fluorescence microscopy (**b,d**) images of a single HRP-containing **PS$_{CV}$** (**a,b**) or HRP-containing **PS$_{WV}$** (**c,d**) after exposure to aqueous solutions of H_2O_2 and Amplex red. The fluorescence microscopy images (**b,d**) were taken 30 min after addition of the substrates. In both cases, the protocells show low levels of red fluorescence associated with resorufin production inside the vesicles, indicating slow diffusion of Amplex red into the differentiated coacervate droplets. HRP-containing **PS$_{CV}$** and **PS$_{WV}$** were prepared by adding 500 µL of a SDS/POM (10/2 or 20/4 mM) mixture to a HRP (0.02 mg mL^{-1})-containing PDDA/ATP coacervate suspension (500 µL, 10 mM). After 30 min, the samples were centrifuged three times at 5000 rpm for 5 min to remove the supernatant and re-dispersed in Milli-Q water. 10 µL of the suspensions were then added to 200 µL of pre-mixed H_2O_2 and Amplex red solution (final concentrations, 10 µM and 2.5 µM, respectively). All scale bars, 10 µm.

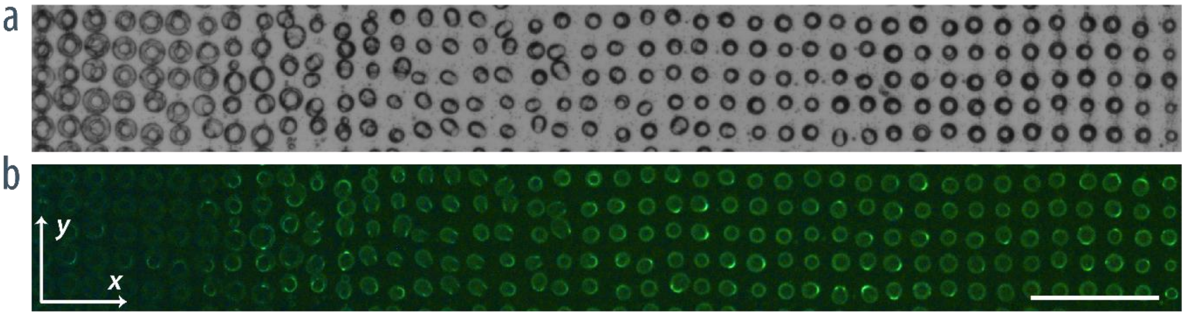

Supplementary Figure 41. (**a,b**) Optical (**a**) and corresponding fluorescence microscopy (**b**) images of 2D array of HRP-containing differentiated protocells comprising a spatially separated tetra-modal distribution of **PS$_{WV}$**, **PS$_{CV}$**, **P$_{CB}$** and **P$_{CV}$** (from left to right) morphological forms. The fluorescence image is recorded 5 min after injection of a mixture of *o*-phenylenediamine (*o*-PD) and H_2O_2 followed by vigorous stirring to achieve homogenous substrate concentrations (final concentrations in the chamber, 0.25 and 0.5 mM, respectively) across the 2D array. Formation of the product, 2,3-diaminophenazine (2,3-DAP) gives rise to yellow/green fluorescence in all the populations; scale bar, 500 µm.

a
b
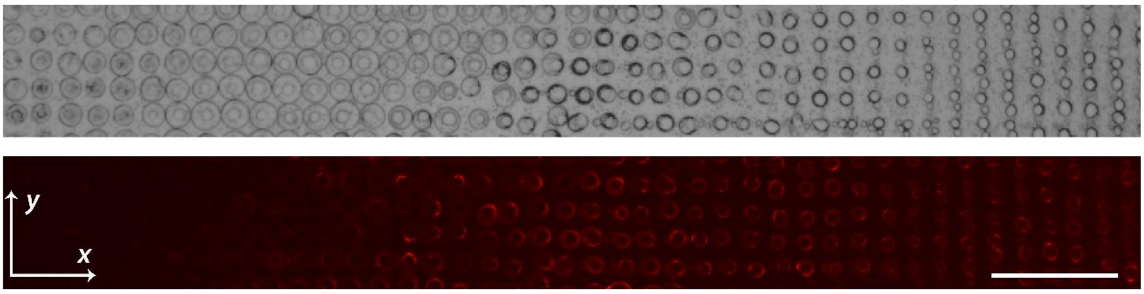

Supplementary Figure 42. (**a**,**b**) Optical (**a**) and corresponding fluorescence microscopy (**b**) images of a 2D array of HRP-containing differentiated protocells comprising a spatially separated tetra-modal distribution of $\mathbf{PS_{WV}}$, $\mathbf{PS_{CV}}$, $\mathbf{P_{CB}}$ and $\mathbf{P_{CV}}$ (from left to right) morphological forms. The fluorescence image is recorded 15 min after injection of resorufin by vigorous stirring to achieve a homogenous distribution of the fluorescent molecule (final concentration: 0.2 µM) across the 2D array. Scale bar, 500 µm.

Supplementary Methods

Materials

All chemicals were used without further purification. Adenosine 5′-triphosphate (ATP), sodium dodecyl sulfate (SDS), poly(diallyldimethylammonium chloride) (PDDA, 20 wt%, M_w ≈ 100-200 kDa), poly(allylamine hydrochloride) (PAH, M_w ≈ 17.5 kDa), sodium phosphotungstate (polyoxometalate; POM), rhodamine isothiocyanate (RITC), horseradish peroxidase (HRP), methylene blue, Nile red and pyranine were purchased from Sigma. 2′,3′-*O*-(2,4,6-trinitrophenyl)adenosine-5′-triphosphate (TNP-ATP, 10 mM, aqueous solution) was obtained from Jena Bioscience. 2-Methoxy(polyethyleneoxy)propyl trimethoxysilane (PEG-TMS) was purchased from ABCR GmbH. Amplex red was obtained from Thermo Fisher Scientific Inc. Hydrogen peroxide (H_2O_2) was purchased from VWR. Milli-Q water (18.2 MΩ·cm) was used for the preparation of all aqueous solutions.

Optical and confocal microscopy

Optical microscopy experiments were carried out using a Leica DMI 3000B optical microscope. Fluorescence imaging was performed using a Leica DFC 310FX, and dye molecules were excited by using specific filters with the following excitation (λ_{ex}) and emission wavelength cut offs (λ_{em}):, TNP-ATP, λ_{ex} = 355 - 425 nm and λ_{em} = 455 nm; pyranine, λ_{ex} = 450 - 490 nm and λ_{em} = 510 nm; RITC, resorufin, sulforhodamine B, Nile red, λ_{ex} = 515 - 560 nm and λ_{em} = 580 nm; methylene blue, λ_{ex} = 590 - 650 nm and λ_{em} = 660 nm. All glass slides used for the imaging were functionalized with PEG-TMS.

Confocal microscopy imaging was performed by mounting the custom-made acoustic device on a Leica SP5-II laser scanning microscope attached to a Leica DMI 6000 inverted epifluorescence microscope and equipped with a ×10 or ×20 objective (0.4 NA and 0.7 NA, respectively). High contrast images against the background solution were obtained after *ca*. 45 minutes of *in situ* pattern formation. 3D reconstructions were processed with Icy software, and all images were consistent with the images shown in the main text. All glass slides used for imaging were functionalized with PEG-TMS.

Labelling of horseradish peroxidase

RITC-labelled HRP was prepared by dissolving the enzyme (10 mL, 4 mg/mL) in sodium carbonate buffered solutions (100 mM, pH 8), followed by addition of a dimethyl sulfoxide (DMSO) solution of RITC (200 µL, 2 mg/mL). The reaction mixture was kept at 4 °C for 12 h, and then dialysed (molecular weight cut 15 kDa) against Milli-Q water over three days with regular changes in water. The fluorescently tagged enzyme was lyophilized and stored in the dark before use. UV/vis spectroscopy ($\varepsilon_{(559\ nm)} = 6.21 \times 10^4\ M^{-1}\ cm^{-1}$ for RITC) typically gave a RITC : HRP molar ratio of 1 : 40.

RITC functionalization of PAH

PAH (10 mL, 10 mg/mL) was dissolved in 4-(2-hydroxyethyl)-1-piperazinepropanesulfonic acid buffer (EPPS, 100mM, pH 9.5), and then a DMSO solution of RITC (2.83 mL, 1 mg/mL) slowly added. The reaction mixture was stirred in the dark for 12 h, and then dialysed (molecular weight cut-off 7 kDa) against Milli-Q water over three days with regular changes in water. The RITC-tagged PAH polymer was lyophilized and stored in the dark before use. UV/vis spectroscopy ($\varepsilon_{(559\ nm)} = 6.21 \times 10^4\ M^{-1}\ cm^{-1}$ for RITC) typically gave a RITC : PAH monomer molar ratio of 1 : 10^4.

Mapping of the morphological landscape

To determine the range of possible morphological types produced by the interaction of POM or SDS, or mixtures of the two additives on preformed membrane-free PDDA/ATP coacervate droplets, a matrix of 30 samples was prepared and investigated. In each case, the coacervate micro-droplets were prepared by adding ATP (1 mL, 50 mM) to a solution of PDDA (10 mL, 5 mM, monomer, 100 - 200 kDa), followed by centrifugation of the suspensions at 2000 rpm for 5 min, and removal of the supernatant. The coacervate phase was then re-dispersed in 5 mL of Milli-Q water and solutions of the morphogens (total volume, 500 µL) added to 500 µL of the re-dispersed micro-droplets to give the following five sets of experiments with the following final concentrations (*C*): **A1**-**A6**; C_{POM} = 0 mM, C_{SDS} = 0-10 mM; **B1**-**B6**, C_{POM} = 0.5 mM, C_{SDS} = 0-10 mM; **C1**-**C6**, C_{POM} = 1.0 mM, C_{SDS} = 0-10 mM; **D1**-**D6,** C_{POM} = 1.5 mM, C_{SDS} = 0-10 mM; and **E1**-**E6**, C_{POM} = 2.0 mM, C_{SDS} = 0-10 mM. The resulting membrane-bounded protocells were classified according to five different morphological types using optical and fluorescence microscopy. Fluorescent coacervate droplets were prepared by doping the PDDA/ATP mixtures with TNP-ATP or RITC-PAH.

Determination of CMC of SDS at different POM concentration

The CMC values of SDS at different concentrations of POM were determined by using pyrene as a fluorescent probe. The measurements were performed on a Fluoromax 4 fluorescence spectrometer with excitation wavelength of 334 nm and the emission spectrum was recorded from 350 to 450 nm; the excitation/emission slits were set as 4/2 mm. For each measurement, 1 µL of pyrene/ethanol solution (0.66 mM) was added to 1 mL of SDS and SPT solution. The ratio (I_3/I_1) between the intensities of the first (I_1=372 nm) peak and the third (I_3=383 nm) peak in the fluorescence emission spectra was used to determine the CMC of SDS.

Simulation methods

The simulated area consisted of the entire chamber (20 x 20 mm). Results are shown corresponding to the 5x5 mm square in the centre of the device (observation window; see Supplementary Figures 14 and 15). The simulations assumed that the chemical morphogens (POM or SDS) were injected from the left-hand side of the device and diffused along the *x* direction. In general, diffusion of SDS and POM was restricted to a 2D plane to simulate addition of the morphogens at the base of the acoustic trapping device (20 x 20 x 2 mm). Restricting the diffusion plane to a height (***z***) of 500 µm gave simulated induction times (*ca.* 3 min) commensurate with the experimental data for known injected morphogen concentrations. In contrast, larger values of ***z*** (2 mm) gave simulated induction times of around 15-30 min.

The simulated concentration gradient across the viewing area along the diffusion direction was defined as; $\Delta C = C_{in} - C_{out}$. The 2D diffusion profiles of POM were directly determined using Fick's equations:

$$\frac{\partial[POM]}{\partial t} = D(POM)\left(\frac{\partial^2[POM]}{\partial x^2} + \frac{\partial^2[POM]}{\partial y^2}\right) \qquad (1)$$

where the diffusion coefficient of POM (*D(POM)*) is 2.48 x 10^{-10} m^2 s^{-1} (Ref 1), *t* signifies time, and *x* and *y* are dimensions along and perpendicular to the diffusion direction, respectively (see Figure 14a). Simulations included: (i) plots of ΔC against time across the observation window and along the diffusion direction, and (ii) 2D plots of the spatial and temporal distributions of the POM concentration in a row of protocells aligned perpendicular or parallel to the diffusion direction in the centre of the viewing window (plots showing changes in concentration with time as a function of relative position in the row). The simulations were approximations as they did not consider binding of the POM clusters at the coacervate surface or morphogen depletion during the reaction-diffusion process.

Simulation of the SDS gradients was complicated by the continuous interchange of SDS between molecular dispersed monomers and self-assembled micelles (critical micelle concentration, C_{CMC}= 8.2 mM; 62 molecules per micelle) (Ref. 2). The diffusion coefficients of the SDS molecule (C_{SDS}^{Mol}) and SDS micelle (C_{SDS}^{Mic}) were 5.3 x 10^{-10} m^2 s^{-1} (Ref.2), and 9.2 x 10^{-11} m^2 s^{-1} (Ref. 3), respectively. Diffusion of both the molecular and micellular components were modelled using a finite-difference solution to the 2D Fick's diffusion equation. We assumed instant inter-conversion of SDS molecules and micelles during the diffusion process such that the interchange was determined by assessing the total surfactant concentration relative to the CMC. Hence, at each time step (typically 3s), the total SDS concentration at each location in the observation window was evaluated relative to the CMC and the concentration partitioned into molecular and micellular species according to:

$$if\ C_{SDS}^{Tot} \geq C_{CMC}\ then\ C_{SDS}^{Mol} = C_{CMC}\ and\ C_{SDS}^{Mic} = C_{SDS}^{Tot} - C_{CMC} \qquad (2)$$

and

$$if\ C_{SDS}^{Tot} < C_{CMC}\ then\ C_{SDS}^{Mol} = C_{SDS}^{Tot} \qquad (3)$$

After partition, simulations of the diffusion gradients of the molecular and micellular components were undertaken by separate application of the finite-difference method for a discrete time step, after which the evaluation process was repeated. To ensure the stability of the simulation, the stability factor (F_o) was set to less than 0.25,

$$F_O = \frac{D(S) \times \Delta t}{L^2} \leq 0.25 \qquad (4)$$

where *L* was 100 µm (representing one step of the lattice) and Δt was set as 3 s.

Simulation of opposing concentration gradients of POM and SDS along the *x* direction of an array of model droplets was undertaken using Fick's equations (Supplementary Equation 1) to directly determine the diffusion profiles of POM under different conditions. As the presence of POM decreased the CMC of SDS, we used the simulated spatially distribution of POM to dynamically modulate the localized conversion of SDS micelles into SDS monomers along the morphogen gradients. For this, we used experimental CMC values (C_{CMC}) that were determined under equilibrium conditions in the presence of a range of POM concentrations (C_{POM}) using pyrene as a fluorescent probe (see Supplementary Methods). The data were

fitted to a single exponential decay function (Supplementary Figure 38) with the following parameters:

$$C_{CMC} = 6.853 \times e^{(-\frac{C_{POM}}{3.036})} + 1.617 \quad (5)$$

Using a finite explicit approach, dynamic adjustments of the CMC of SDS in the POM concentration gradient were calculated in the same time interval (3 s) and the adjusted values inserted into the chemical concentration gradients and then iterated into the next round of calculations.

Supplementary References

1. P. Stonehart, Diffusion coefficients of tungsten heteropolyacids. *Anal. Chim. Acta* 37, 1967, 350-358.
2. T. Liu, R. Guo, G. Song, Determination of the diffusion coefficient for SDS micelle with different shape and the effects of ethanol by cyclic voltammetry without probes. *J. Dispersion Sci. Technol.17,* **1996,** 509-526.
3. N. Kamenka, B. Lindman, B. Brun, Translational motion and association in aqueous sodium dodecyl sulphate solutions. *Colloid Polymer Sci. 252*, **1974**, 144-152.
