# Supplementary material for: Artificial morphogen-mediated differentiation in synthetic protocells
Source: Nat Commun. 2019 Jul 25;10:3321. doi: 10.1038/s41467-019-11316-4 (PMC6658542; doi:10.1038/s41467-019-11316-4)
Supplement: Supplementary file 13 — Description of Additional Supplementary Files [file 41467_2019_11316_MOESM13_ESM.docx]

**Title: Supplementary Movie 1**

**Description:** Morphological transformations in coacervate droplet arrays under non-diffusive equilibrium conditions at a POM final concentration of 2 mM. Optical microscopy video showing the transformation of acoustically formed membrane-free PDDA/ATP coacervate droplets into membrane-bounded POM/coacervate vesicles (**P_CV_**). An aqueous solution of POM clusters (sodium phosphotungstate; 500 µL, 4 mM) was injected into a 2D coacervate micro-droplet array with vigorously stirring to ensure homogeneous mixing. The droplet array was prepared by addition of ATP (100 µL, 50 mM) to a PDDA (1 mL, 5 mM monomer, 100-200 kDa) solution contained within the sample chamber of an acoustic trapping device constructed with two transducer pairs operating at 6.76/6.78 MHz (10 V). The movie is shown at x30 of real-time speed at 6 frames per second. Total duration of recording was 5 min in real time.

**Title: Supplementary Movie 2**

**Description:** Morphological transformations in coacervate droplet arrays under non-diffusive equilibrium conditions at a POM final concentration of 0.5 mM. Optical microscopy video showing the transformation of acoustically formed membrane-free PDDA/ATP coacervate droplets into multi-compartmentalized POM/coacervate vesicles (**M_CV_**). An aqueous solution of POM clusters (sodium phosphotungstate; 500 µL, 1 mM) was injected into a 2D coacervate micro-droplet array with vigorously stirring to ensure homogeneous mixing. The droplet array was prepared as in Movie 1. Movie is shown at x100 of real-time speed at 6 frames per second. Total duration of recording was 15 min in real time.

**Title: Supplementary Movie 3**

**Description:** Morphological transformations in coacervate droplet arrays under non-diffusive equilibrium conditions at a POM final concentration of 20 mM. Optical microscopy video showing kinetic inhibition of the transformation of PDDA/ATP coacervate droplets into spherical POM/coacervate vesicles. An aqueous solution of POM clusters (sodium phosphotungstate; 500 µL, 40 mM) was injected into a 2D coacervate micro-droplet array with vigorously stirring to ensure homogeneous mixing. The droplet array was prepared as in Movie 1. Movie is shown at x90 of real-time speed at 6 frames per second. Total duration of recording was 15 min in real time.

**Title: Supplementary Movie 4**

**Description:** Morphological transformations in coacervate droplet arrays under non-diffusive equilibrium conditions at a POM final concentration of 1 mM. Optical microscopy video showing the transformation of acoustically formed membrane-free PDDA/ATP coacervate droplets into membrane-bounded POM/coacervate vesicles with balloon-like morphology vesicles (**P_CB_**), An aqueous solution of POM clusters (sodium phosphotungstate; 500 µL, 2 mM) was injected into a 2D PDDA/ATP coacervate micro-droplet array with vigorously stirring to ensure homogeneous mixing. The droplet array was prepared as in Movie 1. Movie is shown at x30 of real-time speed at 6 frames per second. Total duration of recording was 5 min in real time. The anisotropic form was attributed to the adhesion force between the coacervate micro-droplets and glass substrate, which under relative low concentrations of POM was sufficient to overcome the osmotically induced isotropic expansion of the POM/PDDA membrane surrounding the coacervate vesicles. As a consequence, whilst expansion away from the substrate remained unhindered, growth in the lateral direction was curtailed by membrane adhesion.

**Title: Supplementary Movie 5**

**Description:** Morphological transformations in coacervate droplet arrays under non-diffusive equilibrium conditions at a SDS final concentration of 20 mM. Optical microscopy video showing the transformation of acoustically formed membrane-free PDDA/ATP coacervate droplets into membrane-bounded SDS/PDDA vesicles (**S_V_**). An aqueous solution of SDS (500 µL, 40 mM) was injected into a 2D PDDA/ATP coacervate micro-droplet array with vigorously stirring to ensure homogeneous mixing. The droplet array was prepared as in Movie 1. Movie is shown at x50 of real-time speed at 10 frames per second. Total duration of recording was 8 min in real time.

**Title: Supplementary Movie 6**

**Description:** Protocell differentiation in unidirectional reaction-diffusion POM gradients. Optical microscopy video showing the spatiotemporal transformation of an array of homogeneous PDDA/ATP coacervate droplets into a binary population of spherical (**P_CV_**; left side) and balloon-shaped (**P_CB_**; centre and right side) membrane-bounded POM/coacervate vesicles. An aqueous solution of sodium phosphotungstate (50 µL, 12.5 mM) was injected into the 2D PDDA/ATP coacervate micro-droplet array from the left-hand side as viewed in the video. The droplet array was prepared as in Movie 1. Movie is shown at x30 of real-time speed at 6 frames per second. Total duration of recording was 5 min in real time.

**Title: Supplementary Movie 7**

**Description:** Protocell differentiation in unidirectional reaction-diffusion SDS gradients. Optical microscopy video showing the spatiotemporal transformation of an array of homogeneous PDDA/ATP coacervate droplets into a ternary population of ATP-depleted SDS/PDDA vesicles (**S_V_**; left side), multi-compartmentalized coacervate vesicles (**M_CV_**; centre) and native coacervate droplets (**C**; left). An aqueous solution of SDS (50 µL, 50 mM) was injected into the 2D PDDA/ATP coacervate micro-droplet array from the left-hand side as viewed in the video. The droplet array was prepared as in Movie 1. Movie is shown at x90 of real-time speed at 18 frames per second. Total duration of recording was 15 min in real time.

**Title: Supplementary Movie 8**

**Description:** Protocell differentiation in opposing reaction-diffusion SDS/POM gradients at an initial morphogen molar ratio of 2.3 (70/30 µL, 50 mM). Optical microscopy video showing the spatiotemporal transformation of an array of homogeneous PDDA/ATP coacervate droplets. The coacervate droplets differentiate into four spatially distinct populations (POM/SDS wrinkled vesicles (**PS_WV_**, far left), POM/SDS coacervate vesicles (**PS_CV,_** left), balloon-shaped POM/coacervate vesicles (**P_CB_**, centre) and spherical coacervate vesicles (**P_CV_**; right) are produced via multi-compartmentalized POM/coacervate vesicles (**M_CV_**). Aqueous solutions of SDS and POM are injected from the left and right sides of the device, respectively. The droplet array was prepared as in Movie 1. Movie is shown at x100 of real-time speed at 20 frames per second. Total duration of recording was 20 min in real time.

**Title: Supplementary Movie 9**

**Description:** Protocell differentiation in opposing reaction-diffusion SDS/POM gradients at a morphogen molar ratio of 9.0 (90/10 µL; 50 mM). Optical microscopy video showing the spatiotemporal transformation of an array of homogeneous PDDA/ATP coacervate droplets. The coacervate droplets differentiate into a spatially interpenetrating community of morphological types comprising a small region of POM/SDS/PDDA wrinkled vesicles (**PS_WV_**) that were positioned nearest the SDS gradient, an extensive central region containing changing numbers of **PS_WV_, PS_CV_** and **P_CB_** forms with **PS_CV_** being dominant throughout, and a demarcated area of **P_CV_** structures closest to the POM gradient. Aqueous solutions of SDS and POM are injected from the left and right sides of the device, respectively. The droplet array was prepared as in Movie 1. Movie is shown at x50 of real-time speed at 10 frames per second. Total duration of recording was 10 min in real time.

**Title: Supplementary Movie 10**

**Description:** Protocell differentiation in opposing reaction-diffusion SDS/POM gradients at a morphogen molar ratio of 1.0 (50/50 µL; 50 mM). Optical microscopy video showing the spatiotemporal transformation of an array of homogeneous PDDA/ATP coacervate droplets. The coacervate droplets differentiate into two spatially distinct populations (balloon-shaped POM/coacervate vesicles (**P_CB_**, left and centre) and spherical coacervate vesicles (**P_CV_**; right) are produced via multi-compartmentalized POM/coacervate vesicles (**M_CV_**). Aqueous solutions of SDS and POM are injected from the left and right sides of the device, respectively. The droplet array was prepared as in Movie 1. Movie is shown at x100 of real-time speed at 20 frames per second. Total duration of recording was 20 min in real time.

**Title: Supplementary Movie 11**

**Description:** Functional diversity in differentiated protocell consortia. Fluorescence microscopy video showing spatial localization of enzyme activity (increase in red fluorescence) in a morphogen-mediated differentiated protocell community. A tetramodal distribution of HRP-containing **PS_WV_**, **PS_CV_**, **P_CB_** and **P_CV_** protocells (from left to right) was prepared using opposing SDS/POM reaction-diffusion gradients (SDS : POM = 2.3 (70/30 µL; 50 mM). Aqueous solutions of SDS and POM are injected from the left and right sides of the device, respectively. The enzyme reaction is initiated by injection of a mixture of H_2_O_2_ and Amplex red (final concentrations in the chamber, 10 and 2.5 µM, respectively) followed by vigorous stirring to ensure homogeneous mixing. Fluorescence microscopy (λ_ex_ = 515 - 560 nm and λ_em_ = 580 nm) was used to detect the HRP-mediated conversion of non-fluorescence Amplex red to red fluorescent resorufin. Resorufin production occurs only in the **P_CB_** and **P_CV_** protocell populations. In contrast, Amplex red does not permeate the **PS** hybrid membrane such that the domains associated with **PS_WV_** and **PS_CV_** remain dark. Movie is shown at x30 of real-time speed at 15 frames per second. Total duration of recording was 6 min in real time.
